# Identification of α-Synuclein Proaggregator: Rapid Synthesis and Streamlining RT-QuIC Assays in Parkinson’s Disease

**DOI:** 10.1021/acsmedchemlett.2c00138

**Published:** 2022-08-11

**Authors:** Fumito Takada, Takahito Kasahara, Kentaro Otake, Takamitsu Maru, Masanori Miwa, Kei Muto, Minoru Sasaki, Yoshihiko Hirozane, Masato Yoshikawa, Junichiro Yamaguchi

**Affiliations:** † Department of Applied Chemistry, Waseda University, 513 Wasedatsurumakicho, Shinjuku, Tokyo 162-0041, Japan; ‡ Takeda Pharmaceutical Company Limited, 2-26-1 Muraoka-Higashi, Fujisawa, Kanagawa 251-8555, Japan; § Axcelead Drug Discovery Partners Inc., 2-26-1 Muraoka-Higashi, Fujisawa, Kanagawa 251-8555, Japan; ∥ Waseda Institute for Advanced Study, Waseda University, 513 Wasedatsurumakicho, Shinjuku, Tokyo 162-0041, Japan

**Keywords:** C−H Arylation, α-synuclein, proaggregator, RT-QuIC

## Abstract

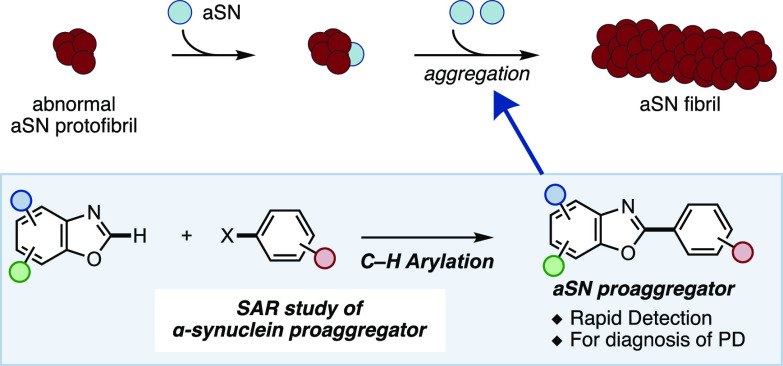

We report the discovery of two compounds, TKD150 and TKD152, that promote the aggregation of α-synuclein (aSN) using a real-time quaking-induced conversion (RT-QuIC) assay to detect abnormal aSN. By utilizing a Pd-catalyzed C–H arylation of benzoxazole with iodoarenes and implementing a planar conformation to the design, we successfully identified TKD150 and TKD152 as proaggregators for aSN. In comparison to a previously reported proaggregator, PA86, the two identified compounds were able to promote aggregation of aSN at twice the rate. Application of TKD150 and TKD152 to the RT-QuIC assay will shorten the inherent lag time and may allow wider use of this assay in clinical settings for the diagnosis of α-synucleinopathy-related diseases.

In a world with an aging population, neurodegenerative disorders such as Parkinson’s disease (PD) and dementia with Lewy bodies (DLB) has a prevalence of up to 1% of the population for those who are over the age of 60.^1^ Typical symptoms of the disease are involuntary movement of the body and rigidity of muscles, which results from the loss of dopaminergic neurons in substantia nigra pars compacta in the midbrain. A pathological hallmark of PD and DLB is the presence of Lewy bodies (LB) in a patient’s nerve cells. Although the mechanism of Lewy body formation and its pathogenesis remains elusive, it is well recognized that abnormal α-synuclein (aSN) such as insoluble aSN aggregates and aSN fibrils are the major components of LB.^2^ Detection and quantification of abnormal aSN that is present in a miniscule amount as a biomarker for disease prognosis has attracted attention, and the development of such technology holds potential for early diagnosis of PD and DLB.^3−5^

Recently, an application of a real-time quaking-induced conversion (RT-QuIC) assay for the detection of abnormal aSN present in the brain and cerebrospinal fluid of PD and DLB patients has been published (Figure 1).^6^ This assay is based on a phenomenon where agitation of aSN facilitates aggregation to form fibrils, which thioflavin T binds to and generates a fluorescence signal. The aggregation occurs faster in the presence of abnormal aSN, thus attracting interest for its application as a prognostic tool for synucleopathy-related diseases such as PD and DLB.^7−9^ Despite the potential utility of this methodology, the inherent lag time (70–140 h) to acquire the results has been a drawback of the RT-QuIC assay.^6,10^ The lag phase is attributed to the slow aggregation of abnormal aSN and requires a long time to accumulate sufficient fluorescence signal for detection. If the assay could be chemically expedited by using compounds to promote the aggregation process, the RT-QuIC assay would become more useful, especially in a clinical setting. Although several small molecules have been reported to inhibit the aggregation of aSN, a compound that functions as a proaggregator is much rarer. To our knowledge, only one report by the Otzen group demonstrated the ability of 2-arylbenzoxazole (PA86, **1**) to promote the aggregation of aSN.^11^ Inspired by Otzen’s finding, we saw an opportunity to further develop the compound’s ability to promote aSN aggregation. Herein we report the discovery of two compounds, TKD150 and TKD152, which can promote the aggregation of aSN monomers twice as fast as PA86.

**Figure 1:**
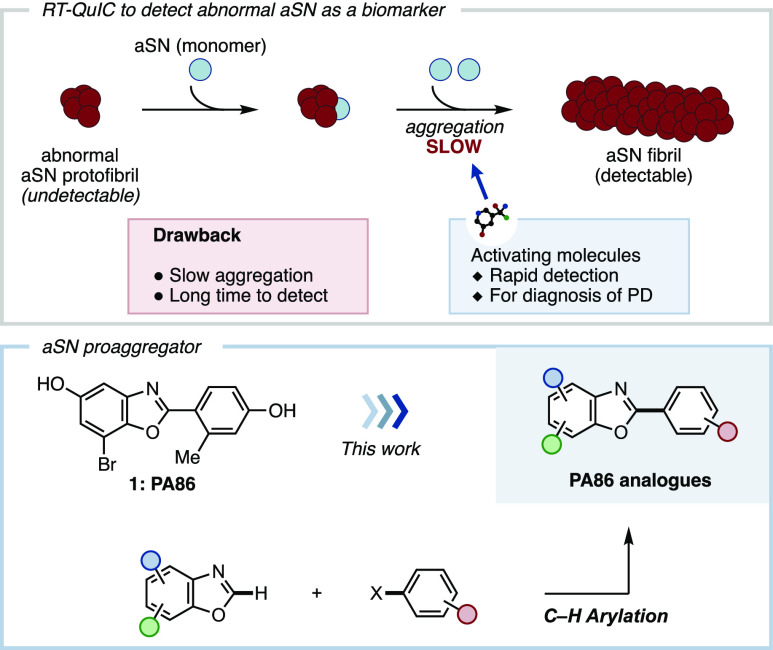
aSN as a biomarker for the diagnosis of PD and identification of aSN proaggregator by C–H arylation.

To carry out a rapid and diversified synthesis of PA86 analogues, we envisioned a C–H arylation strategy to construct the 2-aryl benzoxazole framework (Figure 1, bottom). The synthesis of PA86 has been reported in one patent in which a typical heterocycle synthesis through a condensation of 2-aminophenols with benzoyl chlorides, followed by cyclization was employed.^12^ While this synthesis would be applicable to evaluate and improve the biological activity of the compound, we sought for a shorter synthetic route as well as avoiding the use of moisture-sensitive acid chlorides.

We first investigated appropriate reaction conditions for the C–H arylation^13−20^ between benzoxazoles and haloarenes. With a bromo substituent present on the benzoxazole in PA86 (**1**), we sought for reaction conditions that suppresses undesired competitive reactions at the bromine atom. We noticed that Miura’s C–H arylation conditions using Pd(OAc)_2_/PPh_3_/Cs_2_CO_3_ catalysis showed high reactivity for iodoarenes compared with bromoarenes.^21^ With a slight modification of Miura’s C–H arylation conditions, we succeeded in assembling benzoxazoles with iodoarenes to deliver a variety of 2-aryl benzoxazoles without the loss of the bromine atom (Figure 2A). After this C–H arylation, demethylation of the methoxy groups furnished of PA86 (**1**) and a variety of analogues **2**–**16** (see the Supporting Information for details).

**Figure 2:**
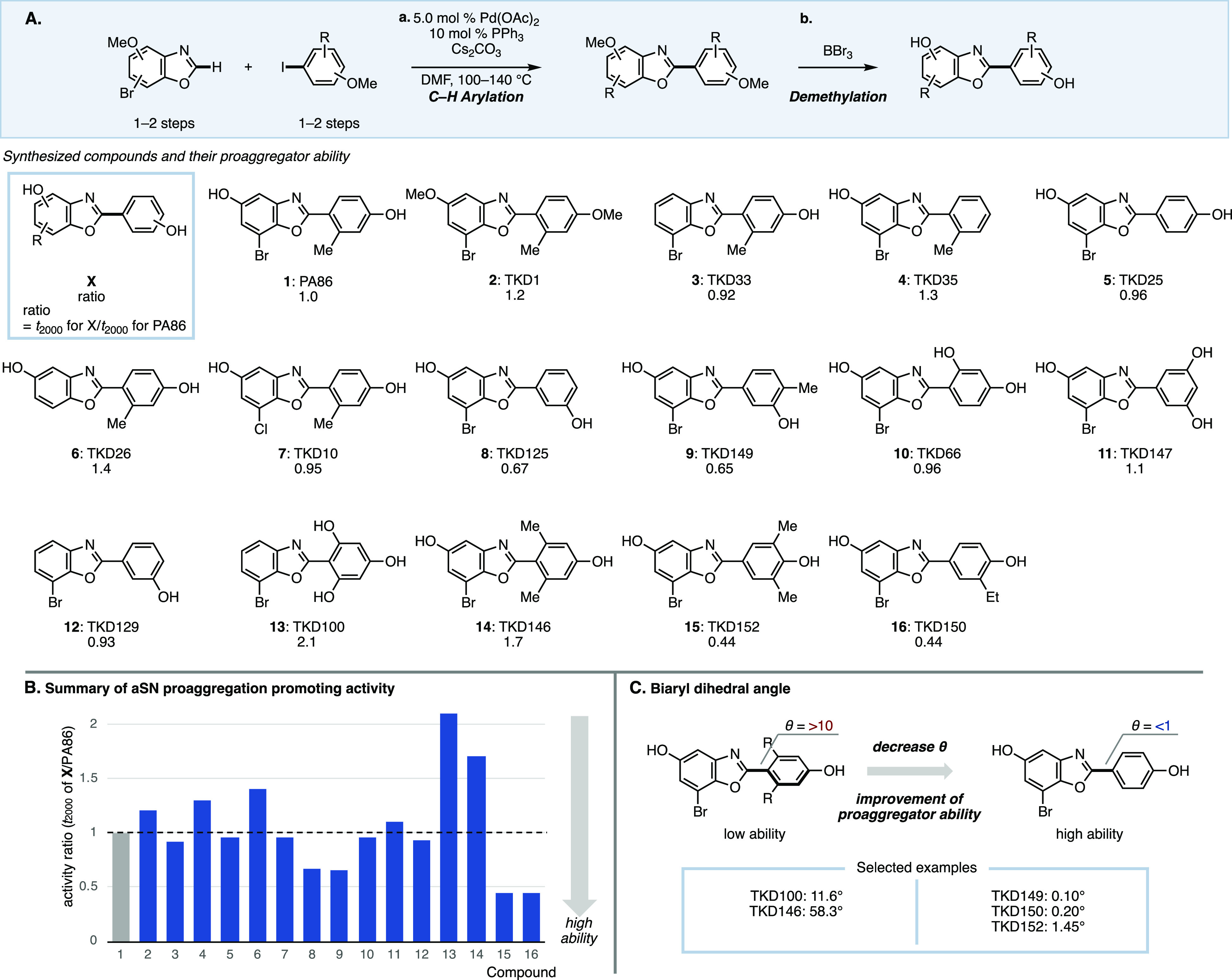
(A) General synthetic approach for the synthesis of PA86 (**1**) and its analogues (**2**–**16**) via C–H arylation. Reaction conditions: (a) benzoxazole (1.0 equiv), iodoarene (1.0 equiv), Pd(OAc)_2_ (5.0 mol %), PPh_3_ (10 mol %), Cs_2_CO_3_ (2.0 equiv), dimethylformamide, 100–140 °C, 26–73% yield. (b) BBr_3_ (2.3–14.2 equiv), CH_2_Cl_2_, −78 °C to RT. See SI for details. (B) Synthesized PA86 analogues and their aggregation promoting ability toward aSN expressed as a ratio (*t*_2000_ for compound/*t*_2000_ for PA86, see SI for details). (C) Possible correlation between the proaggregator ability and the biaryl dihedral angle.

The synthesized compounds **1**–**16** were then assessed for their ability to promote aggregation of the monomeric αSN protein by RT-QuIC assay. Specifically, compounds **1**–**16** were incubated in the presence of aSN preformed fibrils (PFF), thioflavin T, and a soluble aSN monomer and agitated for 60 h. Three control runs in the absence of compound were performed in parallel for every experiment, which indicated that presence of dimethyl sulfoxide (DMSO) had only a marginal effect on PFF-dependent aggregation in comparison to the control without DMSO (see the Supporting Information for details). The compound’s aggregation promoting activity was evaluated by measuring the *t*_2000_ value, the time required to reach fluorescence intensity of 2000 arbitrary unit (au), and then expressing it as a ratio by comparing with *t*_2000_ value of PA86 (Figure 2A,B). A low activity ratio is desirable since a lower activity ratio means a greater aggregation ability. We first set out to investigate the crucial functional groups present in PA86 that are required to promote aggregation of the monomeric aSN protein. Replacement of both hydroxy groups with methoxy groups reduced the aggregation activity (TKD1). Removing either hydroxy group had opposing effects as TKD33 showed slightly higher activity than the parent compound PA86, but TKD35 had lower activity. The result of TKD25 showed that the methyl group had negligible effect on the aggregation ability, whereas removal of bromide decreased the aggregation activity as seen in TKD26. In contrast, substituting the bromide to chloride (TKD10) showed similar aggregation ability as PA86.

We then focused our attention to alter the substitution pattern and number of hydroxy groups present on the phenyl ring. Changing the position of the hydroxy group from the *para*- (TKD25) to the *meta*-position (TKD125) improved the aggregation ability. Introduction of an additional methyl substituent (TKD149) did not show any improvement in activity. The effect of the dihydroxy phenyl group was negligible, where a similar aggregation activity to PA86 was observed for TKD66 and TKD147. With only one hydroxy group present on the right aryl group, TKD129 showed similar aggregation activity as PA86. TKD100, which has a trihydroxy phenyl group, resulted in significant reduction of aggregation ability. A similar compound bearing a trisubstituted phenyl moiety (TKD146) showed weaker aggregation ability than PA86. Surprisingly, an unexpected improvement of aggregation ability was realized when the two methyl substituents were placed at the *meta*-position (TKD152). Finally, TKD150 bearing a mono substituted alkyl phenol showed comparable aggregation ability with TKD152.

Although an obvious structure–activity relationship of PA86 analogues to the aggregation-promoting ability was not evident, the stark contrast between TKD146 and TKD152/TKD150 suggested to us that the absence of an *ortho* substituent was crucial. It has been reported that thioflavin T binds to β-sheet motifs and amyloids by achieving a planar conformation to give an intense fluorescence signal that is detected during the RT-QuIC assay.^22,23^ We reasoned that the achievement of a planar conformation by the molecule may play a role to interact with aSN monomers or fibrils to promote its aggregation. To investigate on the planarity of the compound, the dihedral angle of minimized energy structures of selected compounds was determined (Figure 2C).^24^ Compounds on the left side showing poor aggregation ability possessed larger dihedral angles, whereas the compounds on the right side showing good aggregation ability had dihedral angles of nearly 0. Based on the above rationale, we reasoned that TKD152 and TKD150 are able to promote aSN better than PA86 due to the planarity of the molecule.

At this point, we addressed subtle aspects of the RT-QuIC assay to confirm that the compounds are exhibiting proaggregator properties. First, we confirmed that the compounds did not influence the fluorescence signal from thioflavin T at various concentrations of PFF (Figure 3A). While marginal effect to the fluorescence signal was observed for TKD149 at low concentration of PFF and for TKD150 at higher concentrations of PFF, the fluorescence signal from thiovlavin T remains unaffected. Thus, the observed increase of fluorescence signal in the RT-QuIC assay results from thioflavin T binding to the formed fibrils and not due to the presence of compounds. Next, we checked whether the compound is promoting aggregation by forming colloidal aggregators.^25^ All 16 compounds were checked using online database, indicating that the compounds are not similar to previously known colloidal aggregators (see Supporting Information for details).^26^ Furthermore, TKD150 was experimentally verified to be void of colloids by conducting a particle size analysis of a sample prepared in the same buffer solution used in the RT-QuIC assay (Figure 3B).

**Figure 3:**
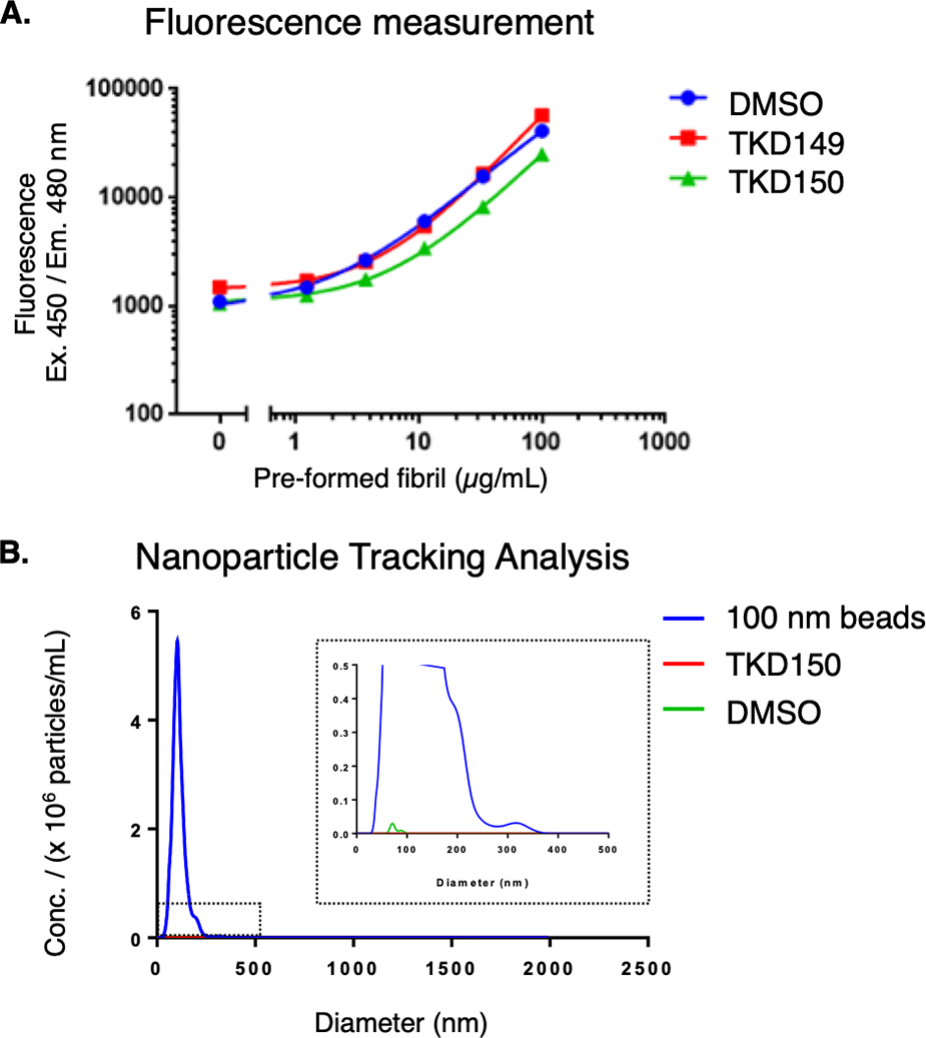
(A) Measurement of thioflavin T fluorescence signal. (B) Particle size analysis of TKD150.

Having identified several compounds that promote aggregation of aSN monomers better than PA86, we investigated whether these compounds selectively bind to aSN monomers or fibrils. To this end, affinity selection mass spectroscopy (ASMS)^27−29^ between the proaggregator compound and aSN monomer or fibril was performed. Up to 100 μM proaggregator compound was added to a solution of soluble aSN monomer, for which none of the compounds showed binding. Next, the compound’s affinity toward aSN fibril was examined, which indicated binding of TKD149 and TKD152 toward aSN fibril and determined the binding constant (*K*_d_) as 18.4 and 18.67 μM, respectively (Figure 4).^30^ However, we were not able to detect binding for other compounds, including TKD125 and TKD150 that exhibited good proaggregating ability. We speculate that these compounds have either weak binding toward aSN fibril or fast *k*_off_, resulting with no signal. Overall, the ASMS data revealed selective binding of TKD149 and TKD152 to aSN fibril while binding of compounds to aSN monomers could not be detected. These data suggest that the proaggregator compounds bind to aSN fibrils and consequently accelerates the aggregation process of aSN monomers.

**Figure 4:**
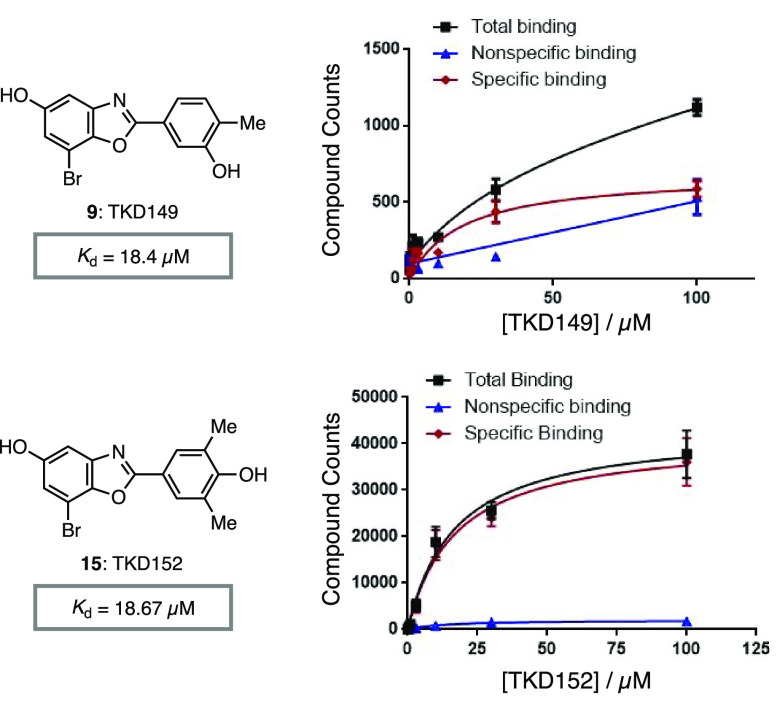
Determination of binding constant for TKD149 toward aSN fibril from ASMS studies.

Lastly, we confirmed that the addition of proaggregator compounds resulted in aggregation of soluble aSN monomers into aSN fibrils by obtaining transmission electron microscopy (TEM) images. TKD149 and TKD150 were selected based on their varying ability to promote aggregation of the aSN monomer. A sample of the soluble aSN monomer and proaggregator compound was shaken in the absence of thioflavin T for 60 h, and then TEM images of the resulting aggregates were obtained (Figure 5). As expected, formation aSN fibril was confirmed in all of the samples with proaggregator compound (TKD149 and TKD150). The control run that only contained DMSO and the aSN monomer had also formed aSN fibril, due to the self-aggregation property of aSN monomers under shaking conditions.^31^ Taken together with the RT-QuIC assay results, the obtained TEM images corroborates the role of benzoxazole compounds as proaggregator for aSN monomer into aSN fibril.

**Figure 5:**
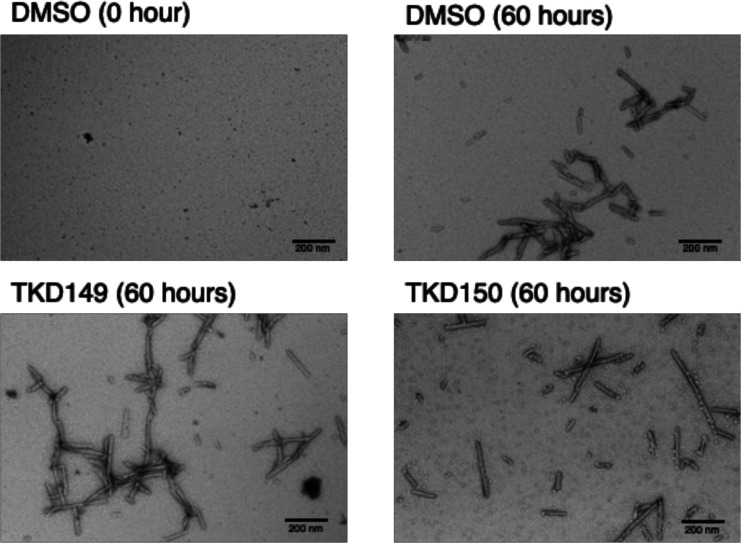
TEM images of aSN protein agitated with proaggregator in the absence of thioflavin T.

In summary, we have discovered TKD152 and TKD150, which can promote the aggregation of monomeric aSN into fibril twice as fast as a previously known proaggregator, PA86. The identification of these two compounds was made possible by employing a Pd-catalyzed C–H arylation reaction, allowing rapid preparation of PA86 analogues. A crucial requirement for the compound to promote aggregation has been identified as the absence of an *ortho* substituent, presumably to achieve a planar conformation that is necessary to interact with aSN fibril. Lastly, TEM images of aSN monomer treated with proaggregator compounds were obtained to confirm the formation of aSN fibril. Further studies using patient-derived cerebrospinal fluid is in progress to ascertain the compound’s value in a clinical setting. Results from these investigations will be reported in due course.

## Supporting Information Available

Synthetic experimental details, characterization data, and biological assay protocols (PDF)

## Abbreviations

PD: Parkinson’s disease
DLB: dementia with Lewy bodies
LB: Lewy bodies
aSN: α-synuclein
RT-QuIC: real time quaking induced conversion
ASMS: affinity selection mass spectroscopy
TEM: transmission electron microscopy
