# Supplementary material for: Identification of α-Synuclein Proaggregator: Rapid Synthesis and Streamlining RT-QuIC Assays in Parkinson’s Disease
Source: ACS Med Chem Lett. 2022 Aug 11;13(9):1421–6. doi: 10.1021/acsmedchemlett.2c00138 (PMC9465709; doi:10.1021/acsmedchemlett.2c00138)
Supplement: Supplementary file 1 — ml2c00138_si_001.pdf [file ml2c00138_si_001.pdf]

Supporting Information

Identification of α-Synuclein Proaggregator: Rapid Synthesis and Streamlining RT-QuIC Assays in Parkinson's Disease

Fumito Takada,[a] Takahito Kasahara,[b] Kentaro Otake,[b] Takamitsu Maru,[c] Masanori Miwa,[c] Kei Muto,[d] Minoru Sasaki,[b] Yoshihiko Hirozane,[b] Masato Yoshikawa,*[b] and Junichiro Yamaguchi*[a]

[a] *Department of Applied Chemistry, Waseda University, 513 Wasedatsurumakicho, Shinjuku, Tokyo 162-0041, Japan*

[b] *Takeda Pharmaceutical Company Limited, 2-26-1 Muraoka-Higashi, Fujisawa, Kanagawa 251-8555, Japan*

[c] *Axcelead Drug Discovery Partners Inc., 2-26-1 Muraoka-Higashi, Fujisawa, Kanagawa 251-8555, Japan*

[d] *Waseda Institute for Advanced Study, Waseda University, 513 Wasedatsurumakicho, Shinjuku, Tokyo 162-0041, Japan*

E-mail: junyamaguchi@waseda.jp (JY), masato.yoshikawa@takeda.com (MY)

Table of Contents

1. General S2
2. Preparation of benzoxazoles **S3** and iodoarenes **S4** S3–S5
3. Synthesis of PA86 analogues via Pd-catalyzed C–H arylation S6–S15
4. RT-QuIC assay S16–S17
5. Binding assay with Affinity Selection-Mass Spectrometry (AS-MS) S18
6. Transmission electron microscopy (TEM) analysis S19
7. Particle size analysis S20
8. Aggregation Advisor results S21–23
9. References S24
10. ^{1}H and ^{13}C NMR Spectra S25–S60
11. HPLC Chromatogram S61–S66

1. General

Unless otherwise noted, all reactants or reagents including dry solvents were obtained from commercial suppliers and used as received. 1-Iodo-2-methylbenzene (**S4b**) and 1-iodo-4-methoxybenzene (**S4c**) were obtained from Tokyo Chemical Industry Corporation (TCI). BBr_3 was obtained from Kishida Chemical. $Pd(OAc)_2$ was obtained from FUJIFILM Wako Pure Chemical Corporation. PPh_3 and Cs_2CO_3 were obtained from Kanto Chemical. Iodo-4-methoxy-2-methylbenzene (**S4a**),[1] 1-iodo-3-methoxybenzene (**S4d**),[2] 4-iodo-2-methoxy-1-methylbenzene (**S4e**),[3] 1-iodo-2,4-dimethoxybenzene (**S4f**),[1] 1-iodo-3,5-dimethoxybenzene (**S4g**),[4] 2-iodo-5-methoxy-1,3-dimethylbenzene (**S4i**),[5] and 5-iodo-2-methoxy-1,3-dimethylbenzene (**S4j**),[6] were synthesized according to procedures and the spectra matched with those of compounds reported in the literature. Unless otherwise noted, all reactions were performed with dry solvents under an atmosphere of N_2 in dried glassware using standard vacuum-line techniques. All C–H arylation reactions of benzoxazoles with iodoarenes were performed in 8-mL glass vessel tubes equipped with a screw cap and heated (IKA Plate RCT Digital) in a 16-well aluminum reaction block (IKA DB4.3 Block) unless otherwise noted. All work-up and purification procedures were carried out with reagent-grade solvents in air.

Analytical thin-layer chromatography (TLC) was performed using Silica-gel 70 TLC Plate-Wako (0.25 mm). The developed chromatogram was analyzed by UV lamp (254 nm). Flash column chromatography was performed with Biotage Isolera® equipped with Biotage Sfär Cartridge Silica D column. Preparative thin-layer chromatography (PTLC) was performed using Wakogel B5-F silica coated plates (0.75 mm) prepared in our laboratory. High-resolution mass spectra (HRMS) were conducted on Thermo Fisher Scientific ExactivePlus (ESI). Nuclear magnetic resonance (NMR) spectra were recorded on a JEOL JNM-ECS-400 (^{1}H 400 MHz, ^{13}C 101 MHz), JEOL JNM-ECZ-400 (^{1}H 400 MHz, ^{13}C 101 MHz,), and JEOL JNM-ECZ-600R (^{1}H 600 MHz, ^{13}C 151 MHz). Chemical shifts for ^{1}H NMR are expressed in parts per million (ppm) relative to tetramethylsilane (δ 0.00 ppm) in $CDCl_3$ and CHD_2COCD_3 (δ 2.05 ppm) in acetone-d_6. Chemical shifts for ^{13}C NMR are expressed in ppm relative to $CDCl_3$ (δ 77.0 ppm) and acetone-d_6 (δ 29.8 ppm). Data are reported as follows: chemical shift, multiplicity (s = singlet, d = doublet, dd = doublet of doublets, t = triplet, td = triplet of doublets, m = multiplet, br = broad), coupling constant (Hz), and integration. Unless otherwise noted, the purity of all test compounds was >95% as determined by HPLC (Waters Acquity H Class) equipped with either an Acquity UPLC BEH C18 column (1.7 mm 2.1 × 30 mm column) or Capcell Pak C18AQ column (50 mm x 3.0 mm I.D., Shiseido, Japan) and detected with by Corona CAD (charged aerosol detector) or photo diode array detector. The HPLC analysis of samples were performed at 50 °C (oven temperature) with a flow rate of 0.5 mL/min. Under neutral conditions, mobile phase A and B consisted of mixture of 5 mmol/L ammonium acetate in water and 5 mmol/L ammonium acetate in acetonitrile, respectively. Under acidic conditions, mobile phase A and B were a mixture of 0.2% formic acid in water and 10 mmol/L ammonium formate and 0.2% formic acid in acetonitrile, respectively. The ratio of mobile phase B was increased linearly from 5% to 99% over 3.2 min, 99% over the next 0.4 min.

2. Preparation of benzoxazoles S3 and iodoarenes S4

Structure of benzoxazoles S3 and iodoarenes S4

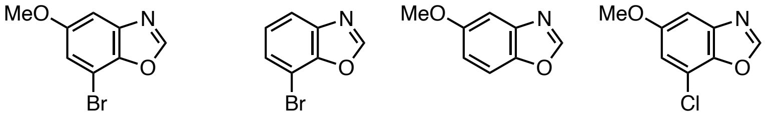

S3A **S3B** **S3C** **S3D**

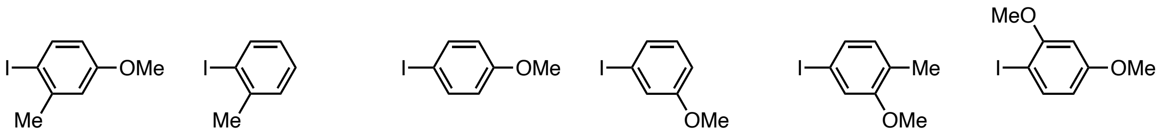

S4a **S4b** **S4c** **S4d** **S4e** **S4f**

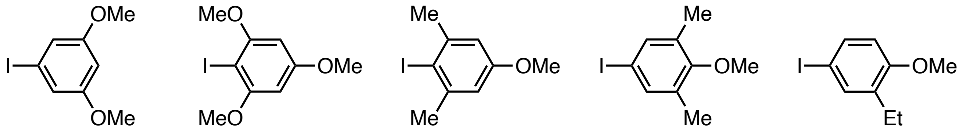

S4g **S4h** **S4i** **S4j** **S4k**

General procedure for the synthesis of benzoxazoles S3

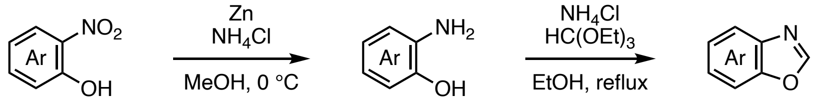

S1 **S2** **S3**

To a solution of nitrophenol (**S1**: 1.0 equiv) in MeOH (0.20 M) were added zinc dust (10 equiv) and then ammonium chloride (10 equiv) portionwise over 5 minutes at 0 °C. The reaction mixture was stirred at 0 °C for 10 min. The reaction mixture was filtered through a pad of Celite® with EtOAc as an eluent and concentrated *in vacuo*. The mixture was diluted with EtOAc and water, and then extracted three times with EtOAc. Combined organic layer were washed with brine, dried over Na_2SO_4, filtrated, and then concentrated to afford aminophenol **S2** as a black solid. This was used for next step without further purification.

To a solution of the aminophenol (**S2**: 1.0 equiv) in EtOH (0.42 M) were added ammonium chloride (0.20 equiv) and triethyl orthoformate (1.5 equiv). The reaction mixture was refluxed for three hours with monitoring the reaction progress on TLC. Upon the completion of reaction, the mixture was cooled to room temperature. The mixture was concentrated *in vacuo*. The residue was purified by Isolera® to afford benzoxazole **S3**.

7-bromo-5-methoxybenzo[*d*]oxazole (S3A)

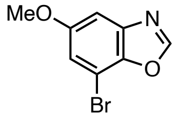

Purification by Isolera® (hexane/EtOAc = 9:1 to 4:1) to afford **S3A** as a violet solid (5.0 mmol scale, 980 mg, 86% yield). ^{1}H NMR (400 MHz, $CDCl_3$) δ 8.09 (s, 1H), 7.21 (d, J = 2.0 Hz, 1H), 7.17 (d,

J = 2.0 Hz, 1H), 3.86 (s, 3H); ^{13}C NMR (101 MHz, $CDCl_3$) δ 157.8, 153.1, 143.0, 141.0, 117.4, 102.7, 102.6, 56.1; HRMS (ESI) *m*/*z* calcd for $C_8H_7BrNO_2$ $[M+H]^+$: 227.9655 found 227.9653.

7-bromobenzo[*d*]oxazole (S3B)

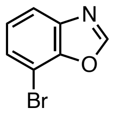

Purification by Isolera® (hexane/EtOA c= 9:1 to 4:1) to afford **S3B** as an orange solid (3.0 mmol scale, 362 mg, 61% yield). The spectra are matched with those of the compounds reported in the literature.[7]

5-methoxybenzo[*d*]oxazole (S3C)

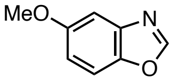

Purification by Isolera® (hexane/EtOAc = 9:1 to 4:1) to afford **S3C** as an orange solid (3.0 mmol scale, 240 mg, 54% yield). The spectra are matched with those of the compounds reported in the literature.[8]

7-chloro-5-methoxybenzo[*d*]oxazole (S3D)

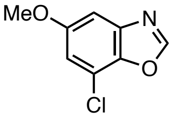

Purification by Isolera® (hexane/EtOAc = 9:1 to 4:1) to afford **S4D** as a white solid (1.5 mmol scale, 230 mg, 84% yield). ^{1}H NMR (400 MHz, $CDCl_3$) δ 8.09 (s, 1H), 7.17 (d, J = 2.0 Hz, 1H), 7.02 (d, J = 2.0 Hz, 1H), 3.86 (s, 3H); ^{13}C NMR (101 MHz, $CDCl_3$) δ 157.6, 153.3, 141.6, 141.4, 116.2, 114.7, 102.2, 56.1; HRMS (ESI) *m*/*z* calcd for $C_8H_7ClNO_2$ $[M+H]^+$: 184.0160 found 184.0161.

Synthesis of 2-ethyl-4-iodo-1-methoxybenzene (S4f) [9]

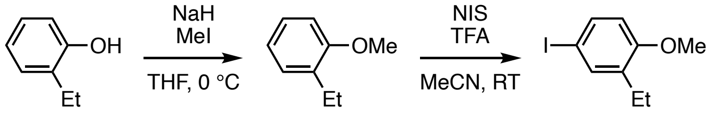

To a solution of 2-ethylphenol (180 μL, 1.5 mmol, 1.0 equiv) in THF (0.50 M) were added sodium hydride (60%, dispersion in paraffin liquid: 94.4 mg, 2.3 mmol, 1.5 equiv) at 0 °C. After stirring for 15 min, methyl iodide (140 μL, 2.3 mmol, 1.5 equiv) was added dropwise to the mixture at 0 °C. After stirring the solution for 2 h at room temperature, the reaction was quenched with sat. NH_4Cl aq. The mixture was extracted three times with Et_2O. The combined organic layer was dried over $MgSO_4$, filtrated, and concentrated *in vacuo* to afford 2-ethylanisol, which was used for next step without further

purification. To a solution of 2-ethylanisol in MeCN (0.20 M) was added NIS (373.3 mg, 1.7 mmol, 1.1 equiv) and TFA (240 μL, 3.1 mmol, 2.0 equiv). After completion of reaction, the reaction was quenched with sat. $Na_2S_2O_3$ aq. and 1 M NaOH aq. The mixture was extracted three times with Et_2O, dried over with $MgSO_4$, filtrated and concentrated *in vacuo*. The residue was purified by Isolera® (hexane/EtOAc = 99:1 to 19:1) to afford **S4f** as a colorless oil (131.4 mg, 33% yield). ^{1}H NMR (400 MHz, $CDCl_3$) δ 7.44 (dd, *J* = 8.2, 2.4 Hz, 1H), 7.42 (d, *J* = 2.4 Hz, 1H), 6.60 (d, *J* = 8.4 Hz, 1H), 3.79 (s, 3H), 2.57 (q, *J* = 7.6 Hz, 2H), 1.16 (t, *J* = 7.6 Hz, 3H); ^{13}C NMR (101 MHz, $CDCl_3$) δ 157.3, 137.4, 135.44, 135.38, 112.4, 82.8, 55.3, 22.9, 13.9; HRMS (DART) *m*/*z* calcd for $C_9H_{11}IO$ $[M]^+$: 261.9849 found 261.9847.

Synthesis of 2-iodo-1,3,5-trimethoxybenzene (S4h)

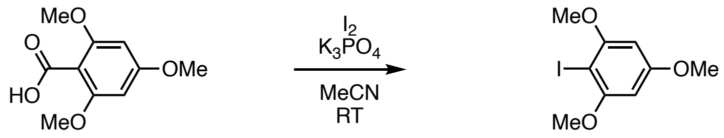

S4h

Following Larrosa's protocol,[1] **S4h** was prepared. To a dried round-bottom flask were added iodine (1.02 g, 4.0 mmol, 4.0 equiv), 2,4,6-trimethoxybenzoic acid (212 mg, 1.0 mmol, 1.0 equiv), and K_3PO_4 (212 mg, 1.0 mmol, 1.0 equiv). The flask was placed under vacuum (quickly) and refilled with N_2 gas three times, and then MeCN (5.0 mL) was added. Capping the flask with a rubber septum, the mixture was stirred at room temperature for 4 h. The mixture was added sat. $Na_2S_2O_8$ aq. and sat. $NaHCO_3$ aq. The mixture was extracted three times with CH_2Cl_2, dried over with Na_2SO_4, filtrated, and then concentrated *in vacuo* to afford **S4h** (265 mg, 90% yield) as a white solid. The spectra are matched with those of the compounds reported in the literature.[10]

3. Synthesis of PA86 analogues via Pd-catalyzed C–H arylation

General procedure A: Pd-catalyzed C–H arylation

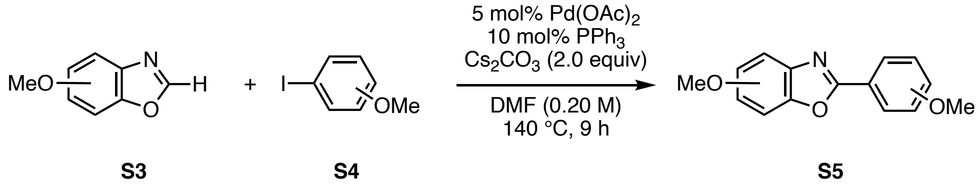

Following Miura's procedure with slightly modifications,[11] the C–H arylation of benzoxazoles with iodoarenes was conducted. An 8-mL glass vessel containing a magnetic stirring bar and Cs_2CO_3 (130 mg, 0.40 mmol, 2.0 equiv) was dried with a heatgun *in vacuo* and filled with N_2 gas after cooling to room temperature. To this vessel were added oxazole **S3** (0.20 mmol), $Pd(OAc)_2$ (2.3 mg, 0.010 mmol, 5 mol%), PPh_3 (5.3 mg, 0.020 mmol, 10 mol%) and iodoarene **S4** (1.0–1.4 equiv). The vessel was placed under vacuum and refilled with N_2 gas three times, and then added DMF (1.0 mL). The vessel was sealed and then heated at 140 °C for 9 h in an oil bath with stirring. After cooling the reaction mixture to room temperature, the mixture was passed through a short silica-gel pad with EtOAc as an eluent and concentrated *in vacuo*. The residue was purified by PTLC to afford **S5**.

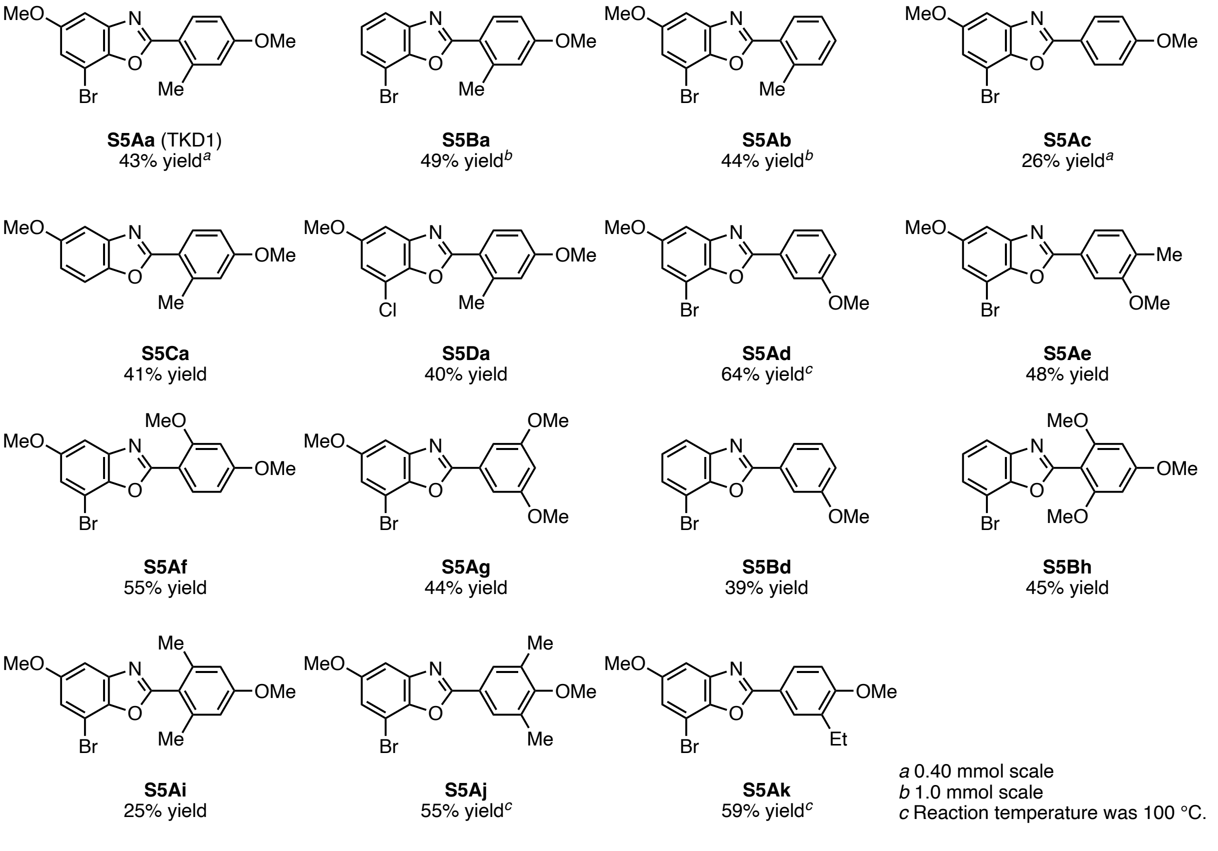

Table S1. Summary of the yields of the C–H arylation products.

General procedure B: demethylation using BBr_3

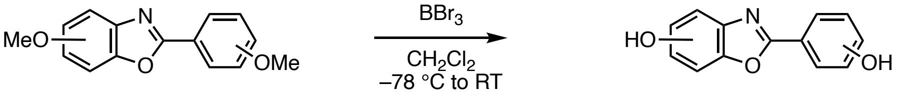

S5 **1–16**

A solution of 2-arylbenzoxazoles **S5** in CH_2Cl_2 (0.10 M) was cooled to –78 °C. To this solution was added boron tribromide (2.3–16.1 equiv) dropwise at –78 °C. The reaction mixture was gradually allowed to warm to room temperature. After stirring for 24 h at room temperature, the mixture was diluted with EtOAc. The mixture was added MeOH and then sat. $NaHCO_3$ aq. The mixture was extracted three times with EtOAc, dried over Na_2SO_4, filtered, and then concentrated *in vacuo*. The residue was purified by Isolera® to afford 2-aryloxazoles **1–16**.

Synthesis of 7-bromo-2-(4-hydroxy-2-methylphenyl)benzo[*d*]oxazol-5-ol (1: PA86)

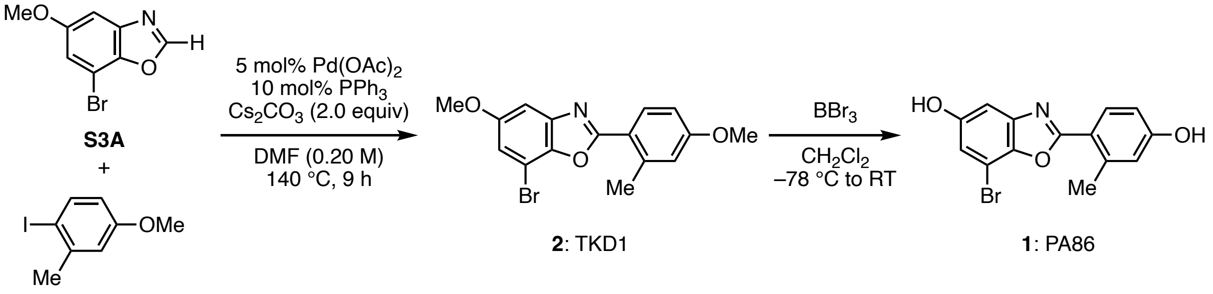

Following the General procedure A, **S3A** (91.2 mg, 0.40 mmol, 1.0 equiv) and **S4a** (143.5 mg, 0.56 mmol, 1.4 equiv) were reacted. Purification by PTLC (hexane/EtOAc = 4:1) afforded 7-bromo-5-methoxy-2-(4-methoxy-2-methylphenyl)benzo[*d*]oxazole (**2**: TKD1) as a white solid (59.5 mg, 43% yield).

Following the General procedure B, **2** (60 mg, 0.14 mmol) and BBr_3 (100 µL, 1.0 mmol, 6.1 equiv) were reacted. Purification by Isolera® (hexane/EtOAc = 9:1 to 1:1) to afford **1** (PA86) as a white solid (42.4 mg, 77% yield).

7-bromo-5-methoxy-2-(4-methoxy-2-methylphenyl)benzo[*d*]oxazole (TKD1)

^{1}H NMR (400 MHz, $CDCl_3$) δ 8.17 (d, J = 8.4 Hz, 1H), 7.19 (d, J = 2.0 Hz, 1H), 7.08 (d, J = 2.0 Hz, 1H), 6.89–6.86 (m, 2H), 3.88 (s, 3H), 3.86 (s, 3H), 2.80 (s, 3H); ^{13}C NMR (101 MHz, $CDCl_3$) δ 164.4, 161.7, 157.6, 143.2, 141.2, 131.8, 118.3, 117.0, 115.8, 111.6, 102.4, 101.9, 56.1, 55.3, 22.6 (one peak is missing due to overlapping.) HRMS (ESI) m/z calcd for $C_{16}H_{15}BrNO_3$ $[M+H]^+$: 348.0230 found 348.0230. The purity of this compound was determined as 90.6% by HPLC analysis.

7-bromo-2-(4-hydroxy-2-methylphenyl)benzo[*d*]oxazol-5-ol (PA86)

^{1}H NMR (400 MHz, acetone-d_6) δ 8.92 (br, 1H), 8.07 (d, J = 9.2 Hz, 1H), 7.12 (d, J = 2.4 Hz, 1H), 7.06 (d, J = 2.4 Hz, 1H), 6.90–6.89 (m, 2H), 2.74 (s, 3H); ^{13}C NMR (101 MHz, acetone-d_6) δ 165.1, 161.1, 156.3, 144.5, 143.1, 142.1, 132.5, 119.3, 117.8, 116.5, 114.3, 105.3, 101.8, 22.6; HRMS (ESI) m/z calcd for $C_{14}H_{11}BrNO_3$ $[M+H]^+$: 319.9917 found 319.9916.

Synthesis of 4-(7-bromobenzo[*d*]oxazol-2-yl)-3-methylphenol (3: TKD33)

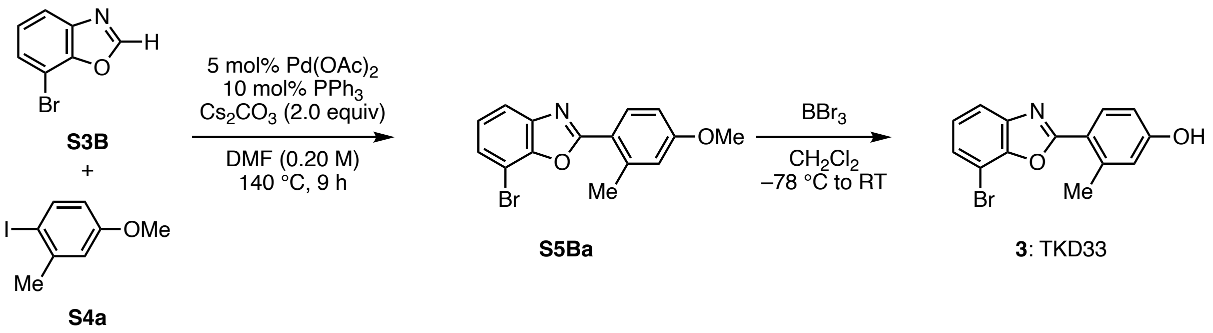

Following the General procedure A, **S3B** (198 mg, 1.0 mmol, 1.0 equiv) and **S4a** (213 µL, 1.4 mmol, 1.4 equiv) were reacted. Purification by Isolera® (hexane/EtOAc = 100:1 to 4:1) afforded 7-bromo-2-(4-methoxy-2-methylphenyl)benzo[*d*]oxazole (**S5Ba**) as a white solid (154.4 mg, 49% yield).

Following the General procedure B, **S5Ba** (27.6 mg, 94 µmol, 1.0 equiv) and BBr_3 (20 µL, 0.21 mmol, 2.4 equiv) were reacted. Purification by Isolera® (hexane/EtOAc = 9:1 to 2:1) to afford **3** (TKD33) as a white solid (21.6 mg, 82% yield). ^{1}H NMR (400 MHz, acetone-d_6) δ 9.09 (br, 1H), 8.16–8.09 (m, 1H), 7.71 (d, J = 8.0 Hz, 1H), 7.55 (d, J = 8.0 Hz, 1H), 7.32 (t, J = 8.0 Hz, 1H), 6.94–6.89 (m, 2H), 2.76 (s, 3H); ^{13}C NMR (101 MHz, acetone-d_6) δ 164.4, 161.2, 148.9, 144.0, 142.3, 132.7, 128.3, 126.6, 119.6, 119.4, 117.5, 114.3, 102.4, 22.6; HRMS (ESI) *m*/*z* calcd for $C_{14}H_{11}BrNO_2$ $[M+H]^+$: 303.9968 found 303.9968.

Synthesis of 7-bromo-2-(*o*-tolyl)benzo[*d*]oxazol-5-ol (4: TKD35)

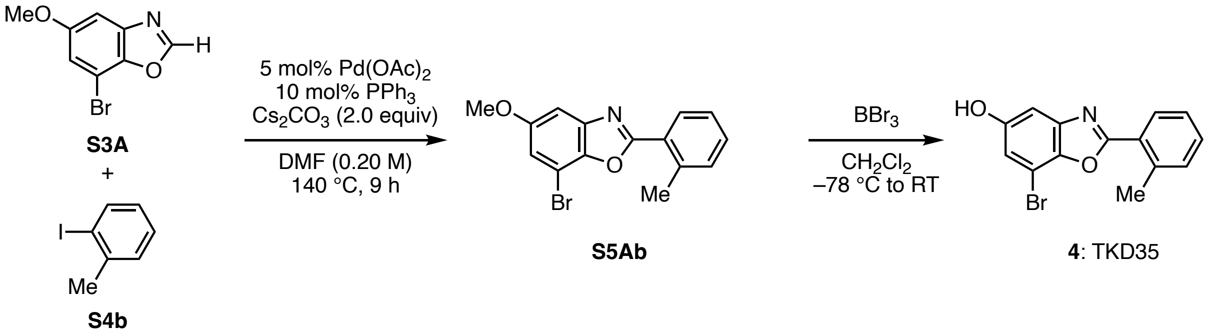

Following the General procedure A, **S3A** (228 mg, 1.0 mmol, 1.0 equiv) and **S4b** (178.0 µL, 1.4 mmol, 1.4 equiv) were reacted. Purification by PTLC (hexane/EtOAc = 4:1) afforded 7-bromo-5-methoxy-2-(*o*-tolyl)benzo[*d*]oxazole (**S5Ab**) as a white solid (141.5 mg, 44% yield).

Following the General procedure B, **S5Ab** (30 mg, 94 µmol) and BBr_3 (20 µL, 0.21 mmol, 2.2 equiv) were reacted. Purification by Isolera® (hexane/EtOAc = 9:1 to 1:1) to afford **4** (TKD35) as a white solid (15.6 mg, 54% yield). ^{1}H NMR (600 MHz, acetone-d_6) δ 8.87 (s, 1H), 8.18 (d, J = 7.6 Hz, 1H), 7.52–7.49 (m, 1H), 7.46–7.41 (m, 2H), 7.17 (d, J = 1.8 Hz, 1H), 7.13 (d, J = 1.8 Hz, 1H), 2.79 (s, 3H); ^{13}C NMR (151 MHz, acetone-d_6) δ 164.7, 156.5, 144.3, 143.5, 139.8, 132.8, 132.3, 130.6, 127.2,

126.5, 117.4, 105.6, 102.2, 22.4; HRMS (ESI) *m/z* calcd for $C_{14}H_{11}BrNO_2$ $[M+H]^+$: 303.9968 found 303.9967.

Synthesis of 7-bromo-2-(4-hydroxyphenyl)benzo[*d*]oxazol-5-ol (5: TKD25)

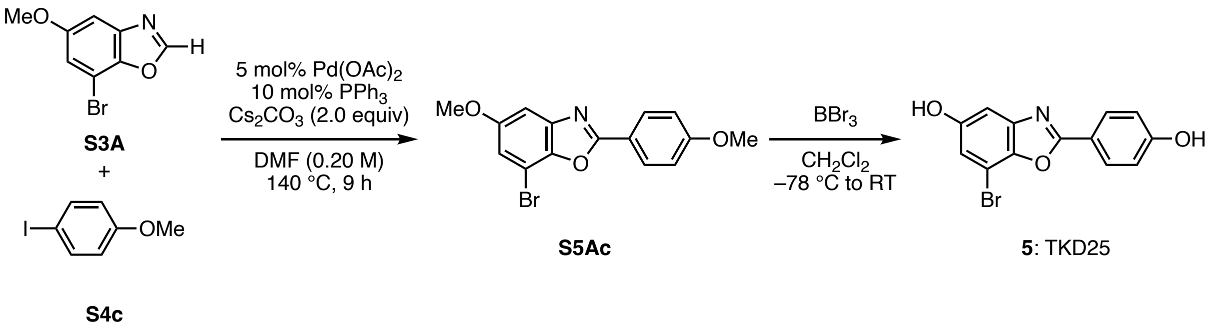

Following the General procedure A, **S3A** (91.1 mg, 0.40 mmol, 1.0 equiv) and **S4c** (186 mg, 0.80 mmol, 2.0 equiv) were reacted. Purification by PTLC (hexane/EtOAc = 9:1) afforded 7-bromo-5-methoxy-2-(4-methoxyphenyl)benzo[*d*]oxazole (**S5Ac**) as a white solid (35.4 mg, 26% yield).

Following the General procedure B, **S5Ac** (16.5 mg, 49 μmol) and BBr_3 (30 μL, 0.32 mmol, 6.5 equiv) were reacted. Purification by Isolera® (hexane/EtOAc = 9:1 to 1:1) to afford **5** (TKD25) as a white solid (11.6 mg, 77% yield). ^{1}H NMR (400 MHz, acetone-d_6) δ 9.00 (bs, 2H), 8.10 (d, J = 8.8 Hz, 2H), 7.08–7.03 (m, 4H); ^{13}C NMR (151 MHz, acetone-d_6) δ 164.9, 161.9, 156.5, 144.7, 143.8, 130.4, 118.9, 116.9, 116.5, 105.3, 102.0; HRMS (ESI) m/z calcd for $C_{13}H_9BrNO_3$ $[M+H]^+$: 305.9760 found 305.9759.

Synthesis of 2-(4-hydroxy-2-methylphenyl)benzo[*d*]oxazol-5-ol (6: TKD26)

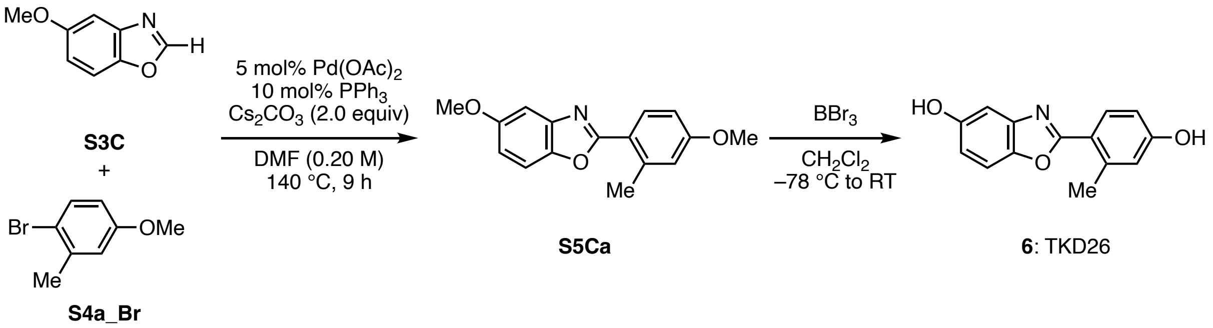

Following the General procedure A, **S3C** (29.8 mg, 0.20 mmol, 1.0 equiv) and **S4a_Br** (52 μL, 0.40 mmol, 2.0 equiv) were reacted. Purification by PTLC (hexane/EtOAc = 4:1) afforded 5-methoxy-2-(4-methoxy-2-methylphenyl)benzo[*d*]oxazole (**S5Ca**) as a white solid (22.3 mg, 41% yield).

Following the General procedure B, **S5Ca** (20.2 mg, 88 μmol) and BBr_3 (41 μL, 0.44 mmol, 5.1 equiv) were reacted. Purification by Isolera® (hexane/EtOAc = 9:1 to 1:2) to afford **6** (TKD26) as a white solid (17.8 mg, 98% yield). ^{1}H NMR (600 MHz, acetone-d_6) δ 8.97 (br, 1H), 8.42 (br, 1H), 8.03 (d, J = 9.0 Hz, 1H), 7.45 (d, J = 8.4 Hz, 1H), 7.13 (d, J = 2.4 Hz, 1H), 6.89–6.85 (m, 3H), 2.73 (s, 3H);

^{13}C NMR (151 MHz, acetone-d_6) δ 164.9, 160.6, 155.6, 144.9, 144.2, 141.8, 132.3, 119.3, 118.6, 114.2, 113.8, 111.0, 105.7, 22.7; HRMS (ESI) *m/z* calcd for $C_{14}H_{12}NO_3$ $[M+H]^+$: 242.0812 found 242.0810.

Synthesis of 7-chloro-2-(4-hydroxy-3-methylphenyl)benzo[*d*]oxazol-5-ol (7: TKD10)

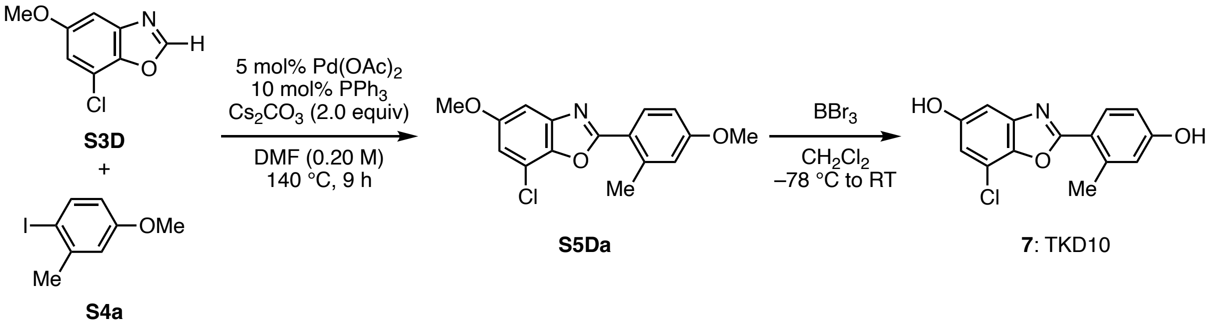

Following the General procedure A, **S3D** (36.7 mg, 0.20 mmol, 1.0 equiv) and **S4a** (52 µL, 0.34 mmol, 1.7 equiv) were reacted. Purification by PTLC (hexane/EtOAc = 6:1, then hexane/$CHCl_3$ = 1:15) afforded 7-chloro-5-methoxy-2-(4-methoxy-2-methylphenyl)benzo[*d*]oxazole (**S5Da**) as a white solid (24.6 mg, 40% yield).

Following the General procedure B, **S5Da** (20.2 mg, 66.5 µmol, 1.0 equiv) and BBr_3 (38 µL, 0.40 mmol, 6.0 equiv) were reacted. Purification by Isolera® (hexane/EtOAc = 9:1 to 1:3) to afford **7** (TKD10) as a white solid (18.3 mg, quant.). ^{1}H NMR (400 MHz, acetone-d_6) δ 8.91 (bs, 2H), 8.06 (d, *J* = 9.2 Hz, 1H), 7.08 (d, *J* = 2.4 Hz, 1H), 6.92 (d, *J* = 2.4 Hz, 1H), 6.91–6.86 (m, 2H), 2.73 (s, 3H); ^{13}C NMR (101 MHz, acetone-d_6) δ 165.3, 161.1, 156.1, 145.1, 142.2, 141.5, 132.6, 119.4, 117.9, 115.3, 114.3, 113.8, 104.9, 22.6; HRMS (ESI) *m/z* calcd for $C_{14}H_{11}ClNO_3$ $[M+H]^+$: 276.0422 found 276.0422.

Synthesis of 7-bromo-2-(3-hydroxyphenyl)benzo[*d*]oxazol-5-ol (8: TKD125)

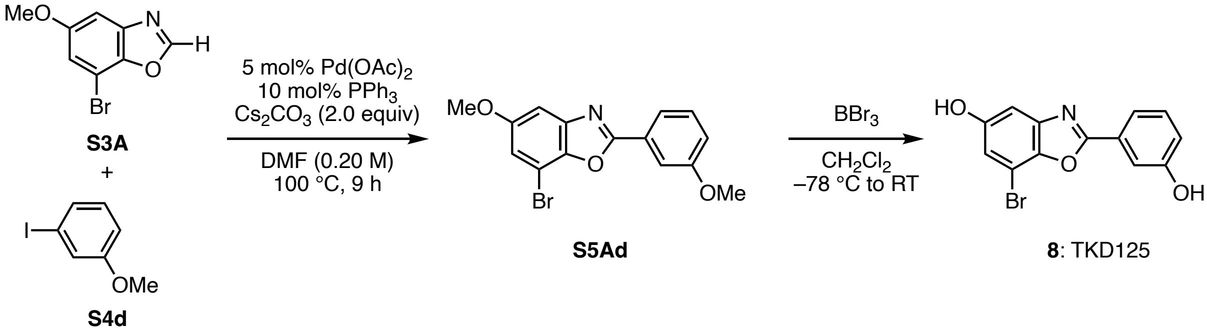

Following the General procedure A, **S3A** (45.6 mg, 0.20 mmol, 1.0 equiv) and **S4d** (46.8 mg, 0.20 mmol, 1.0 equiv) were reacted at 100 °C. Purification by PTLC (hexane/$CHCl_3$ = 3:7) afforded 7-bromo-5-methoxy-2-(3-methoxyphenyl)benzo[*d*]oxazole (**S5Ad**) as a white solid (43.1 mg, 64% yield).

Following the General procedure B, **S5Ad** (18.5 mg, 55 µmol) and BBr_3 (40 µL, 0.42 mmol, 7.6 equiv) were reacted. Purification by Isolera® (hexane/EtOAc = 9:1 to 1:2) to afford **8** (TKD125) as a

white solid (13.7 mg, 81% yield). ^{1}H NMR (400 MHz, acetone-d_6) δ 8.86 (s, 2H), 7.74–7.72 (m, 2H), 7.45 (td, J = 8.0, 2.0 Hz, 1H), 7.15–7.09 (m, 3H); ^{13}C NMR (101 MHz, acetone-d_6) δ 164.4, 158.8, 156.6, 144.4, 143.9, 131.3, 128.8, 120.1, 119.7, 117.4, 114.8, 105.6, 102.2; HRMS (ESI) *m*/*z* calcd for $C_{13}H_9BrNO_3$ [M+H]$^+$: 305.9760 found: 305.9758.

Synthesis of 7-bromo-2-(3-hydroxy-4-methylphenyl)benzo[*d*]oxazol-5-ol (9: TKD149)

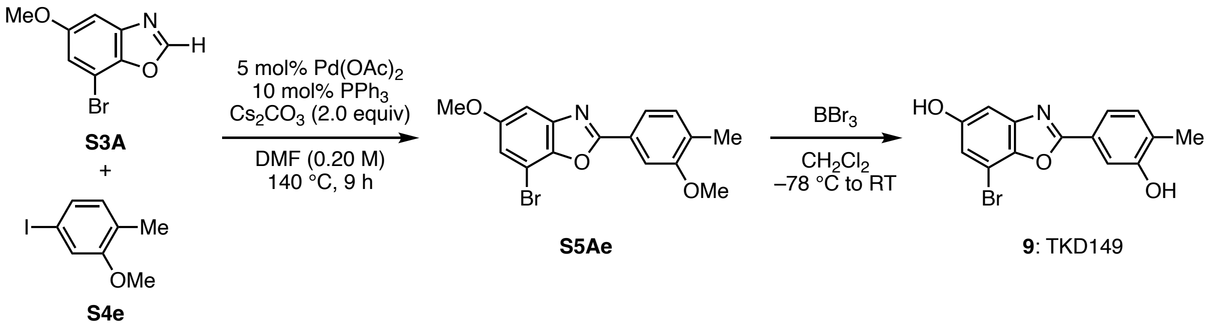

Following the General procedure A, **S3A** (45.6 mg, 0.20 mmol, 1.0 equiv) and **S4e** (49.6 mg, 0.20 mmol, 1.0 equiv) were reacted. Purification by PTLC (hexane/$CHCl_3$ = 1:2) afforded 7-bromo-5-methoxy-2-(3-methoxy-4-methylphenyl)benzo[*d*]oxazole (**S5Ae**) as a white solid (33.7 mg, 48% yield).

Following the General procedure B, **S5Ae** (20.4 mg, 59 µmol) and BBr_3 (70 µL, 0.74 mmol, 12.6 equiv) were reacted. Purification by Isolera® (hexane/EtOAc = 9:1 to 1:2) to afford **9** (TKD149) as a white solid (18.8 mg, quant.). ^{1}H NMR (400 MHz, acetone-d_6) δ 8.84 (s, 1H), 8.75 (s, 1H), 7.71 (s, 1H), 7.65 (d, J = 8.0 Hz, 1H), 7.33 (d, J = 8.0 Hz, 1H), 7.12–7.07 (m, 2H), 2.30 (s, 3H); ^{13}C NMR (101 MHz, acetone-d_6) δ 164.6, 156.7, 156.5, 144.4, 143.8, 132.4, 130.3, 126.2, 119.8, 117.1, 114.0, 105.5, 102.1, 16.4; HRMS (ESI) *m*/*z* calcd for $C_{14}H_{11}BrNO_3$ [M+H]$^+$: 319.9917 found 319.9914. The purity of this compound was determined as 93.5% by HPLC analysis.

Synthesis of 4-(7-bromo-5-hydroxybenzo[*d*]oxazol-2-yl)benzene-1,3-diol (10: TKD66)

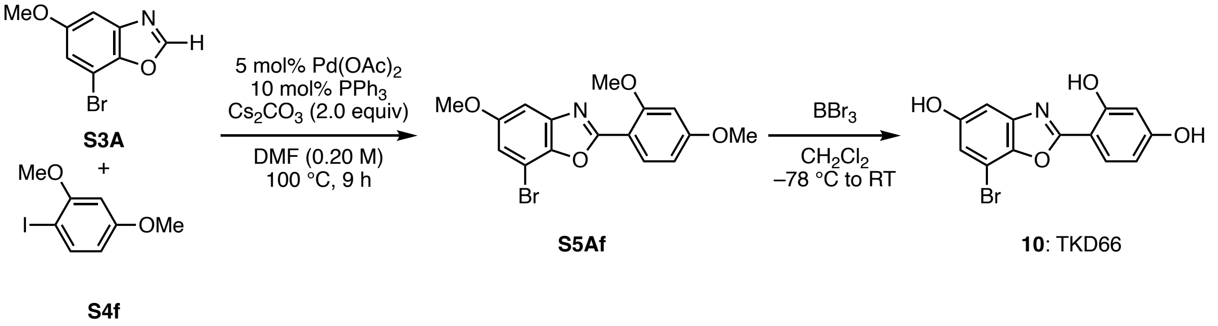

Following the General procedure A, **S3A** (45.6 mg, 0.20 mmol, 1.0 equiv) and **S4f** (52.8 mg, 0.20 mmol, 1.0 equiv) were reacted at 100 °C. Purification by PTLC (hexane/$CHCl_3$ = 1:4) afforded 7-bromo-2-(2,4-dimethoxyphenyl)-5-methoxybenzo[*d*]oxazole (**S5Af**) as a white solid (40.0 mg, 55% yield).

Following the General procedure B, **S5Af** (26.4 mg, 72 µmol) and BBr_3 (90 µL, 0.95 mmol, 13.1 equiv) were reacted. Purification by Isolera® (hexane/EtOAc = 9:1 to 2:5) to afford **10** (TKD66) as a

white solid (18.1 mg, 78%). ^{1}H NMR (400 MHz, acetone-d_6) δ 11.2 (br, 1H), 9.13 (br, 2H), 7.90 (d, J = 8.8 Hz, 1H), 7.14 (d, J = 2.4 Hz, 1H), 7.09 (d, J = 2.4 Hz, 1H), 6.62 (dd, J = 8.8, 2.4 Hz, 1H), 6.54 (d, J = 2.4 Hz, 1H); ^{13}C NMR (101 MHz, acetone-d_6) δ 165.0, 163.9, 161.7, 156.9, 142.3, 142.0, 129.6, 116.7, 109.6, 104.5, 103.9, 103.2, 102.2; HRMS (ESI) m/z calcd for $C_{13}H_9BrNO_4$ $[M+H]^+$: 321.9710 found 321.9707.

Synthesis of 5-(7-bromo-5-hydroxybenzo[*d*]oxazol-2-yl)benzene-1,3-diol (11: TKD147)

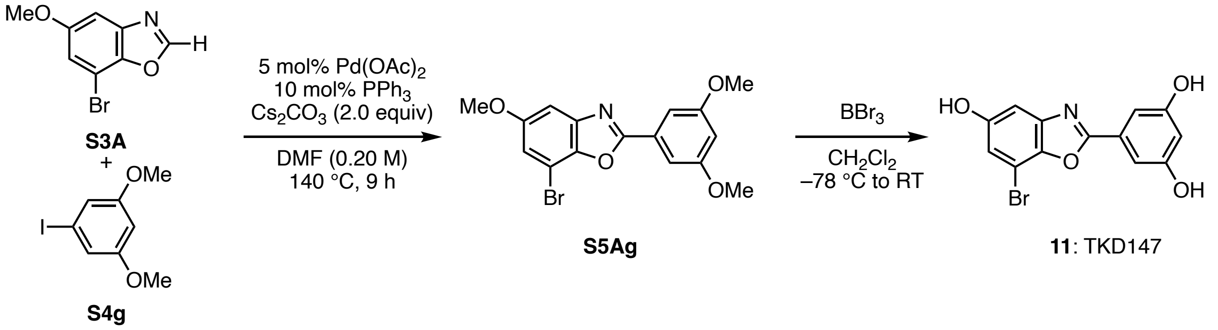

Following the General procedure A, **S3A** (45.6 mg, 0.20 mmol, 1.0 equiv) and **S4g** (52.8 mg, 0.20 mmol, 1.0 equiv) were reacted. Purification by PTLC (hexane/$CHCl_3$ = 1:2) afforded 7-bromo-2-(3,5-dimethoxyphenyl)-5-methoxybenzo[*d*]oxazole (**S5Ag**) as a white solid (32.1 mg, 44% yield).

Following the General procedure B, **S5Ag** (19.4 mg, 53 µmol) and BBr_3 (70 µL, 0.74 mmol, 13.9 equiv) were reacted. Purification by Isolera® (hexane/EtOAc = 9:1 to 1:4) to afford **11** (TKD147) as a white solid (17.1 mg, quant.). ^{1}H NMR (400 MHz, acetone-d_6) δ 8.88–8.73 (br, 3H), 7.24 (d, J = 2.0 Hz, 2H), 7.13 (d, J = 2.4 Hz, 1H), 7.10 (d, J = 2.4 Hz, 1H), 6.60 (t, J = 2.0 Hz, 1H); ^{13}C NMR (101 MHz, acetone-d_6) δ 164.5, 160.0, 156.6, 144.3, 143.8, 129.2, 117.3, 107.2, 106.8, 105.6, 102.2; HRMS (ESI) m/z calcd for $C_{13}H_9BrNO_4$ $[M+H]^+$: 321.9710 found 321.9707.

Synthesis of 3-(7-bromobenzo[*d*]oxazol-2-yl)phenol (12: TKD129)

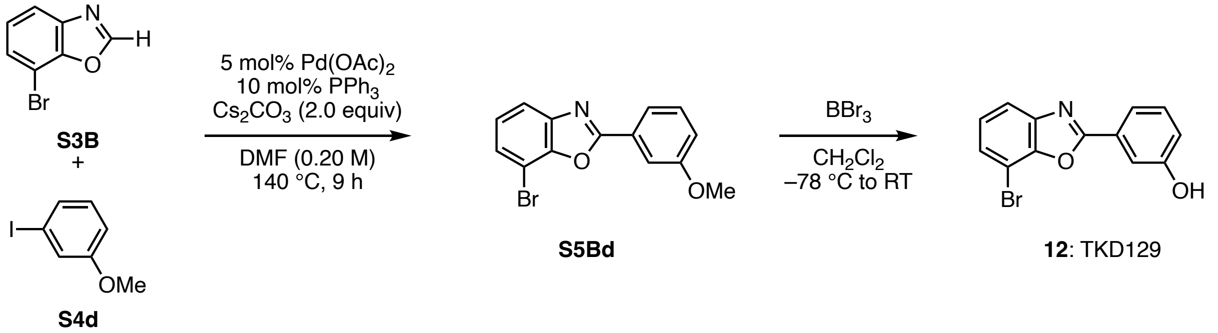

Following the General procedure A, **S3B** (39.4 mg, 0.20 mmol, 1.0 equiv) and **S4d** (46.8 mg, 0.20 mmol, 1.0 equiv) were reacted. Purification by PTLC (hexane/EtOAc = 7:1) afforded 7-bromo-2-(3-methoxyphenyl)benzo[*d*]oxazole (**S5Bd**) as a white solid (23.8 mg, 39% yield).

Following the General procedure B, **S5Bd** (16.5 mg, 54 µmol) and BBr_3 (40 µL, 0.42 mmol, 7.8 equiv) were reacted. Purification by Isolera® (hexane/EtOAc = 9:1 to 1:2) to afford **12** (TKD129) as a

white solid (15.5 mg, 98% yield). ^{1}H NMR (400 MHz, acetone-d_6) δ 8.87 (br, 1H), 7.81–7.69 (m, 3H), 7.60 (d, J = 8.0 Hz, 1H), 7.46 (t, J = 8.0 Hz, 1H), 7.36 (t, J = 8.0 Hz, 1H), 7.13 (dd, J = 8.0, 2.4 Hz, 1H); ^{13}C NMR (101 MHz, acetone-d_6) δ 163.8, 158.8, 149.7, 143.9, 131.4, 129.1, 128.6, 127.0, 120.3, 120.1, 119.8, 115.0, 102.8. HRMS (ESI) m/z calcd for $C_{13}H_{9}BrNO_{2}$ $[M+H]^+$: 289.9811 found 289.9810.

Synthesis of 2-(7-bromobenzo[*d*]oxazol-2-yl)benzene-1,3,5-triol (13: TKD100)

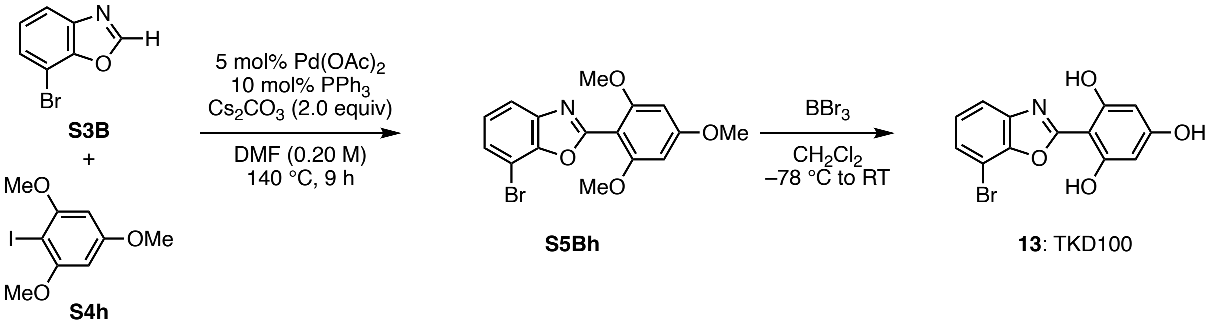

Following the General procedure A, **S3B** (39.7 mg, 0.20 mmol, 1.0 equiv) and **S4h** (59.0 mg, 0.20 mmol, 1.0 equiv) were reacted. Purification by PTLC (hexane/EtOAc = 6:1) afforded 7-bromo-2-(2,4,6-trimethoxyphenyl)benzo[*d*]oxazole (**S5Bh**) as a white solid (32.7 mg, 45% yield).

Following the General procedure B, **S5Bh** (19.6 mg, 54 µmol) and BBr_3 (20 µL, 0.21 mmol, 3.9 equiv) were reacted. Purification by Isolera® (EtOAc) to afford **13** (TKD100) as a white solid (17.2 mg, 99% yield). ^{1}H NMR (400 MHz, acetone-d_6) δ 10.70 (s, 2H), 9.14 (s, 1H), 7.72 (dd, J = 8.0, 1.2 Hz, 1H), 7.57 (dd, J = 8.0, 1.2 Hz, 1H), 7.37 (t, J = 8.0 Hz, 1H), 6.14 (s, 2H); ^{13}C NMR (101 MHz, acetone-d_6) δ 165.0, 164.0, 161.7, 147.8, 140.7, 128.3, 127.2, 118.2, 102.8, 96.4, 93.3; HRMS (ESI) m/z calcd for $C_{13}H_{9}BrNO_{4}$ $[M+H]^+$: 321.9710 found 321.9709.

Synthesis of 7-bromo-2-(4-hydroxy-2,6-dimethylphenyl)benzo[*d*]oxazol-5-ol (14: TKD146)

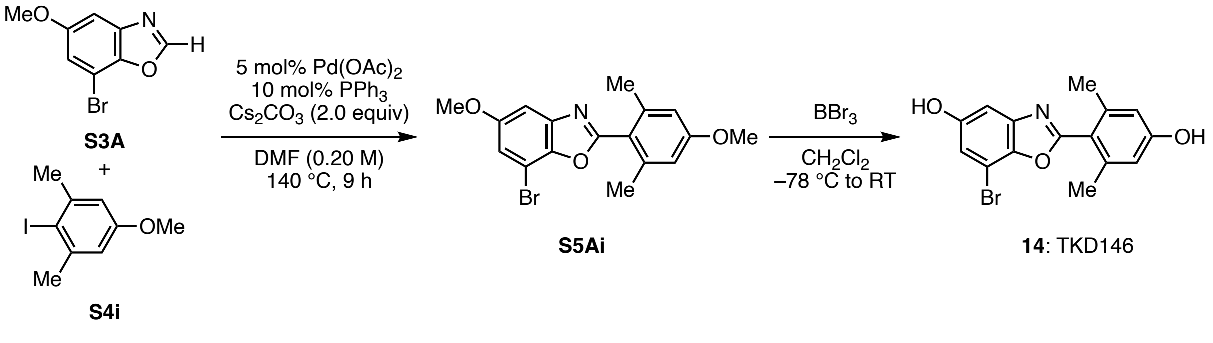

Following the General procedure A, **S3A** (45.6 mg, 0.20 mmol, 1.0 equiv) and **S4i** (52.4 mg, 0.20 mmol, 1.0 equiv) were reacted. Purification by PTLC (hexane/$CHCl_3$ = 1:1) afforded 7-bromo-5-methoxy-2-(4-methoxy-2,6-dimethylphenyl)benzo[*d*]oxazole (**S5Ai**) as a white solid (18.1 mg, 25% yield).

Following the General procedure B, **S5Ai** (15.9 mg, 44 µmol) and BBr_3 (40 µL, 0.42 mmol, 9.6 equiv) were reacted. Purification by Isolera® (hexane/EtOAc = 9:1 to 1:2) to afford **14** (TKD146) as a

white solid (13.0 mg, 89% yield). ^{1}H NMR (400 MHz, acetone-d_6) δ 8.81 (br, 1H), 8.75 (br, 1H), 7.16 (d, J = 2.0 Hz, 1H), 7.11 (d, J = 2.0 Hz, 1H), 6.70 (s, 2H), 2.29 (s, 6H); ^{13}C NMR (101 MHz, acetone-d_6) δ 165.0, 159.9, 156.3, 143.8, 143.7, 141.4, 119.5, 117.0, 115.8, 105.5, 102.1, 20.9; HRMS (ESI) m/z calcd for $C_{15}H_{13}BrNO_3$ $[M+H]^+$: 334.0073 found 334.0071.

Synthesis of 7-bromo-2-(4-hydroxy-3,5-dimethylphenyl)benzo[*d*]oxazol-5-ol (15: TKD152)

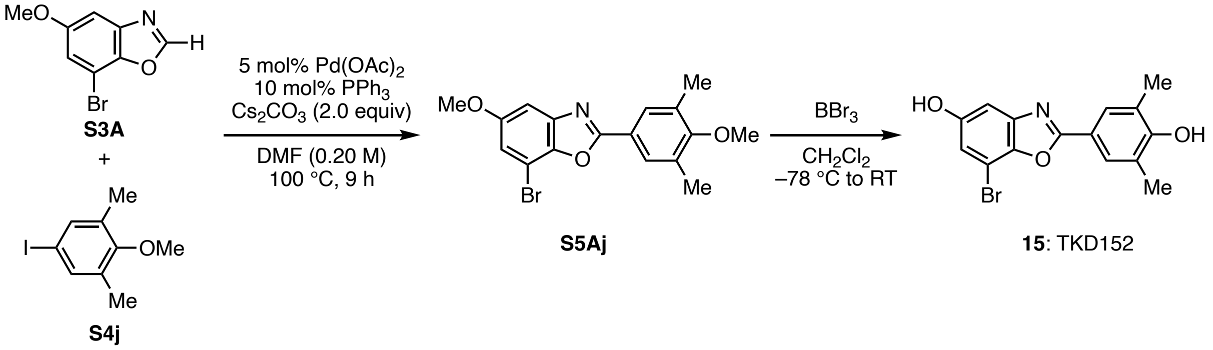

Following the General procedure A, **S3A** (45.6 mg, 0.20 mmol, 1.0 equiv) and **S4j** (52.5 mg, 0.20 mmol, 1.0 equiv) were reacted at 100 °C. Purification by PTLC (hexane/EtOAc = 6:1) afforded 7-bromo-5-methoxy-2-(4-methoxy-3,5-dimethylphenyl)benzo[*d*]oxazole (**S5Aj**) as a white solid (40.2 mg, 55% yield).

Following the General procedure B, **S5Aj** (19.2 mg, 53 μmol) and BBr_3 (60 μL, 0.63 mmol, 11.9 equiv) were reacted. Purification by Isolera® (hexane/EtOAc = 9:1 to 2:5) to afford **15** (TKD152) as a white solid (11.2 mg, 63% yield). ^{1}H NMR (400 MHz, acetone-d_6) δ 8.75 (br, 1H), 8.12 (br, 1H), 7.85 (s, 2H), 7.08 (d, J = 2.0 Hz, 1H), 7.04 (d, J = 2.0 Hz, 1H), 2.35 (s, 6H); ^{13}C NMR (151 MHz, acetone-d_6) δ 165.1, 158.0, 156.4, 144.7, 143.8, 129.0, 125.7, 118.7, 116.4, 105.2, 101.9, 16.6; HRMS (ESI) m/z calcd for $C_{15}H_{13}BrNO_3$ $[M+H]^+$: 334.0073 found 334.0070.

Synthesis of 7-bromo-2-(3-ethyl-4-hydroxyphenyl)benzo[*d*]oxazol-5-ol (16: TKD150)

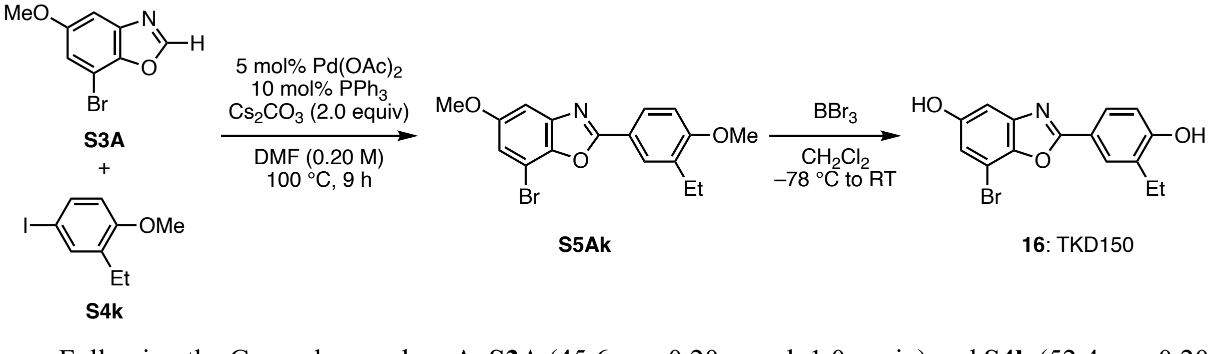

Following the General procedure A, **S3A** (45.6 mg, 0.20 mmol, 1.0 equiv) and **S4k** (52.4 mg, 0.20 mmol, 1.0 equiv) were reacted at 100 °C. Purification by PTLC (hexane/EtOAc = 7:1) afforded 7-bromo-2-(3-ethyl-4-methoxyphenyl)-5-methoxybenzo[*d*]oxazole (**S5Ak**) as a white solid (53.1 mg, 73% yield).

Following the General procedure B, **S5Ak** (36.3 mg, 100 µmol) and BBr_3 (93 µL, 1.0 mmol, 10.0 equiv) were reacted. Purification by Isolera® (hexane/EtOAc = 9:1 to 2:5) to afford **16** (TKD150) as a white solid (30.4 mg, 91%). ^1H NMR (400 MHz, acetone-d_6) δ 9.14 (br, 1H), 8.78 (br, 1H), 8.00 (s, 1H), 7.93 (dd, J = 8.4, 2.0 Hz, 1H), 7.10–7.00 (m, 3H), 2.75 (q, J = 7.6 Hz, 2H), 1.27 (t, J = 7.6 Hz, 3H); ^{13}C NMR (101 MHz, acetone-d_6) δ 165.1, 159.6, 156.4, 144.7, 143.8, 132.3, 129.7, 127.8, 118.9, 116.4, 116.3 105.3, 101.9, 23.8, 14.3; HRMS (ESI) m/z calcd for $C_{15}H_{13}BrNO_3$ $[M+H]^+$: 334.0073 found 334.0070.

4. RT-QuIC assay

Preparation of recombinant human α-Synuclein protein

Recombinant human *α*-Synuclein (residues 1-140, NCBI Reference Sequence: NM_000345) was constructed in a pET21a vector (Merck KgaA, Darmstadt, Germany) to express in Escherichia coli. The plasmid was transformed into *E.coli* BL21 (DE3) (Nippongene, Tokyo, Japan). Expression was induced for 12 h at 25 °C using final concentration of 0.5 mM isopropyl β-D-1-thiogalactopyranoside (IPTG) and then cell pellets were collected by centrifugation. Pellets were lysed using sonication, and then the supernatant was added to NaCl (final concentration 0.5 M) and heated at 85 °C for 10 min. After centrifugation, the pH of the supernatant was reduced to pH 3.5 using HCl, and the mixture stirred at room temperature for 10 min and then centrifuged. The pH of the supernatant was then increased to pH 7.0 with NaOH. Recombinant *α*-Synuclein was then purified by anion exchange chromatography using Q Sepharose Fast Flow column and Mono Q column (Cytiva, MA, USA). Protein concentrations were determined using BCA Protein Assay Kit (Thermo Fisher Scientific, MA, USA) with Bovine Serum Albumin as a standard.

Real time quaking induced conversion (RT-QuIC)

RT-QuIC plate was pre-filled with 40 mg of 0.4 mm zirconia-silica beads. The reaction mixture was composed from 0.1 mg/mL recombinant aSN, 10 μM Thioflavin T (FUJIFILM Wako Pure Chemical Corporation, Osaka, Japan), 10 μM DMSO solution of synthesized compounds in 50 mM phosphate buffer (pH 8.0) (FUJIFILM Wako Pure Chemical Corporation). After adding 97.5 μL of the reaction mixture to the plate, 2.5 μL of pre-formed fibril at 100 ng/mL was added. Three controls were performed for every experiment: 1) absence of pre-formed fibril 2) PBS control (absence of compound) and 3) DMSO control (absence of compound, addition of DMSO). The plate was tightly sealed and repeatedly shaken for 1 minute with subsequent resting for 14 minutes. Fluorescence intensity of Thioflavin T (excitation: 450 nm / emission: 480 nm) was recorded every 15 minutes during the assay, which completed after 60 hours. Assay was performed in duplicate for each sample and time to reach fluorescence intensity of 2000 au was referred as t_{2000}, which was expressed as a ratio by dividing with t_{2000} for PA86. Of these duplicate, shorter t_{2000} value was adopted in order to get enough sensitivity in the screening process. Control runs did not show any indication of accelerated aggregation due to DMSO as shown in Figure S1 (average ratio for PBS conrol = 1.54, DMSO control = 1.50). Pre-formed fibril was prepared as well as RT-QuIC method.

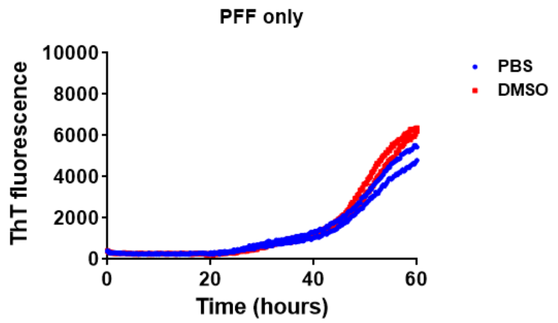

Figure S1: RT-QuIC assay results for PBS control and DMSO control

Preparation of pre-formed fibril

Pre-formed fibril was prepared similarly with RT-QuIC method in the absence of Thioflavin T and synthesized compound described above. After 60 hours incubation, the reaction mixture was centrifuged at 20,000 × g, 4 °C for 20 minutes. The supernatant was removed and the pellet was washed with 1 mL of 20 mM Tris-HCl (Nippongene) and 0.1 M Sodium Chloride (Promega, WI, USA). After centrifugation at 20,000 × g, 4 °C for 20 minutes, the supernatant was resuspended in the same buffer and sonicated at high power for 1 minute on ice (Sonicbio Co., Ltd, Kanagawa, Japan). The protein concentration was evaluated by MicroBCA Proein Assay Kit (Thermo Fisher Scientific).

Effect of aggregator compounds towards fluorescence of Thioflavin T

To test the influence of aggregator compound towards the fluorescence signal of Thioflavin T, a solution containing 10 µM of compound, 10 µM Thioflavin T, and variable amount of aSN pre-formed fibril was prepared (Figure S2). Compared to DMSO control, a marginal increase in Thioflavin T fluorescence signal was observed for TKD149 in absence or low concentration of aSN PFF. However, at higher PFF concentration, no significant increase of ThT fluorescence signal was observed for both TKD149 and TKD150 in comparison to DMSO control. Together, these results suggest that the fluorescence signal from ThT is unaffected in the presence of proaggregator compound.

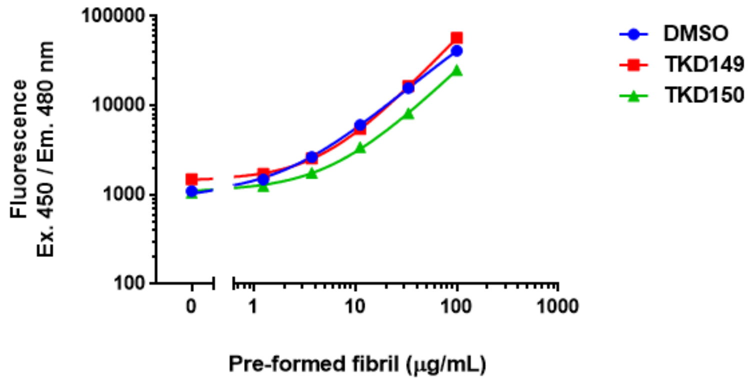

Figure S2: Influence of TKD149 or TKD150 towards fluorescence signal of Thioflavin T

5. Binding assay with Affinity Selection-Mass Spectrometry (AS-MS)

The binding assay using AS-MS was performed at room temperature in a final volume of 10 µl using the recombinant α-synuclein fibril protein. The protein (200 nM or 400 nM) was incubated with test compounds at 8 serial diluted concentrations for 1 hour in assay buffer (50 mM Tris-HCl (pH 8.0), 150 mM NaCl, 0.005% Tween20). For the estimation of background, only the test compounds were added to the assay buffer. The reaction was terminated by separation of bound and free compounds at 1 hour using a 384-well filter plate (5085, Pall Corp, NY, USA) packed with gel filtration resin. Then, 5 µl of flow-through fraction was mixed with 50 µl of solvent solution (water, acetonitrile and methanol (6:1:1) containing 1% formic acid) to denature the protein–compound complexes. Liberated compounds were quantified by a RapidFire-MS/MS system equipped with an electrospray ionization interface (Agilent, CA, USA). Compounds were separated on a reversed-phase column (C18, Agilent, CA, USA) with a mobile phase consisting of solvent A (10 mM ammonium formate containing 0.2% formic acid) and solvent B (acetonitrile containing 0.2% formic acid). The mass transitions (Q1/Q3) used for TKD149 and TKD152 (positive mode), Albendazole (Internal Standard of positive mode) were m/z 320.0/211.6, 334.01/318.9, and 266.1/234.1 respectively. All data was calculated as the peak area by Agilent software and divided by albendazole (IS) area. Using standard plot, the area ratio was converted to concentration and the concentration was analyzed with Prism 6 (Version 6.07, Graph Pad Software, CA, USA). The binding rate constants, apparent equilibrium dissociation constants (K_d) were calculated with Prism 6 using the nonlinear iterative curve-fitting computer program. Data are shown as the mean ± standard deviation (SD).

AS-MS assay results for TKD152 is shown below using 200 nM of aSN fibril (Figure S3A) and 400 nM of aSN fibril (Figure S3B). When 200 nM of fibril was used, the results showed large variation, while less variation was observed when 400 nM of fibril was used.

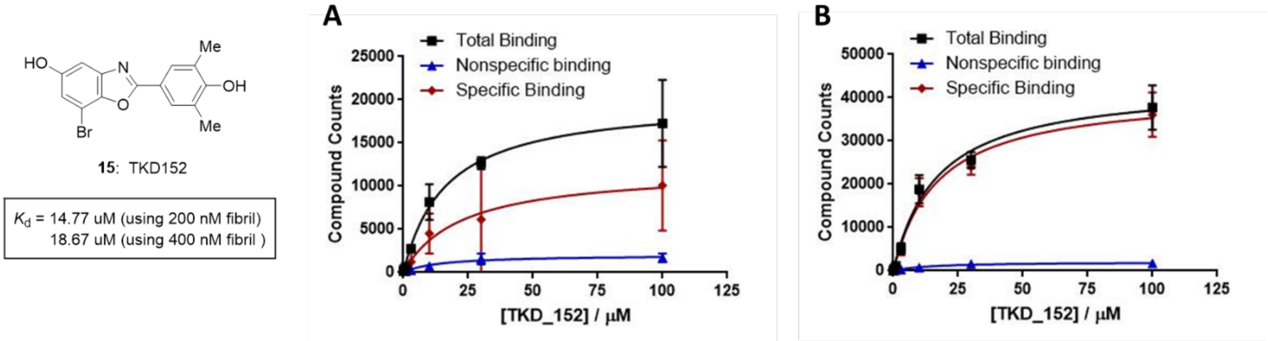

Figure S3: AS-MS assay results for TKD152

6. Transmission electron microscopy (TEM) analysis

RT-QuIC assay was performed for 60 hours in the absence of Thioflavin T. The reaction mixture was dispersed on 400-mesh carbon-supported copper grid at room temperature for 10 seconds. Subsequently, negative staining was performed by 2% uranyl acetate at room temperature for 10 seconds. The images were obtained by HITACHI-H7600 (Hitachi High-Tech Corporation) at 10 kV (Figure S4). The bar in each picture represents 200 nm length.

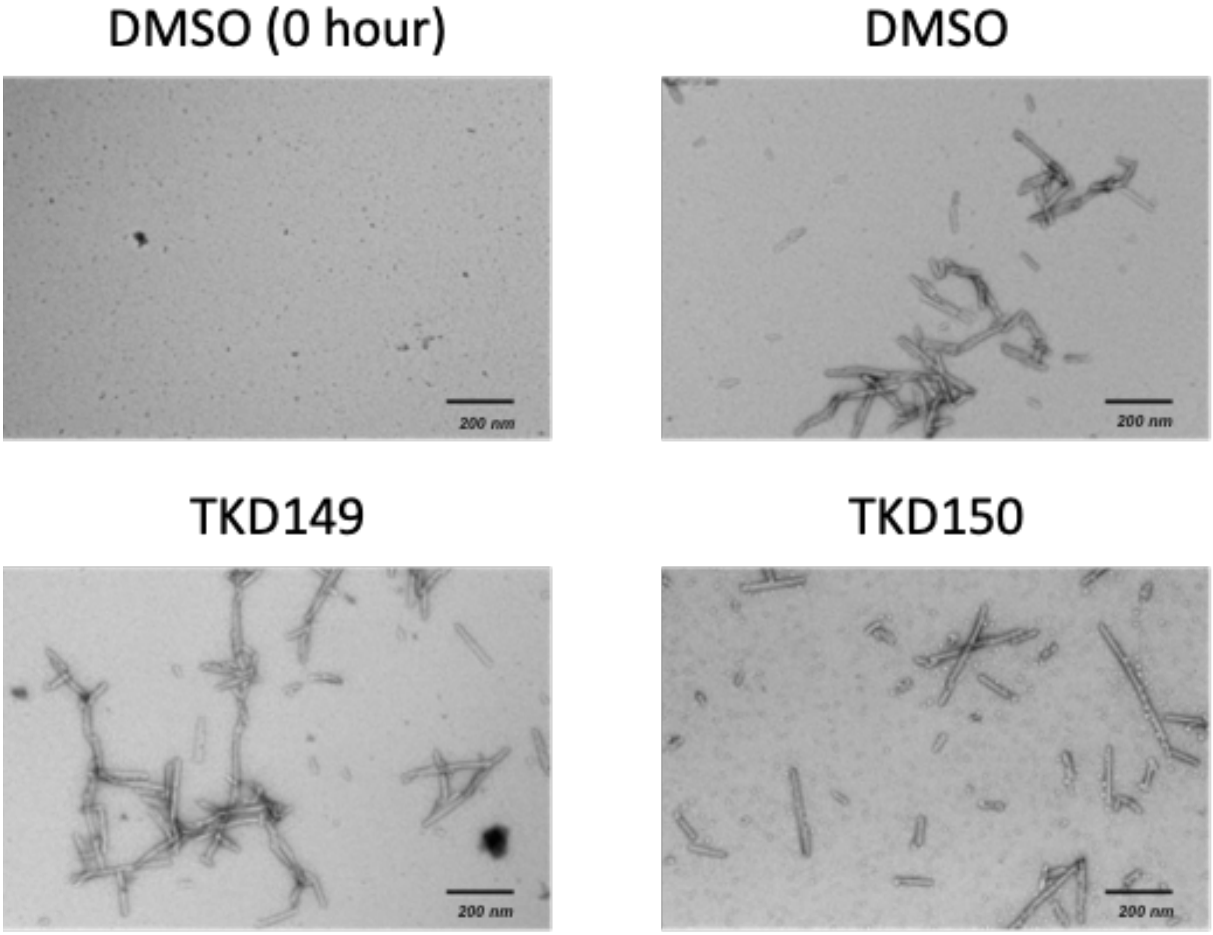

Figure S4. TEM images

7. Particle size analysis

In order to investigate whether TKD150 itself forms colloidal particles, nanoparticle tracking analysis was performed on Nanosight (Malvern Panalytical, UK). Samples (10 mM DMSO solution of compound diluted in RT-QuIC reaction buffer without α-synuclein monomer and PFF, final concentration of 10μM) were incubated for 60 hours at 37 °C under agitating condition used for RT-QuIC assay. Upon completion of agitation, a set of 5 samples were pooled as 1 sample and measured using Nanosight under static conditions. Latex beads with diameter of 100 nm were used as a reference sample. As shown in Figure S5, both DMSO and TKD-150 samples did not show presence of colloids.

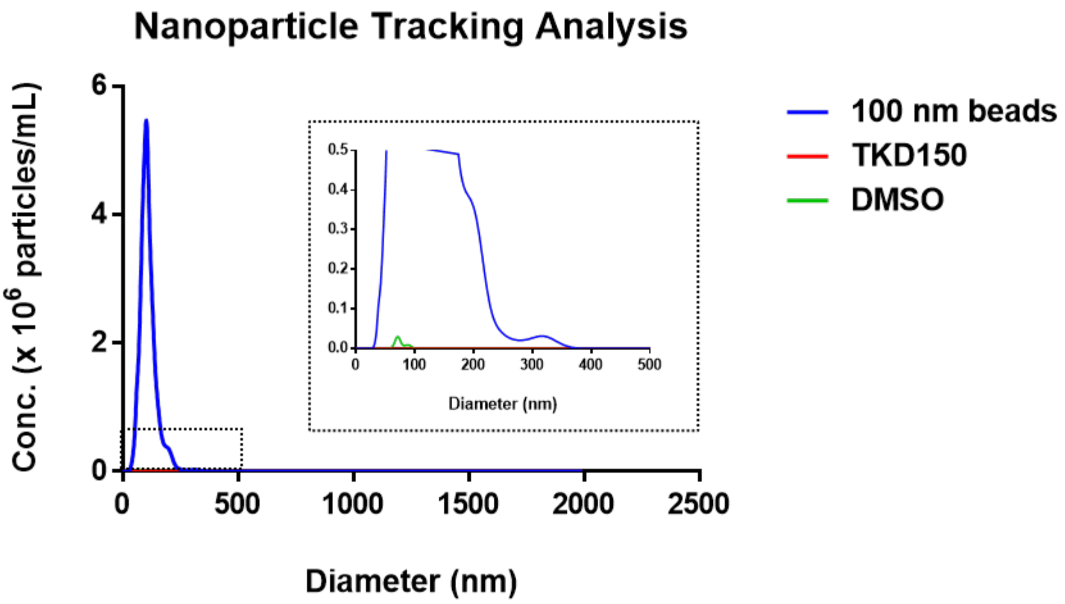

Figure S5: Particle size analysis of TKD-150

8. Aggregation Advisor results

Shown below are results obtained from the online database (http://advisor.bkslab.org/), indicating that none of the compounds are similar to previously known aggregators (query setting of affinity range > 10 μM)

1: PA86

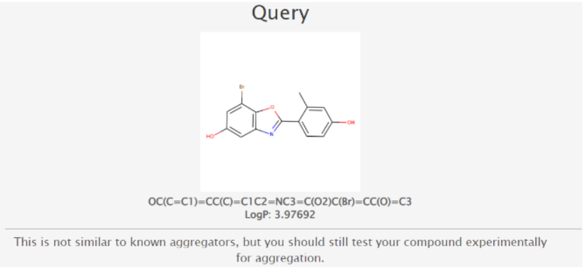

TKD35

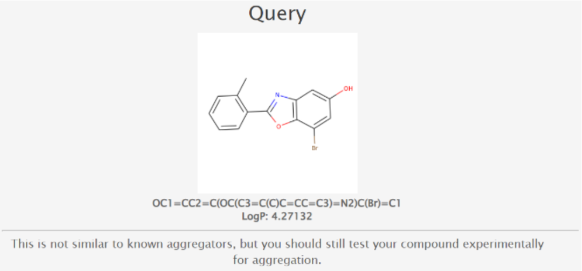

TKD1

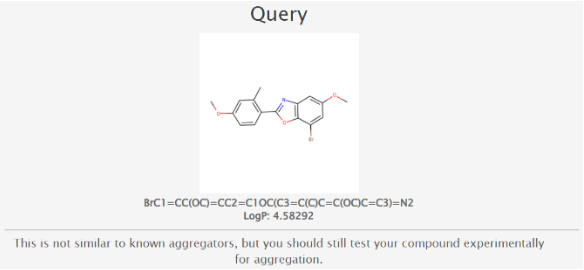

TKD25

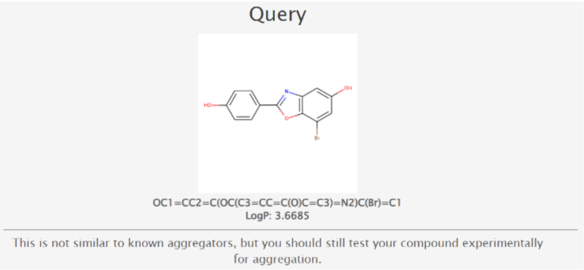

TKD33

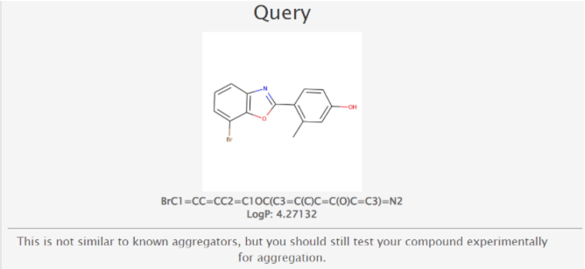

TKD26

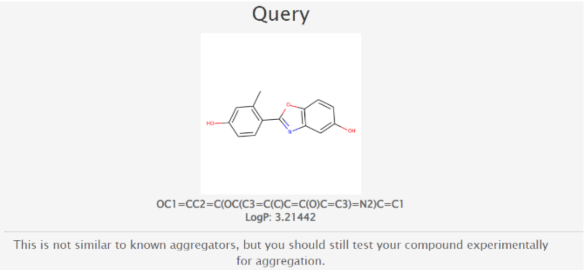

TKD10

Query

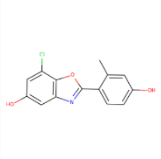

OC(C=C1)=CC(C)=C1C2=NC3=C(O2)C(Cl)=CC(O)=C3
LogP: 3.86782

This is not similar to known aggregators, but you should still test your compound experimentally for aggregation.

TKD66

Query

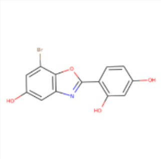

OC1=C(C=CC(O)=C1)C2=NC3=C(O2)C(Br)=CC(O)=C3
LogP: 3.3741

This is not similar to known aggregators, but you should still test your compound experimentally for aggregation.

TKD125

Query

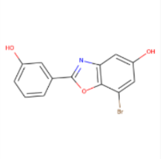

BrC1=CC(O)=CC2=C1OC(C3=CC(O)=CC=C3)=N2
LogP: 3.6685

This is not similar to known aggregators, but you should still test your compound experimentally for aggregation.

TKD147

Query

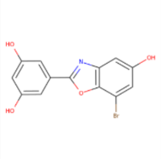

OC1=CC2=C(OC(C3=CC(O)=CC(O)=C3)=N2)C(Br)=C1
LogP: 3.3741

This is not similar to known aggregators, but you should still test your compound experimentally for aggregation.

TDK149

Query

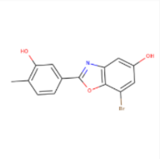

BrC1=CC(O)=CC2=C1OC(C3=CC(O)=C(C)C=C3)=N2
LogP: 3.97692

This is not similar to known aggregators, but you should still test your compound experimentally for aggregation.

TKD129

Query

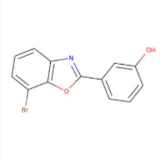

BrC1=CC=CC2=C1OC(C3=CC=CC(O)=C3)=N2
LogP: 3.9629

This is not similar to known aggregators, but you should still test your compound experimentally for aggregation.

TKD100

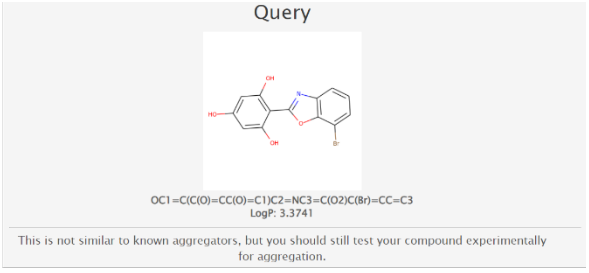

TKD152

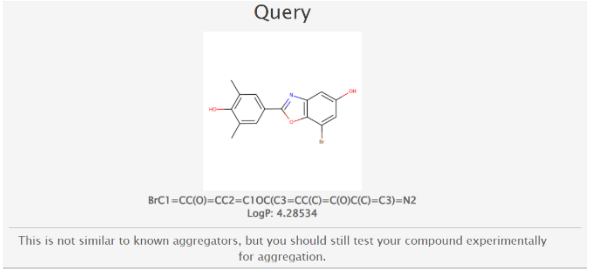

TKD146

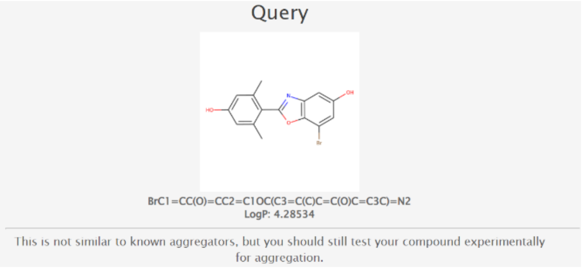

TKD150

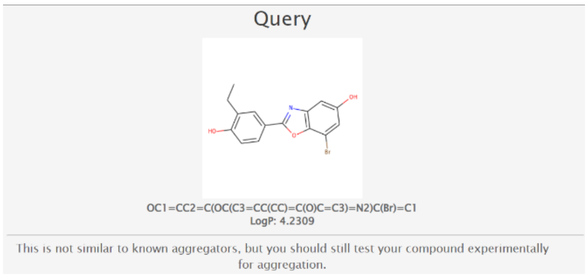

7. References

[1] Perry, G. J. P.; Quibell, J. M.; Panigrahi, A.; Larrosa, I. Transition-Metal-Free Decarboxylative Iodination: New Routes for Decarboxylative Oxidative Cross-Couplings. *J. Am. Chem. Soc.* **2017**, *139*, 11527–11536.

[2] Jiang, M.; Yang, H.; Jin, Y.; Ou, L.; Fu, H. Visible-Light-Induced Decarboxylative Iodination of Aromatic Carboxylic Acids. *Synlett* **2018**, *29*, 1572–1577.

[3] Khatri, H. R.; Zhu, J. Synthesis of Complex *Ortho*-Allyliodoarenes by Employing the Reductive Iodonio-Claisen Rearrangement. *Chem. Eur. J.* **2012**, *18*, 12232–12236.

[4] Alberico, D.; Rudolph, A.; Lautens, M. Synthesis of Tricyclic Heterocycles via a Tandem Aryl Alkylation/Heck Coupling Sequence. *J. Org. Chem.* **2007**, *72*, 775–781.

[5] Bugarin, A.; Connell, B. T. Chiral Nickel(II) and Palladium(II) NCN-Pincer Complexes Based on Substituted Benzene: Synthesis, Structure, and Lewis Acidity. *Organometallics* **2008**, *27*, 4357–4369.

[6] Zhao, W.; Huang, L.; Guan, Y.; Wulff, W. D. Three-Component Asymmetric Catalytic Ugi Reaction–Concinnity from Diversity by Substrate-Mediated Catalyst Assembly. *Angew. Chem., Int. Ed.* **2014**, *53*, 3436–3441.

[7] Dörr, M.; Lips, S.; Martínez-Huitle, C. A.; Schollmeyer, D.; Franke, R.; Waldvogel, S. R. Synthesis of Highly Functionalized *N*,*N*-Diarylamides by an Anodic C,N-Coupling Reaction. *Chem. Eur. J.* **2019**, *25*, 7835–7838.

[8] Le, T. G.; Kundu, A.; Ghoshal, A.; Nguyen, N. H.; Preston, S.; Jiao, Y.; Ruan, B.; Xue, L.; Huang, F.; Keiser, J.; Hofmann, A.; Chang, B. C. H.; Garcia-Bustos, J.; Wells, T. N. C.; Palmer, M. J.; Jabbar, A.; Gasser, R. B.; Baell, J. B. Structure-Activity Relationship Studies of Tolfenpyrad Reveal Subnanomolar Inhibitors of Haemonchus Contortus Development. *J. Med. Chem.* **2019**, *62*, 1036–1053.

[9] Castanet, A.-S.; Colobert, F.; Broutin, P.-E. Mild and Regioselective Iodination of Electron-Rich Aromatics with *N*-Iodosuccinimide and Catalytic Trifluoroacetic Acid. *Tetrahedron Lett.* **2002**, *43*, 5047–5048.

[10] Song, S.; Sun, X.; Li, X.; Yuan, Y.; Jiao, N. Efficient and Practical Oxidative Bromination and Iodination of Arenes and Heteroarenes with DMSO and Hydrogen Halide: A Mild Protocol for Late-Stage Functionalization. *Org. Lett.* **2015**, *17*, 2886–2889.

[11] Pivsa-Art, S.; Satoh, T.; Kawamura, Y.; Miura, M.; Nomura, M. Palladium-Catalyzed Arylation of Azole Compounds with Aryl Halides in the Presence of Alkali Metal Carbonates and the Use of Copper Iodide in the Reaction. *Bull. Chem. Soc. Jpn.* **1998**, *71*, 467–473.

8. ^{1}H and ^{13}C NMR Spectra

^{1}H NMR of **S3A** (400 MHz, $CDCl_3$)

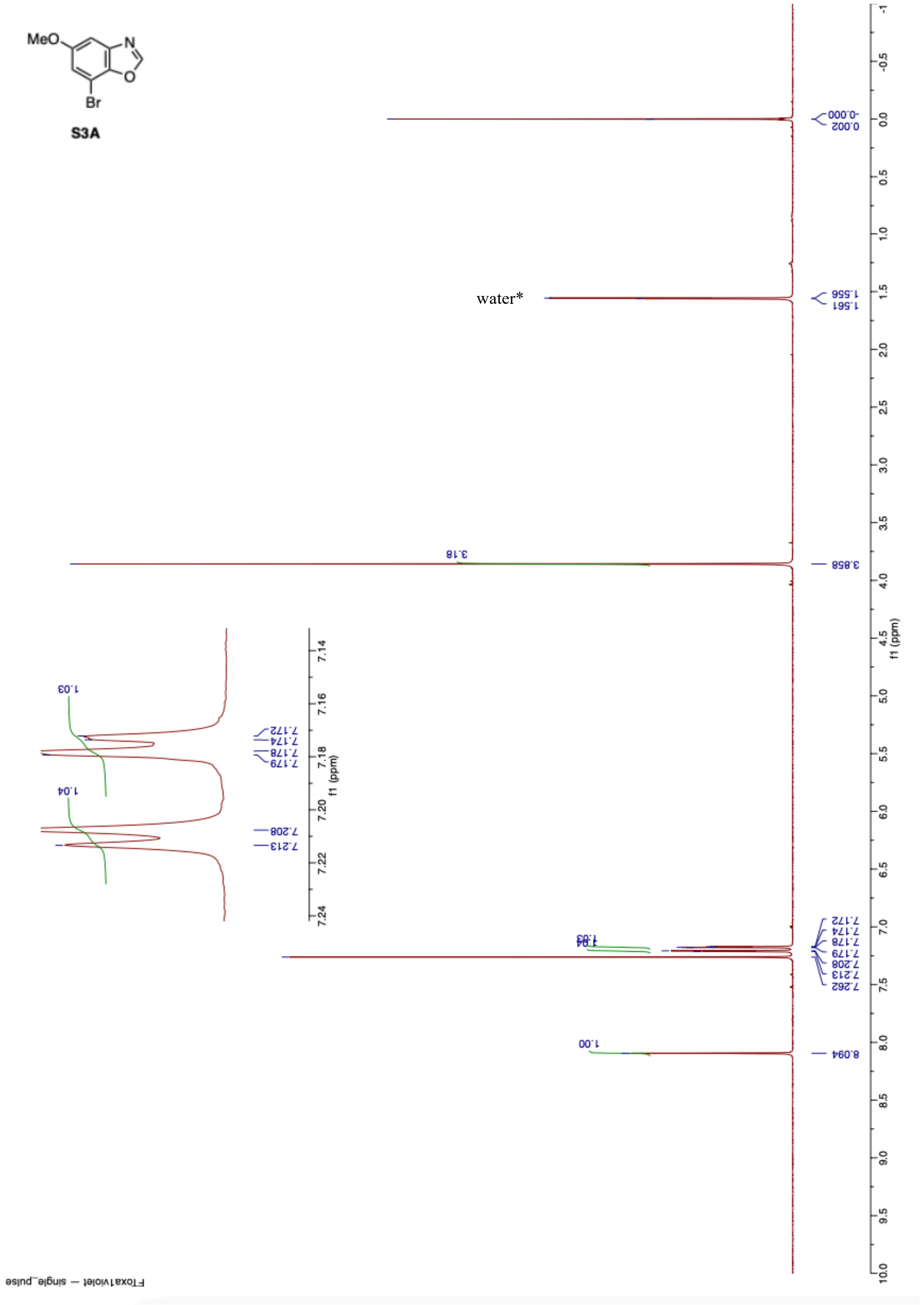

^{13}C NMR of **S3A** (101 MHz, $CDCl_3$)

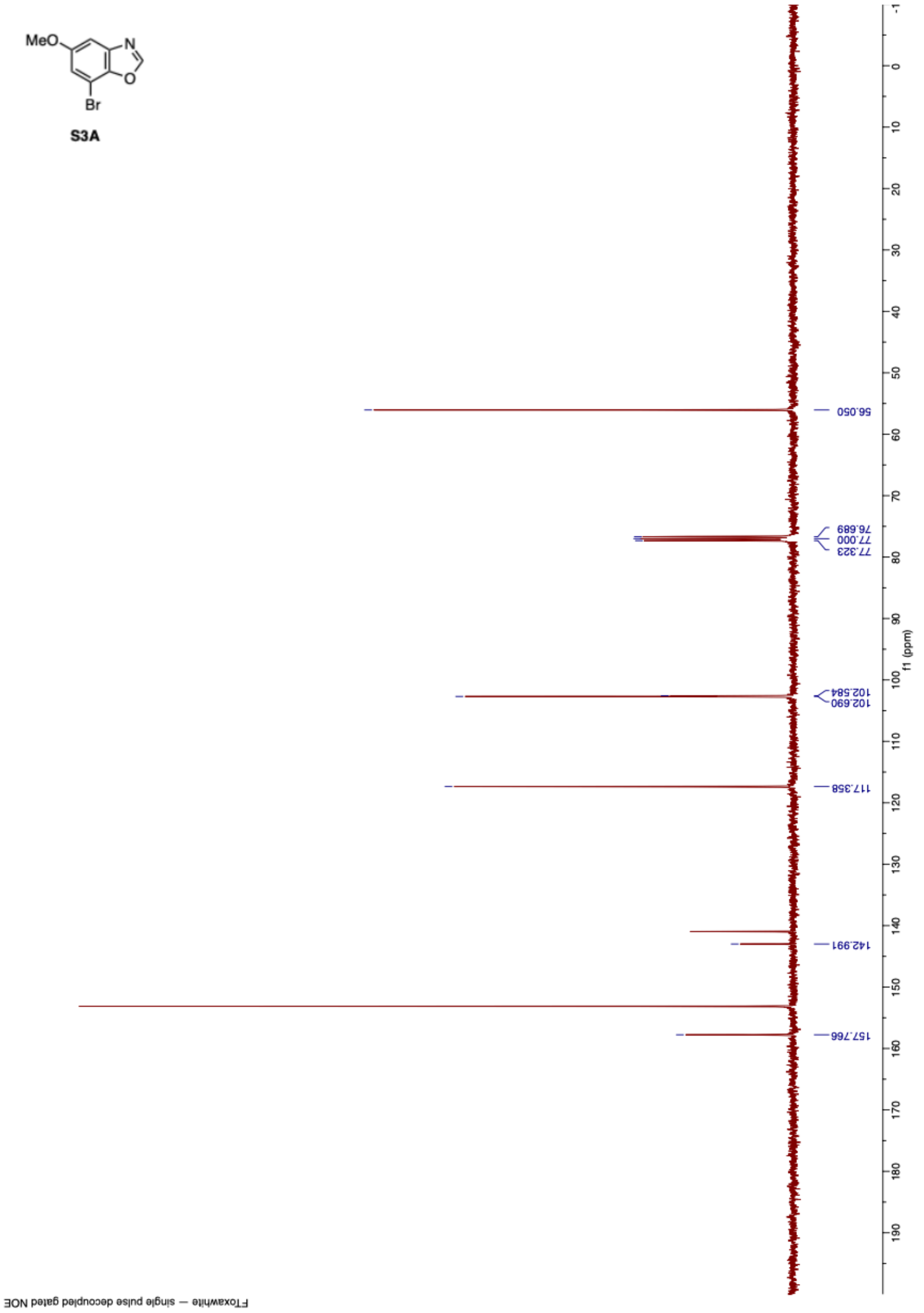

^{1}H NMR of **S3D** (400 MHz, $CDCl_3$)

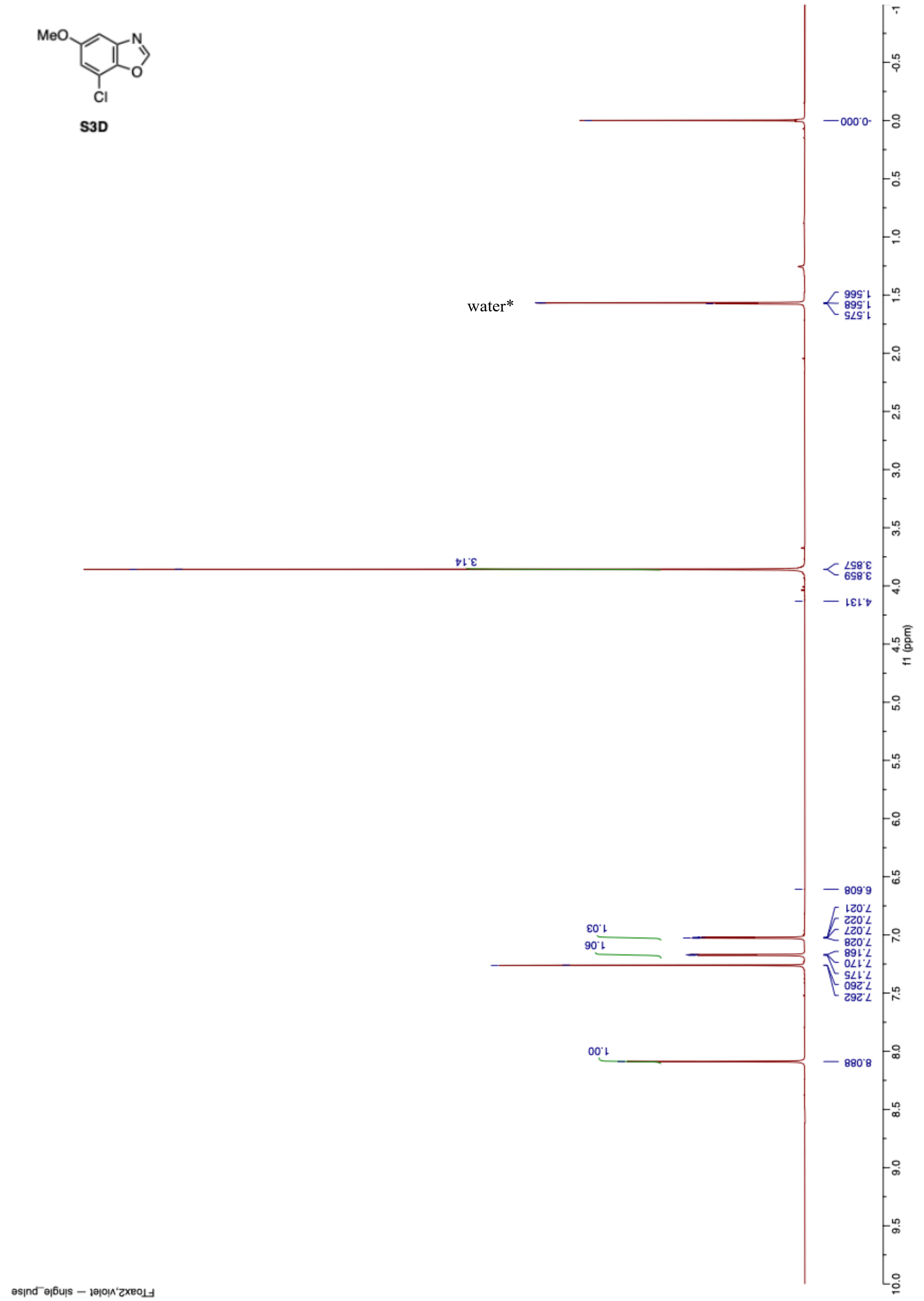

^{13}C NMR of **S3A** (101 MHz, $CDCl_3$)

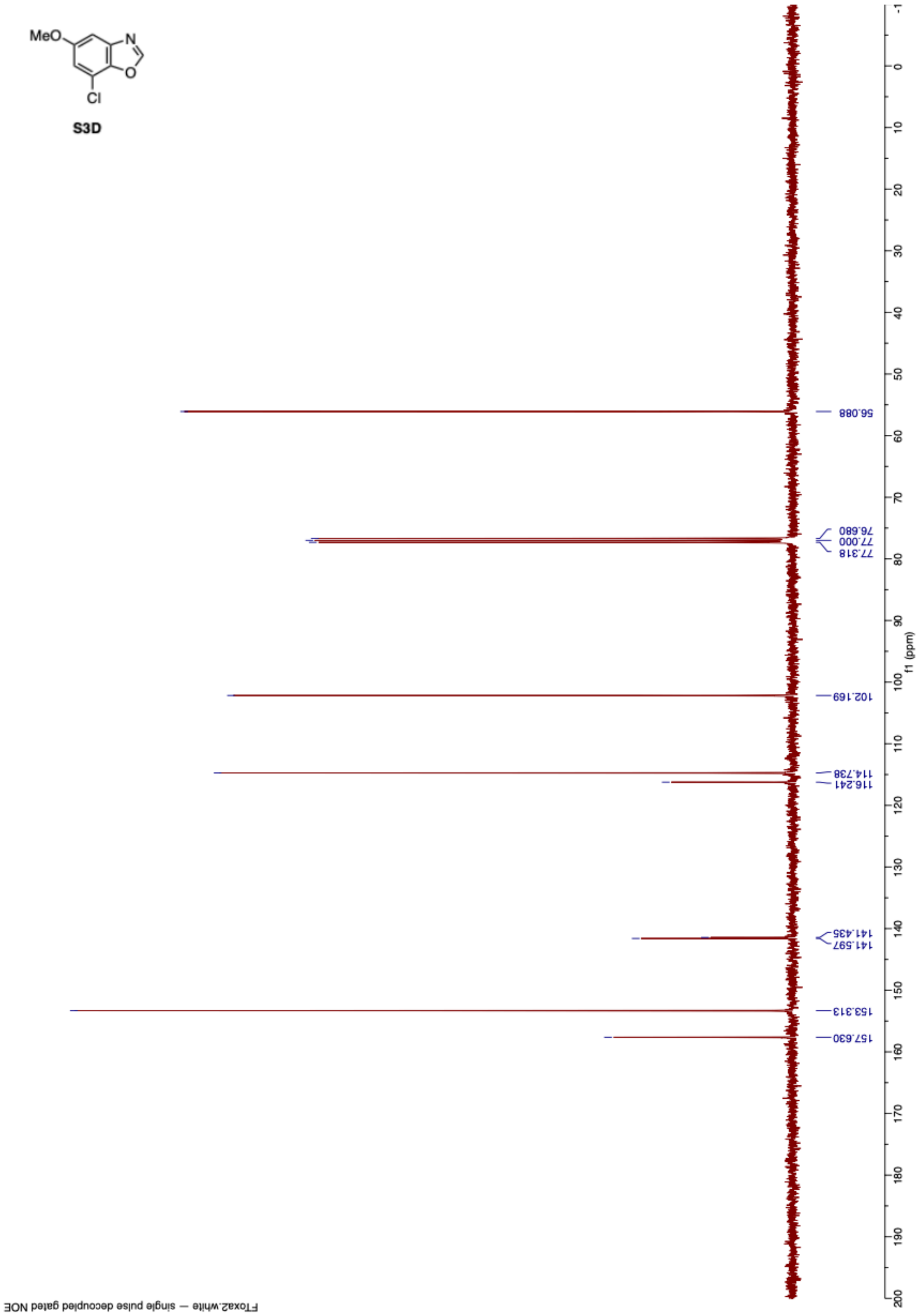

^{1}H NMR of **TKD1** (400 MHz, $CDCl_3$)

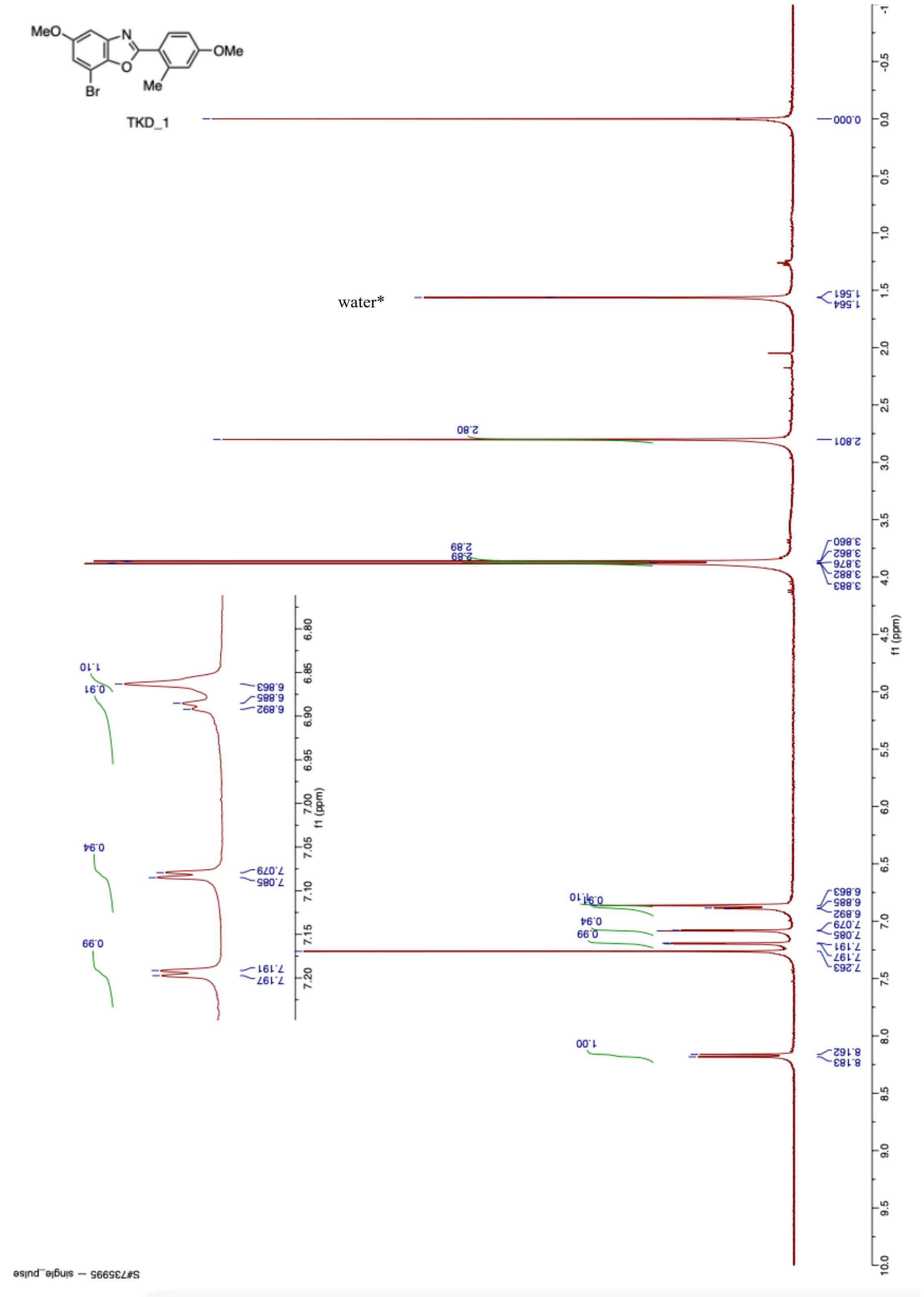

^{13}C NMR of **TKD1** (101 MHz, $CDCl_3$)

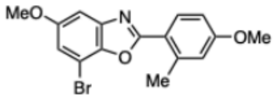

TKD_1

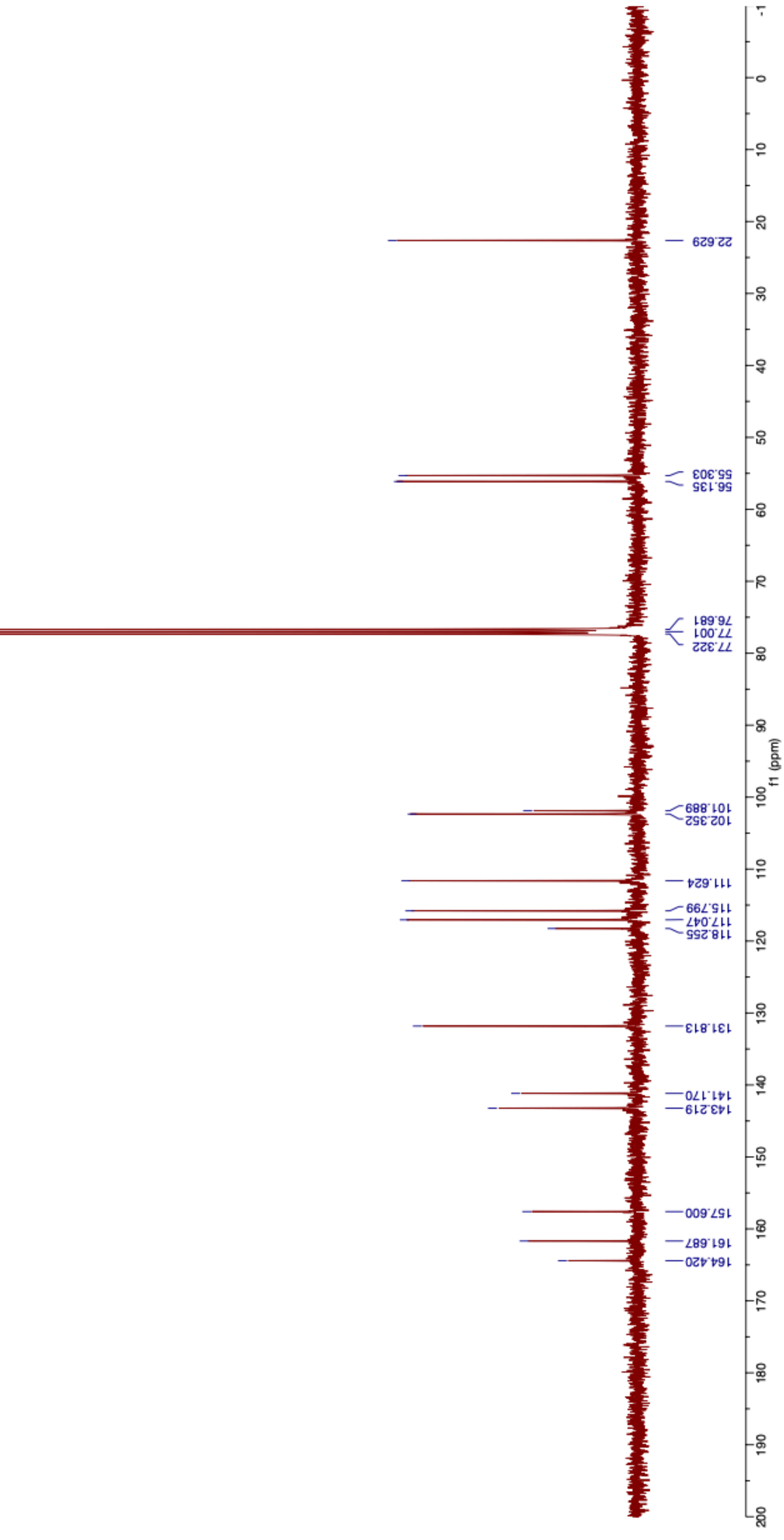

^{1}H NMR of **PA86** (400 MHz, acetone-d_6)

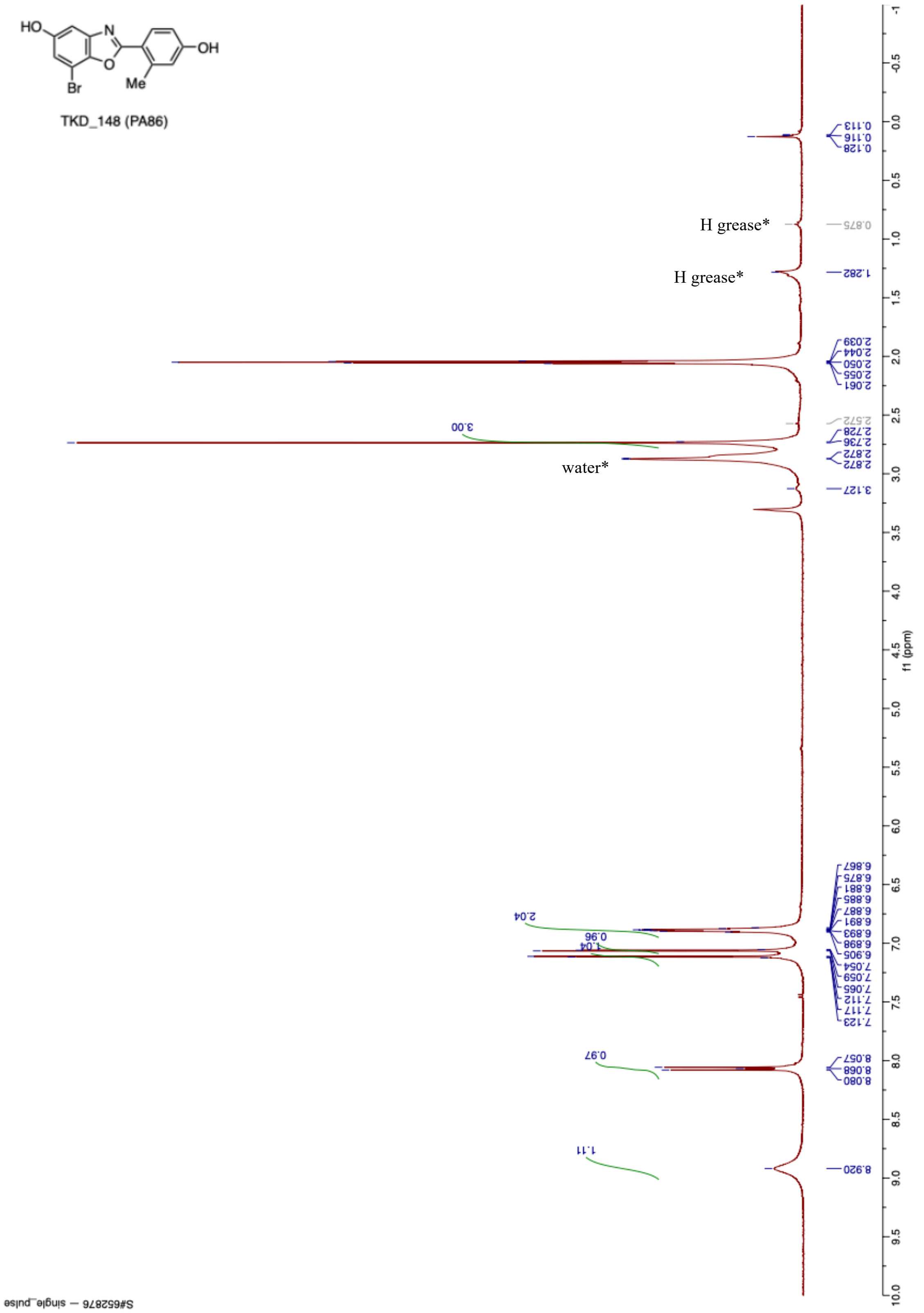

^{13}C NMR of **PA86** (101 MHz, acetone-d_6)

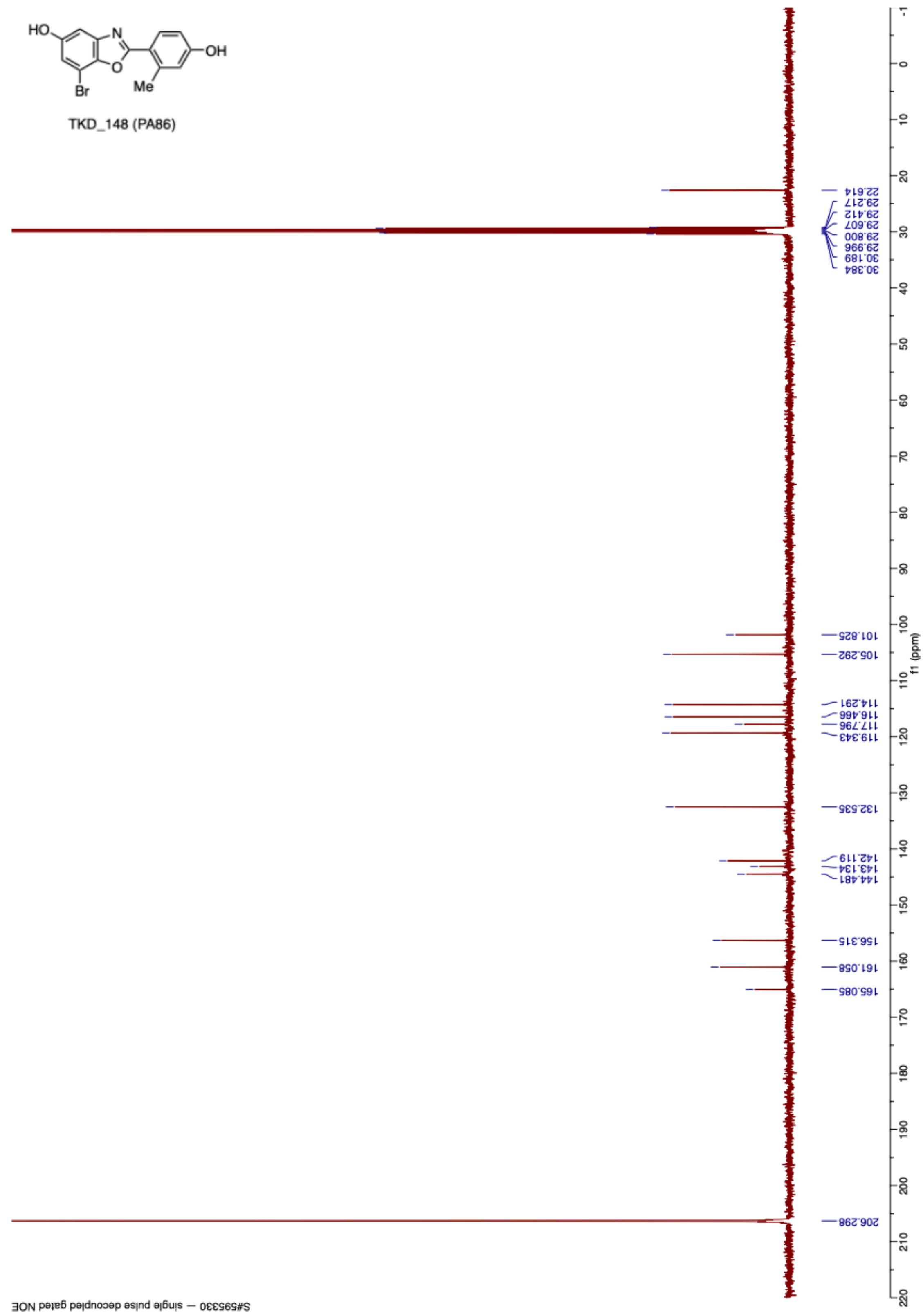

^{1}H NMR of **TKD33** (400 MHz, acetone-d_6)

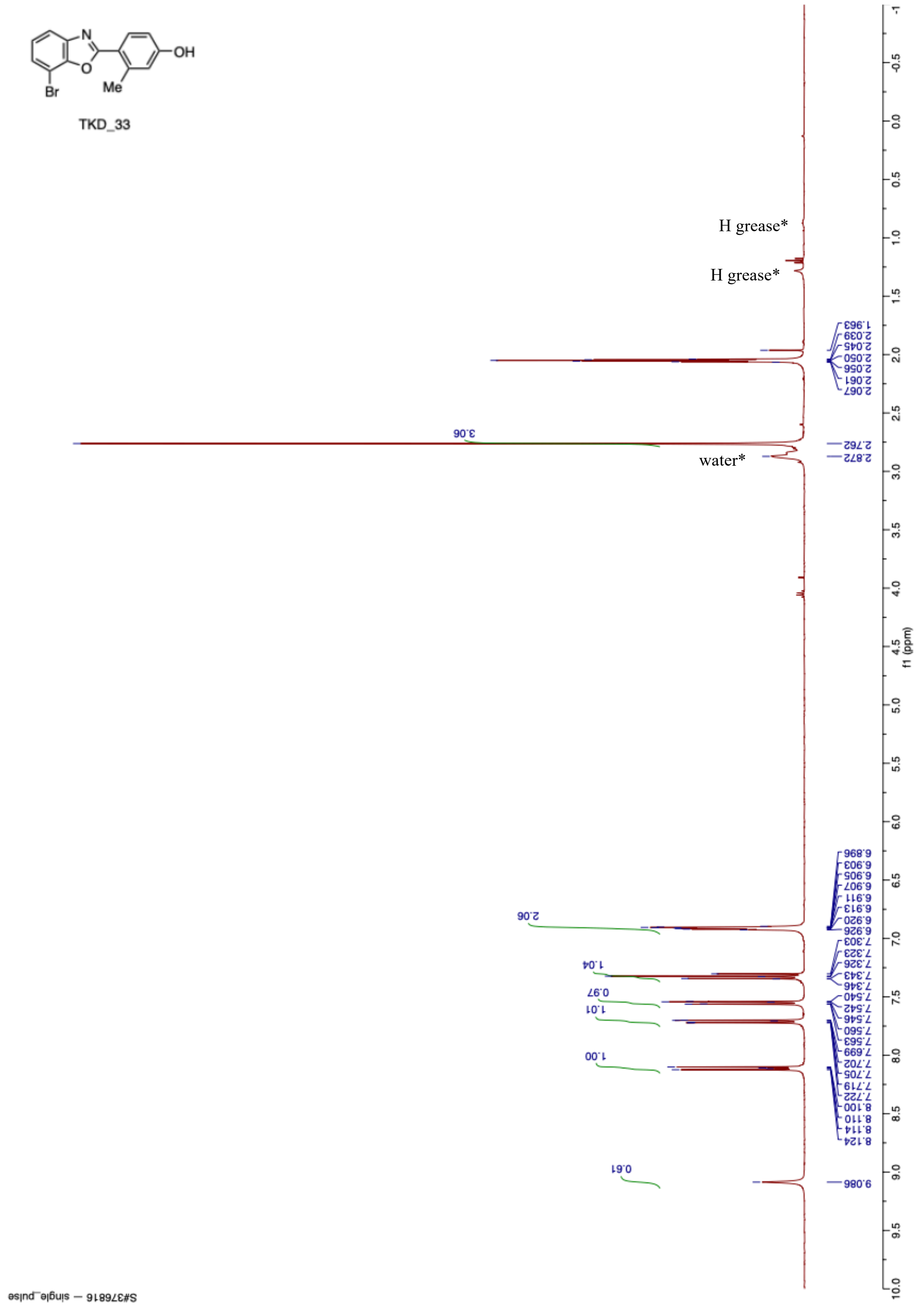

^{13}C NMR of **TKD33** (101 MHz, acetone-d_6)

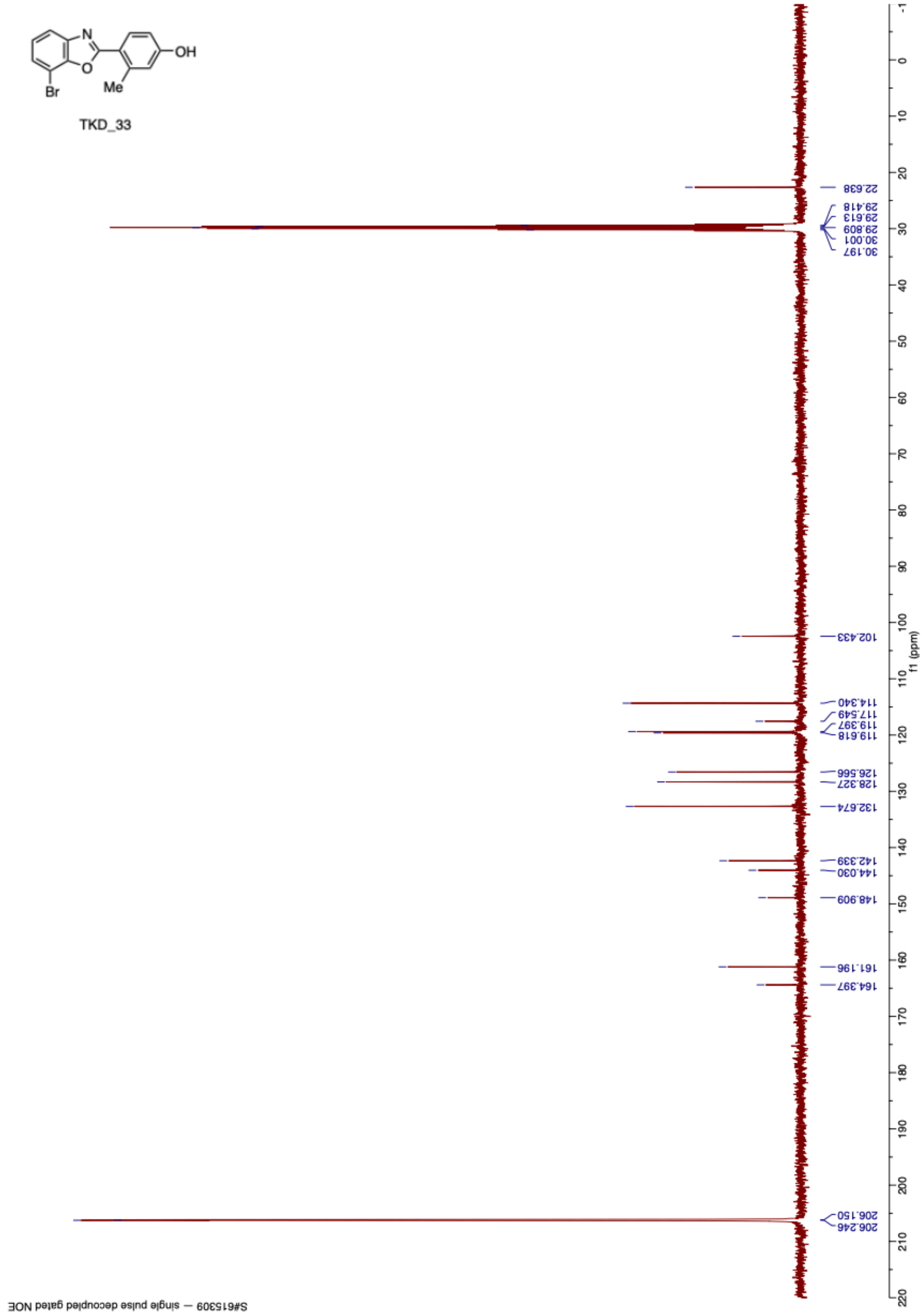

^{1}H NMR of **TKD35** (600 MHz, acetone-d_6)

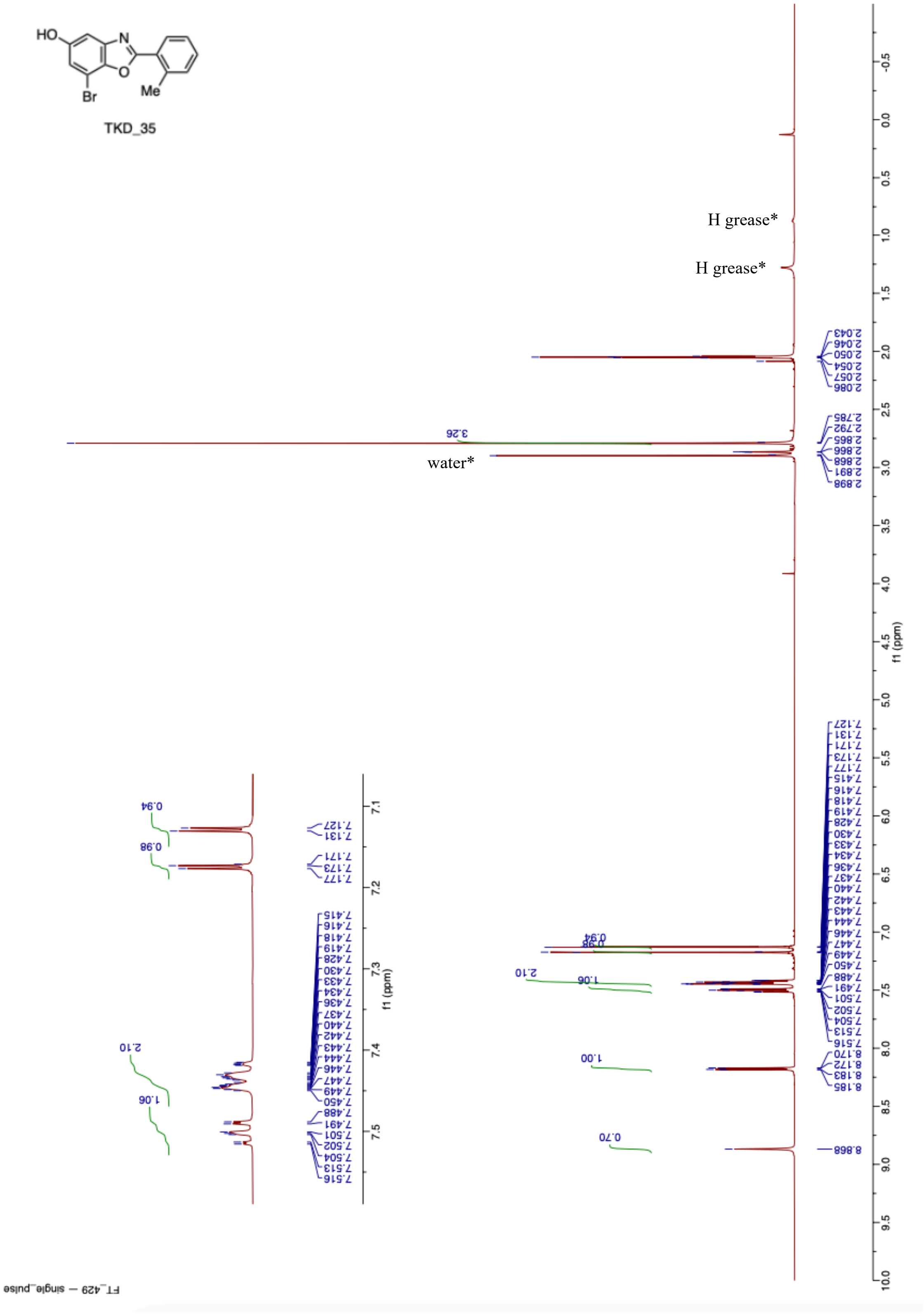

^{13}C NMR of **TKD35** (151 MHz, acetone-d_6)

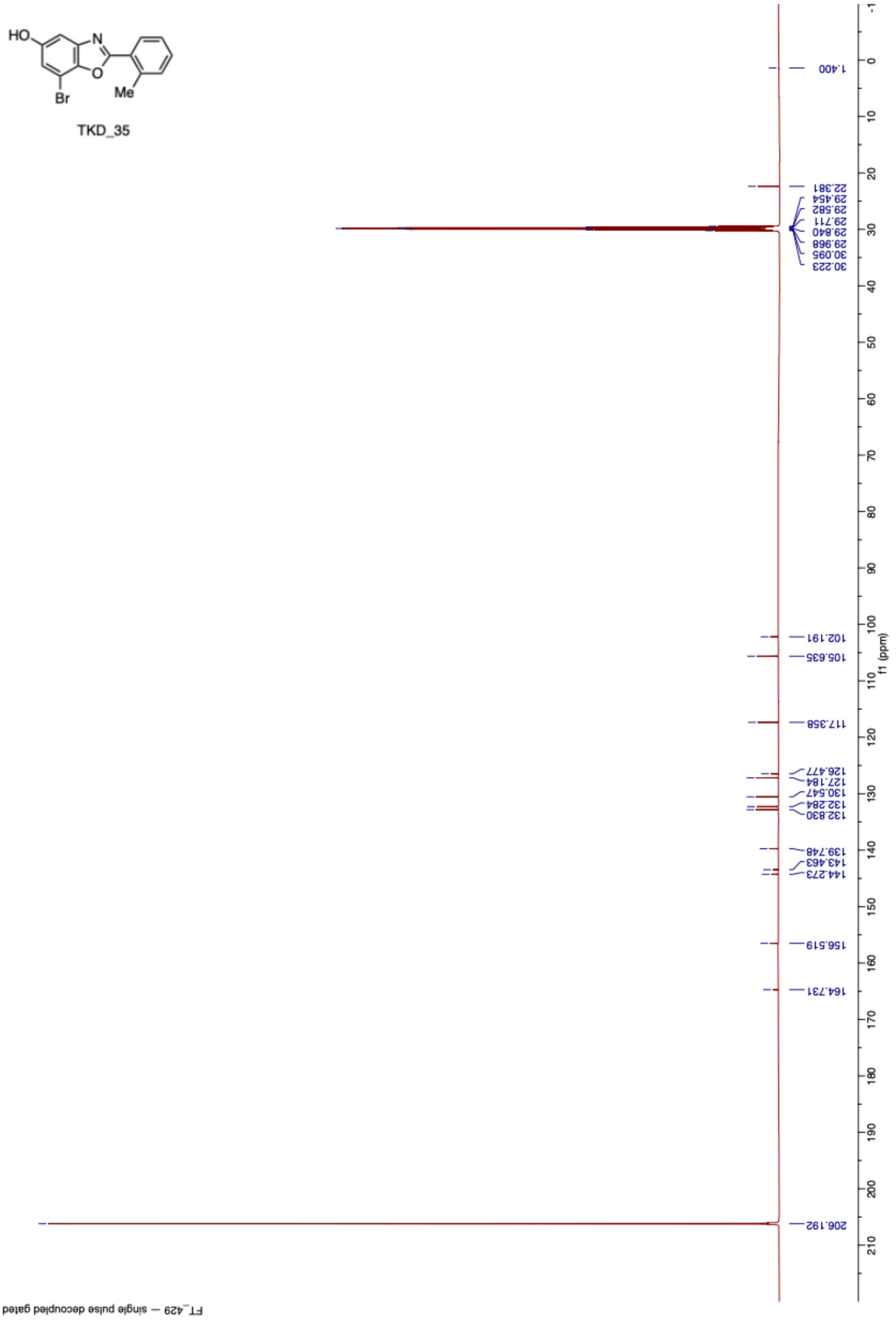

^{1}H NMR of **TKD25** (400 MHz, acetone-d_6)

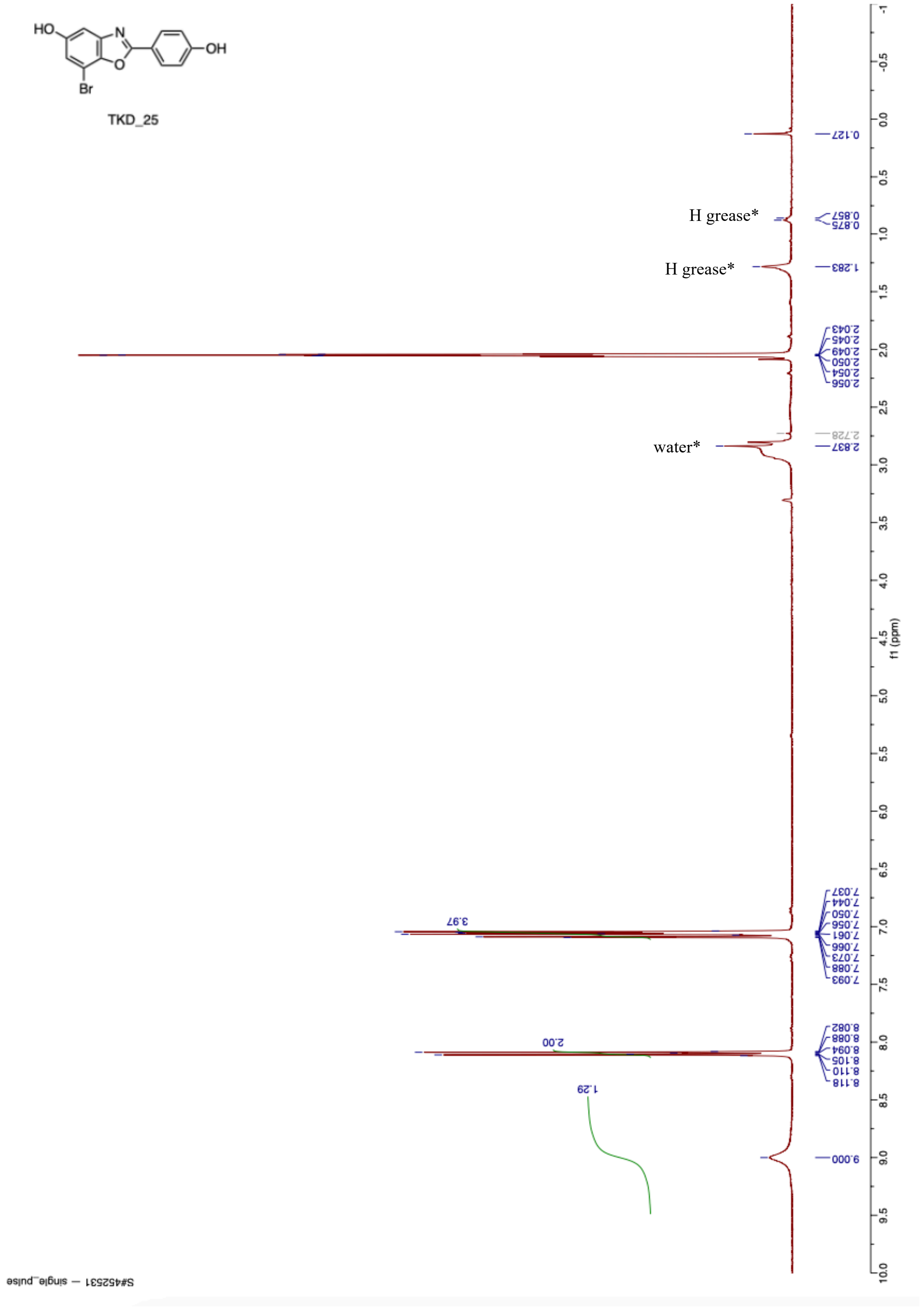

^{13}C NMR of **TKD25** (101 MHz, acetone-d_6)

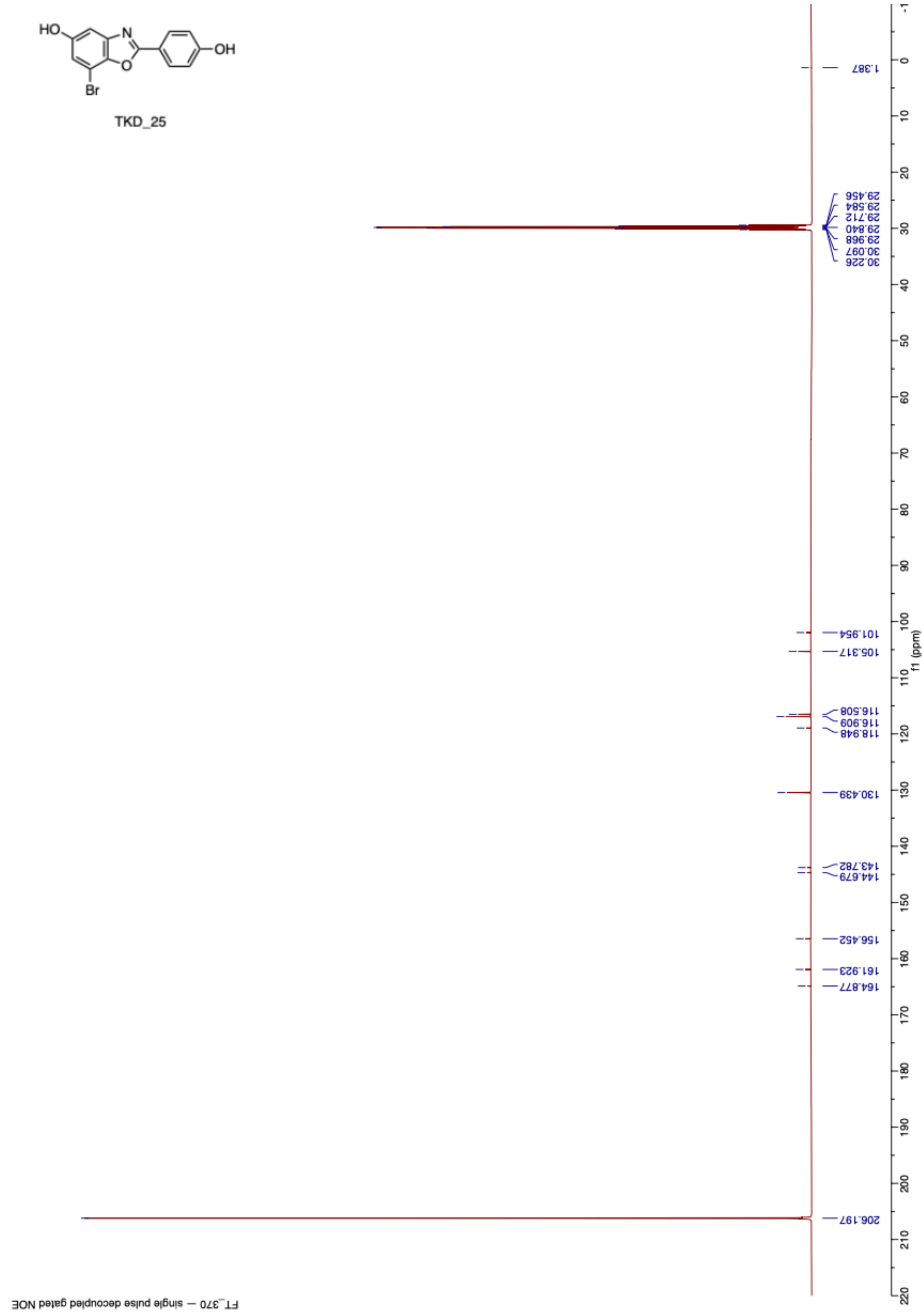

^{1}H NMR of **TKD26** (600 MHz, acetone-d_6)

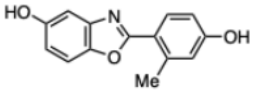

TKD_26

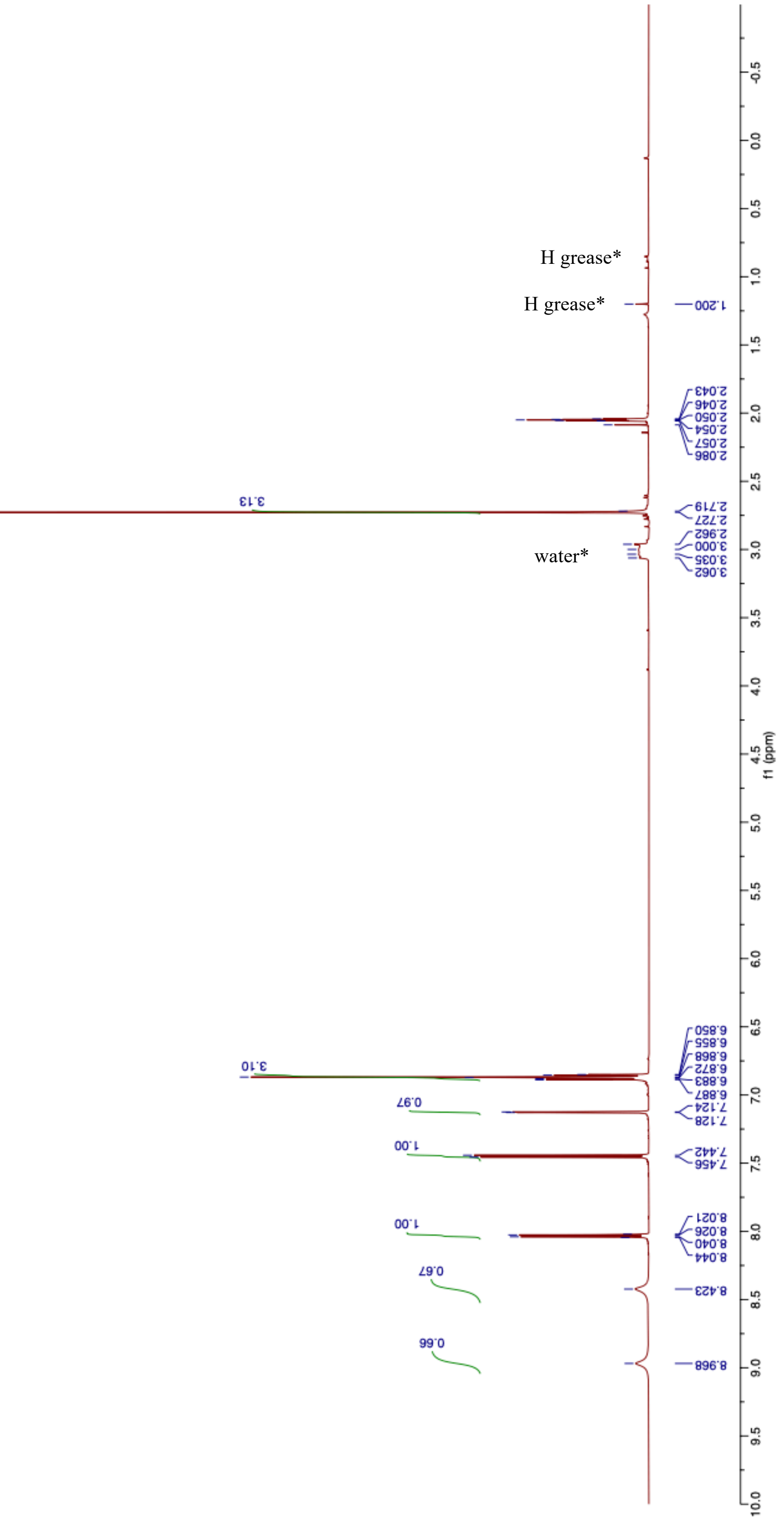

^{13}C NMR of **TKD26** (151 MHz, acetone-d_6)

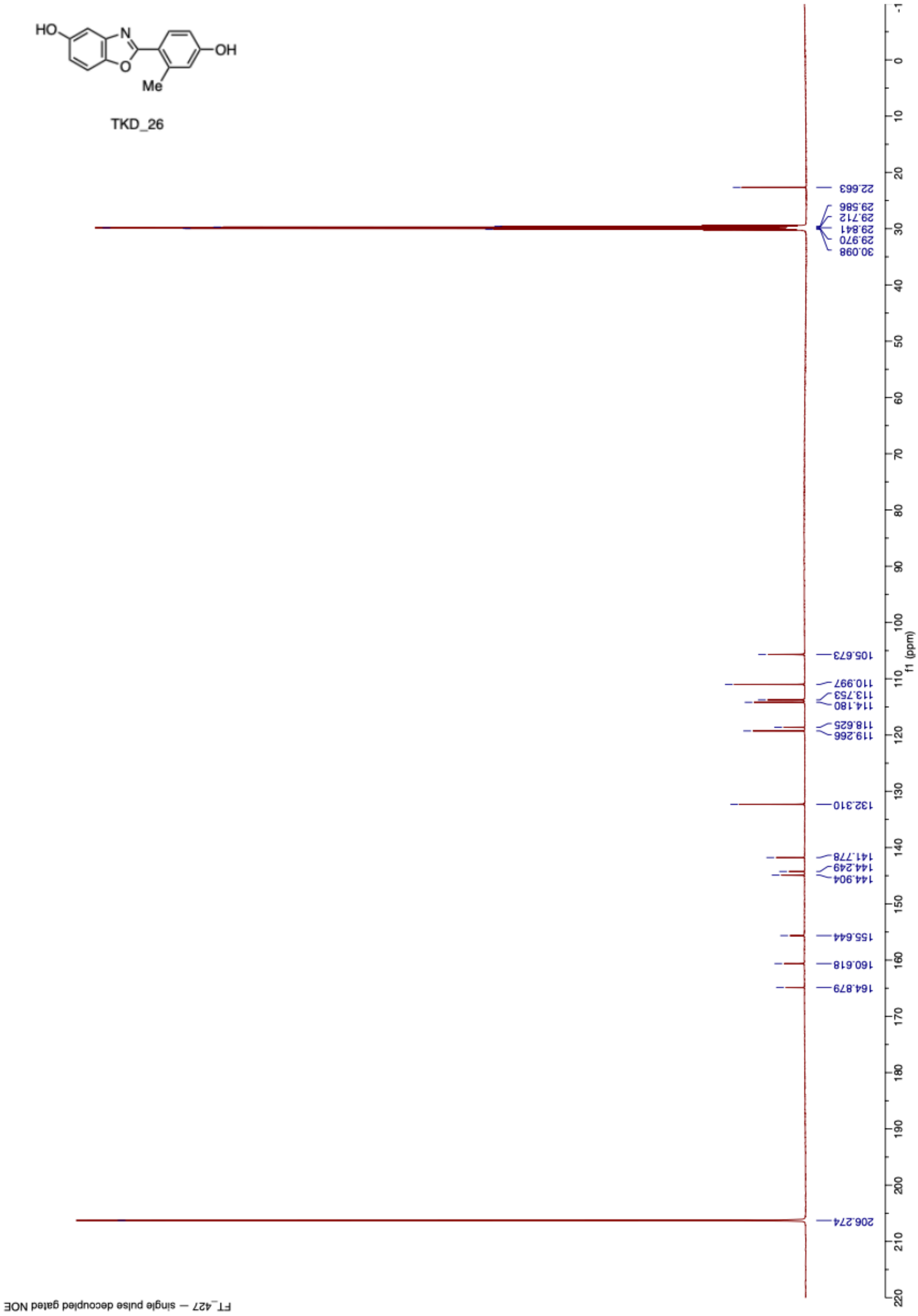

^{1}H NMR of **TKD10** (400 MHz, acetone-d_6)

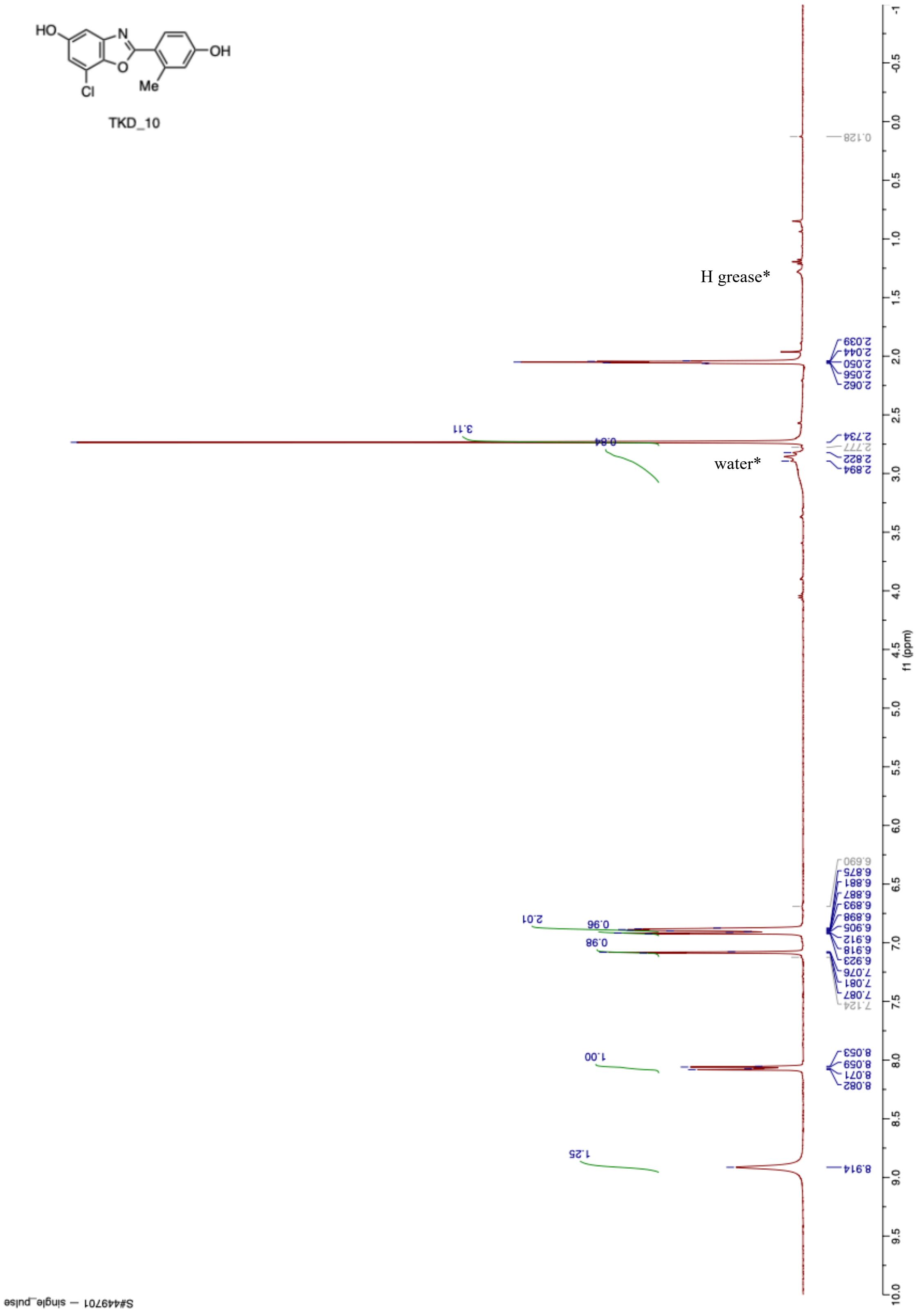

^{13}C NMR of **TKD10** (101 MHz, acetone-d_6)

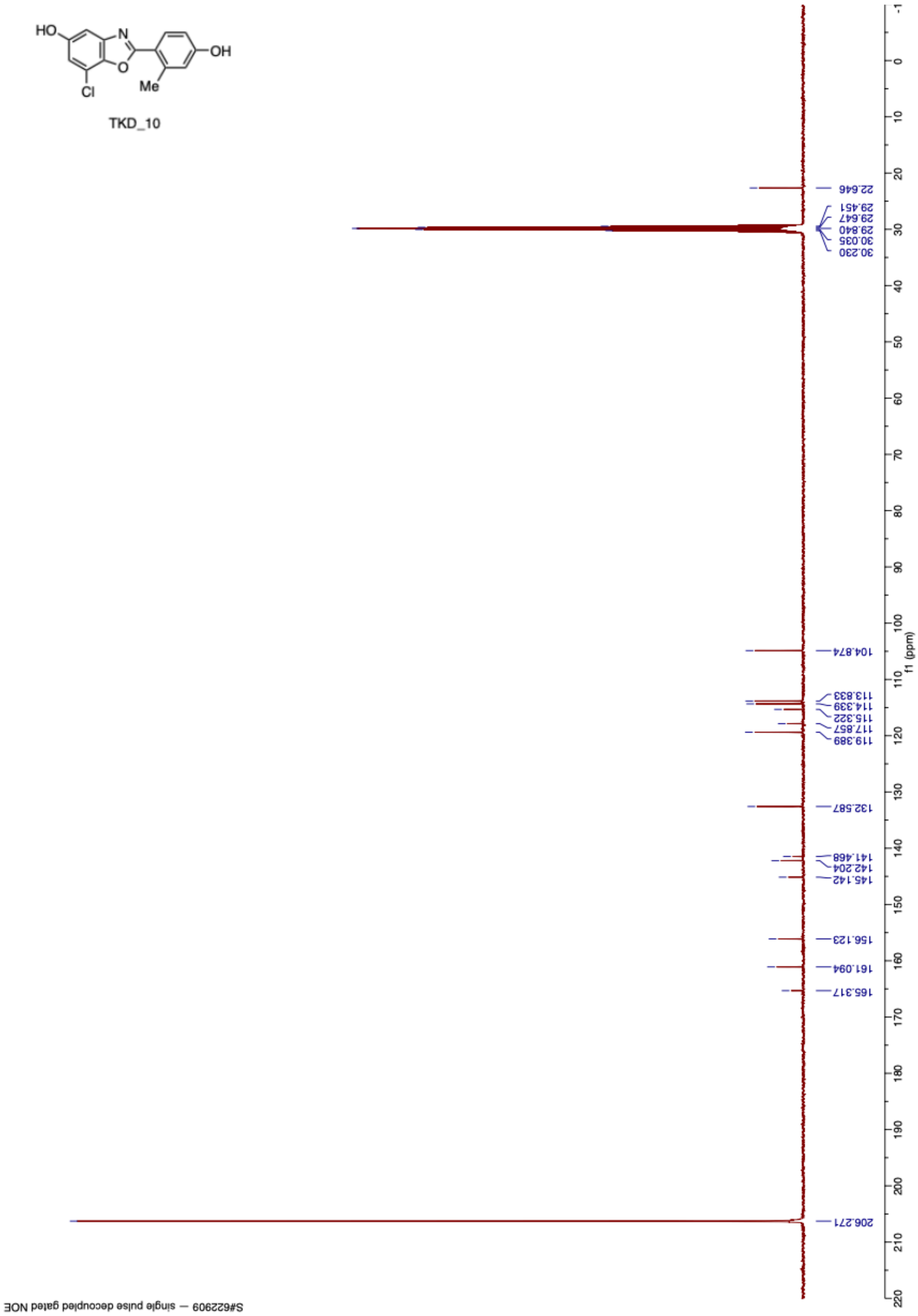

^{1}H NMR of **TKD125** (400 MHz, acetone-d_6)

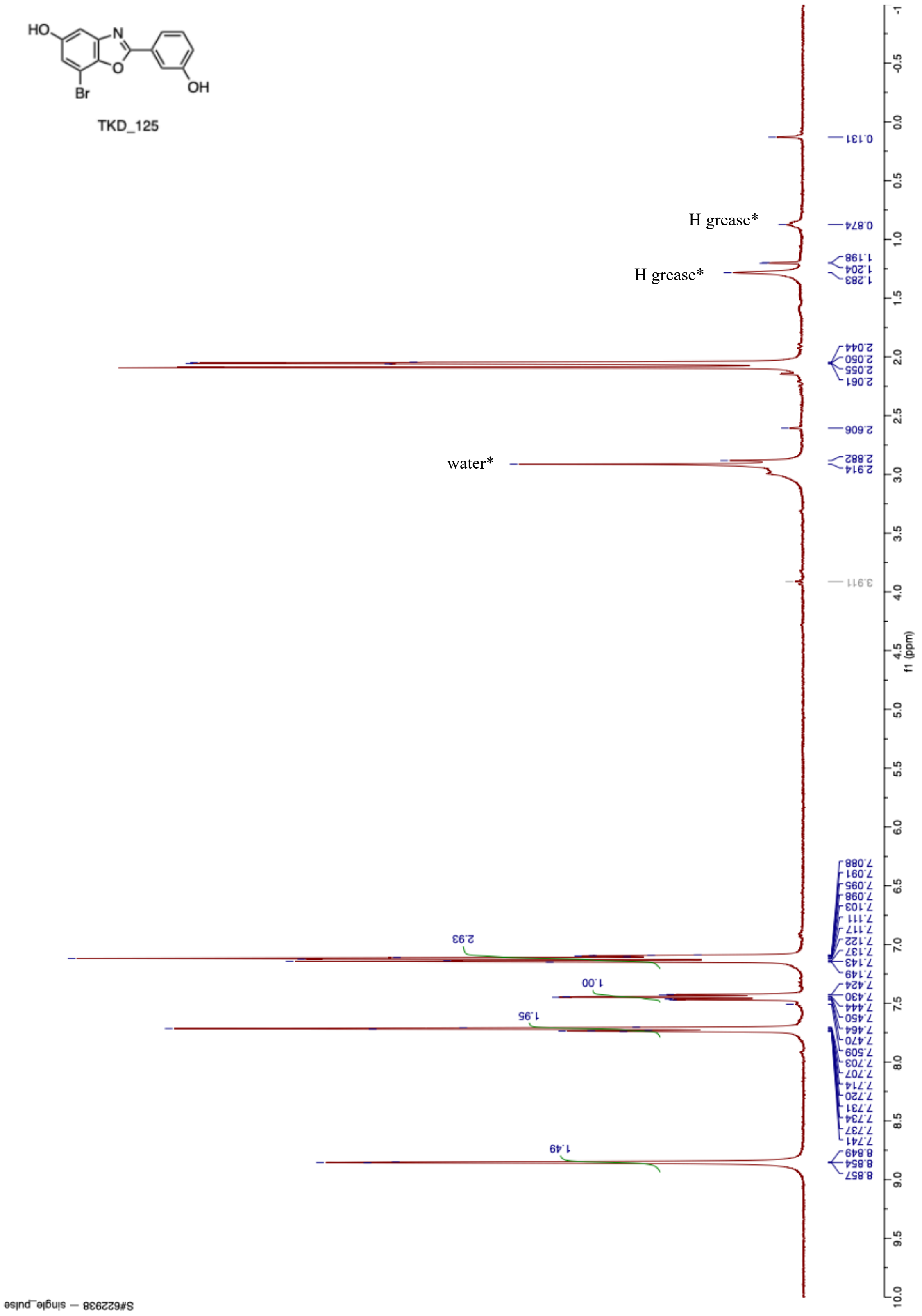

^{13}C NMR of **TKD125** (101 MHz, acetone-d_6)

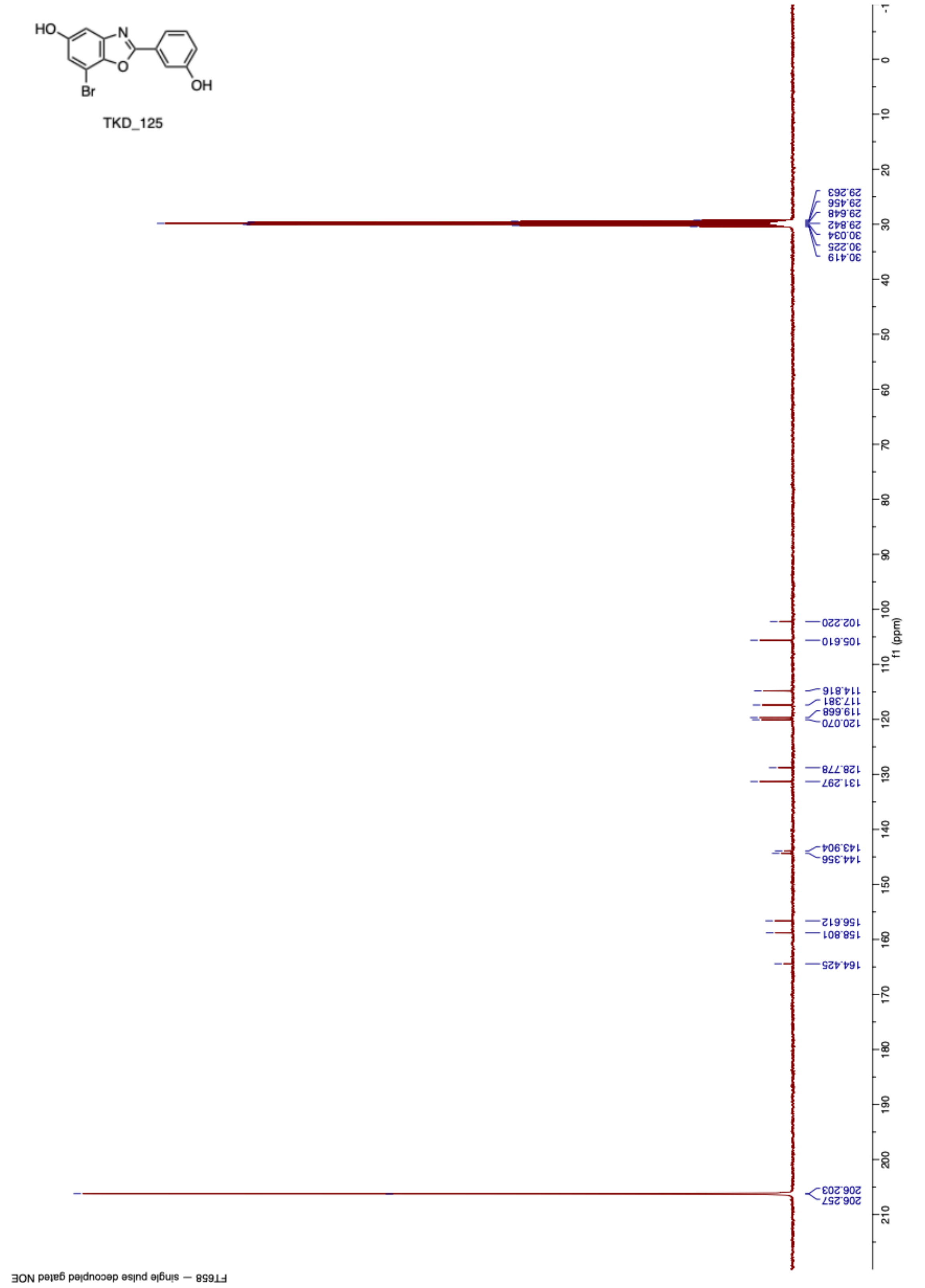

^{1}H NMR of **TKD149** (400 MHz, acetone-d_6)

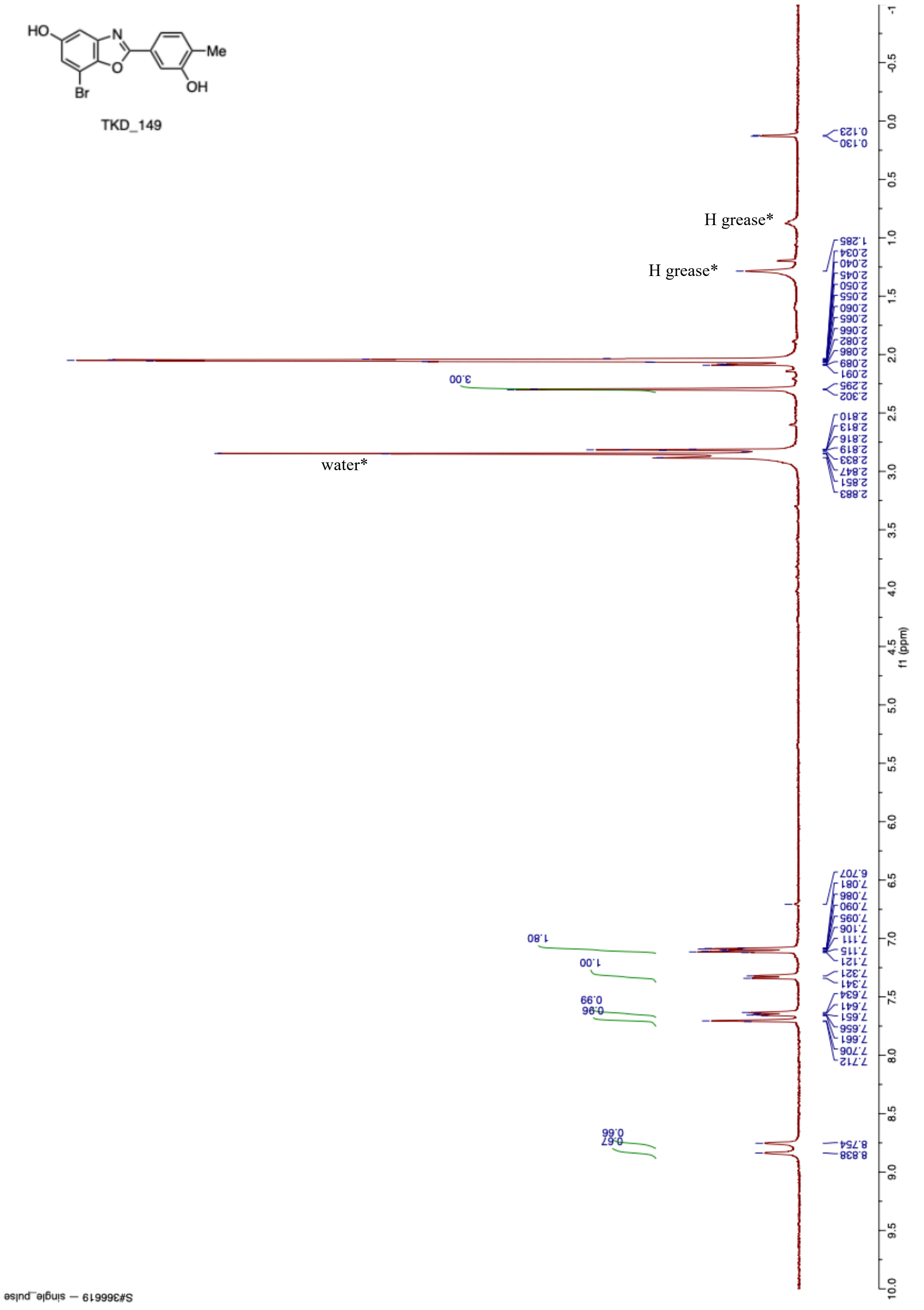

^{13}C NMR of **TKD149** (101 MHz, acetone-d_6)

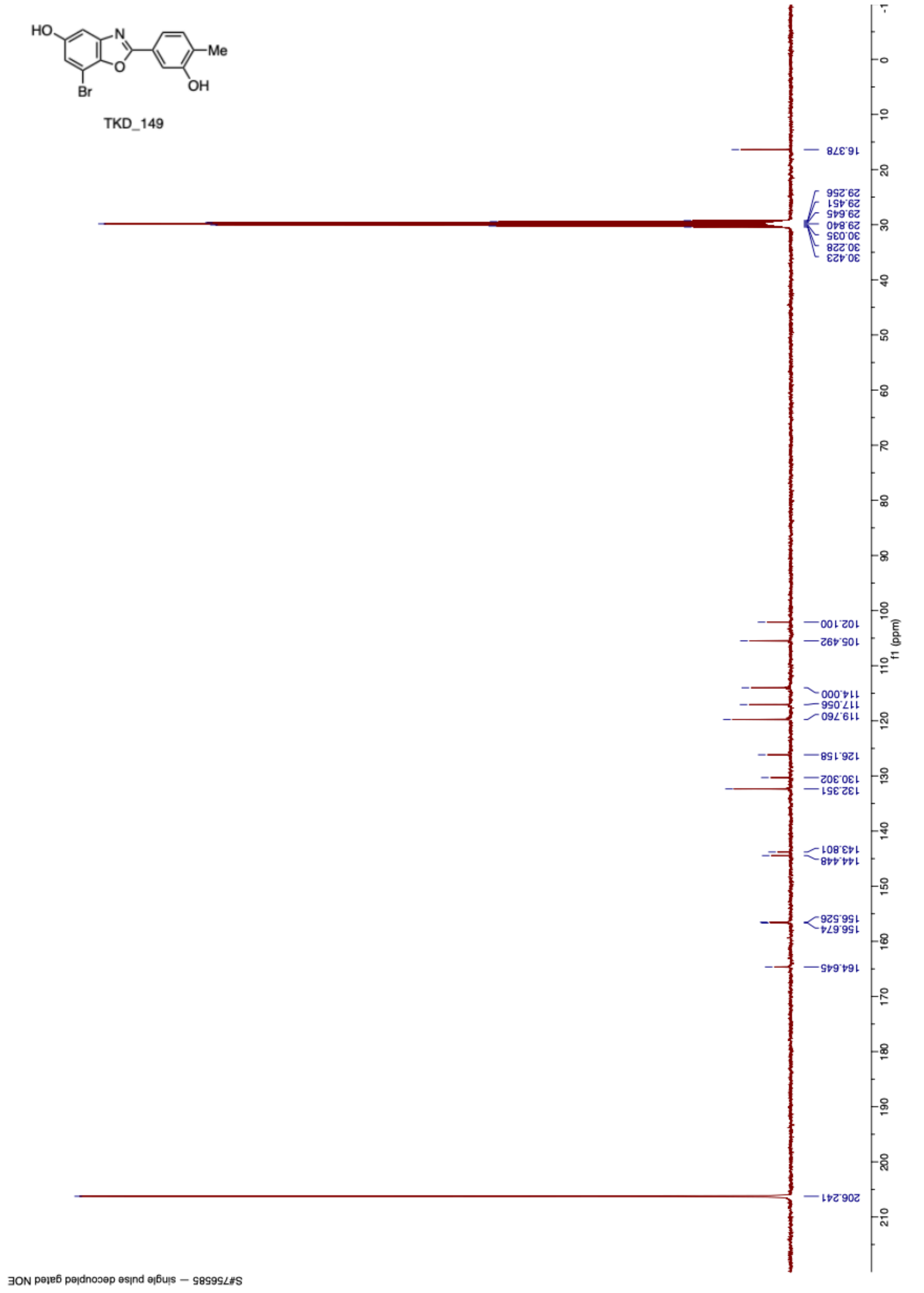

^{1}H NMR of **TKD66** (400 MHz, acetone-d_6)

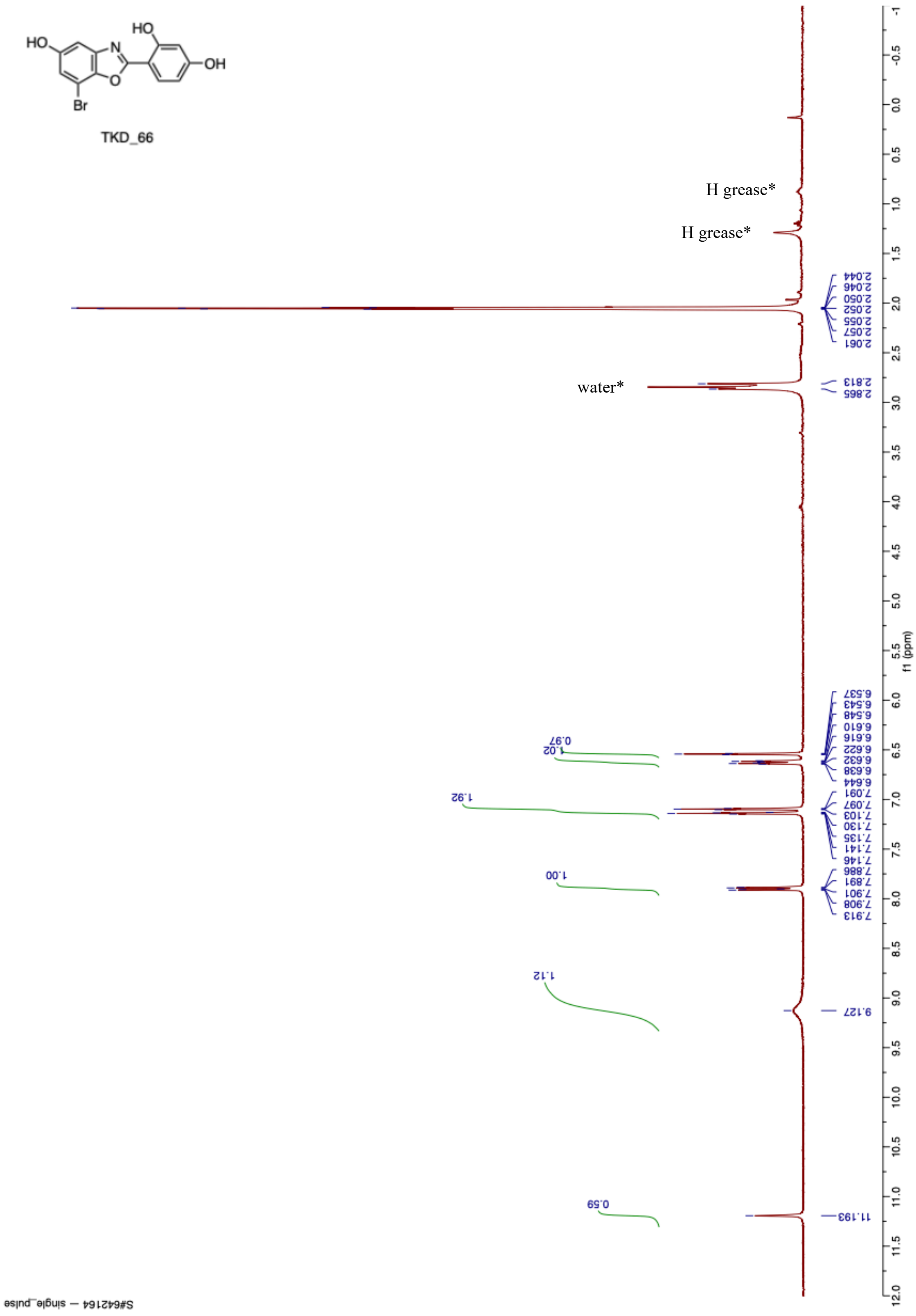

^{13}C NMR of **TKD66** (101 MHz, acetone-d_6)

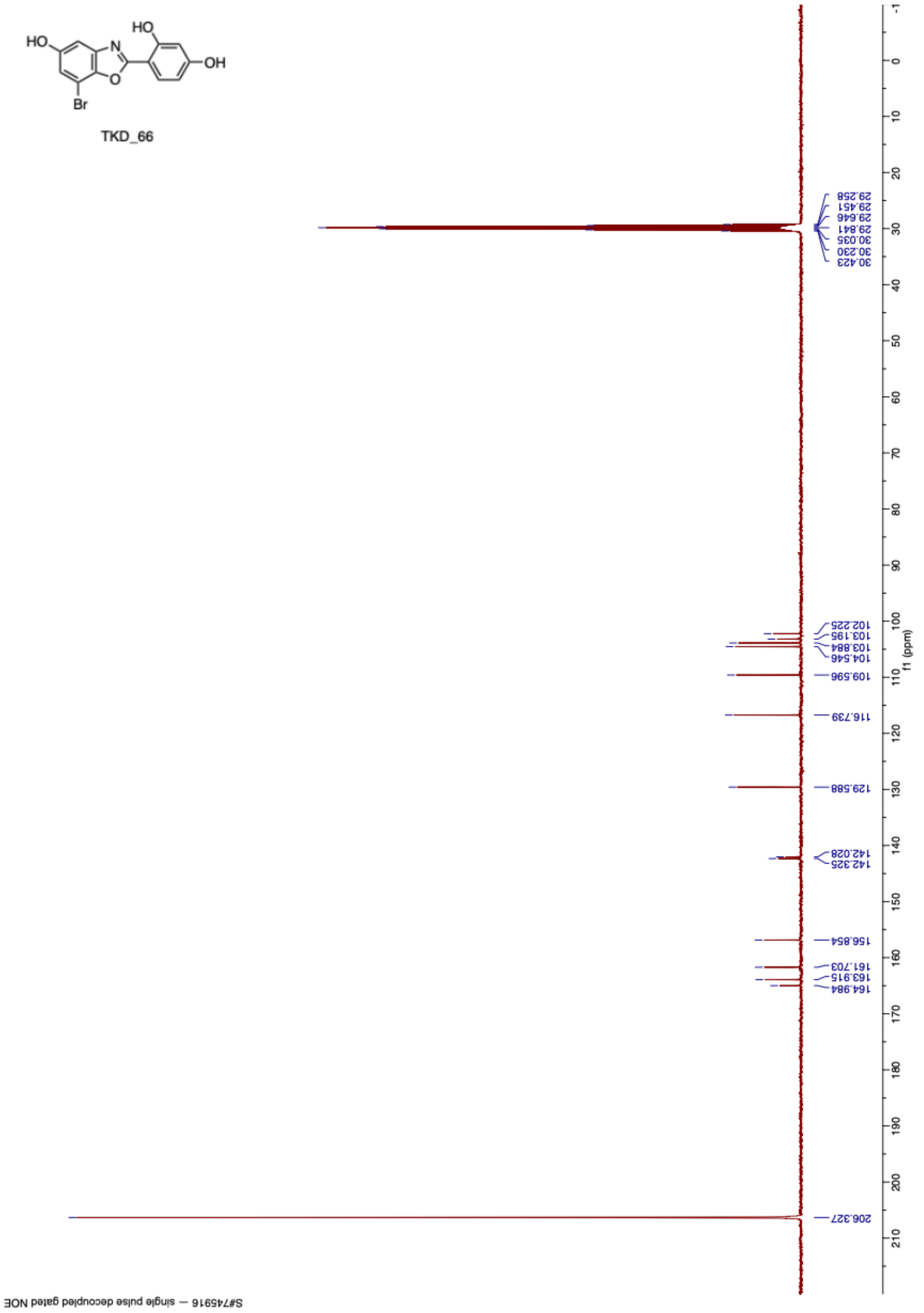

^{1}H NMR of **TKD147** (400 MHz, acetone-d_6)

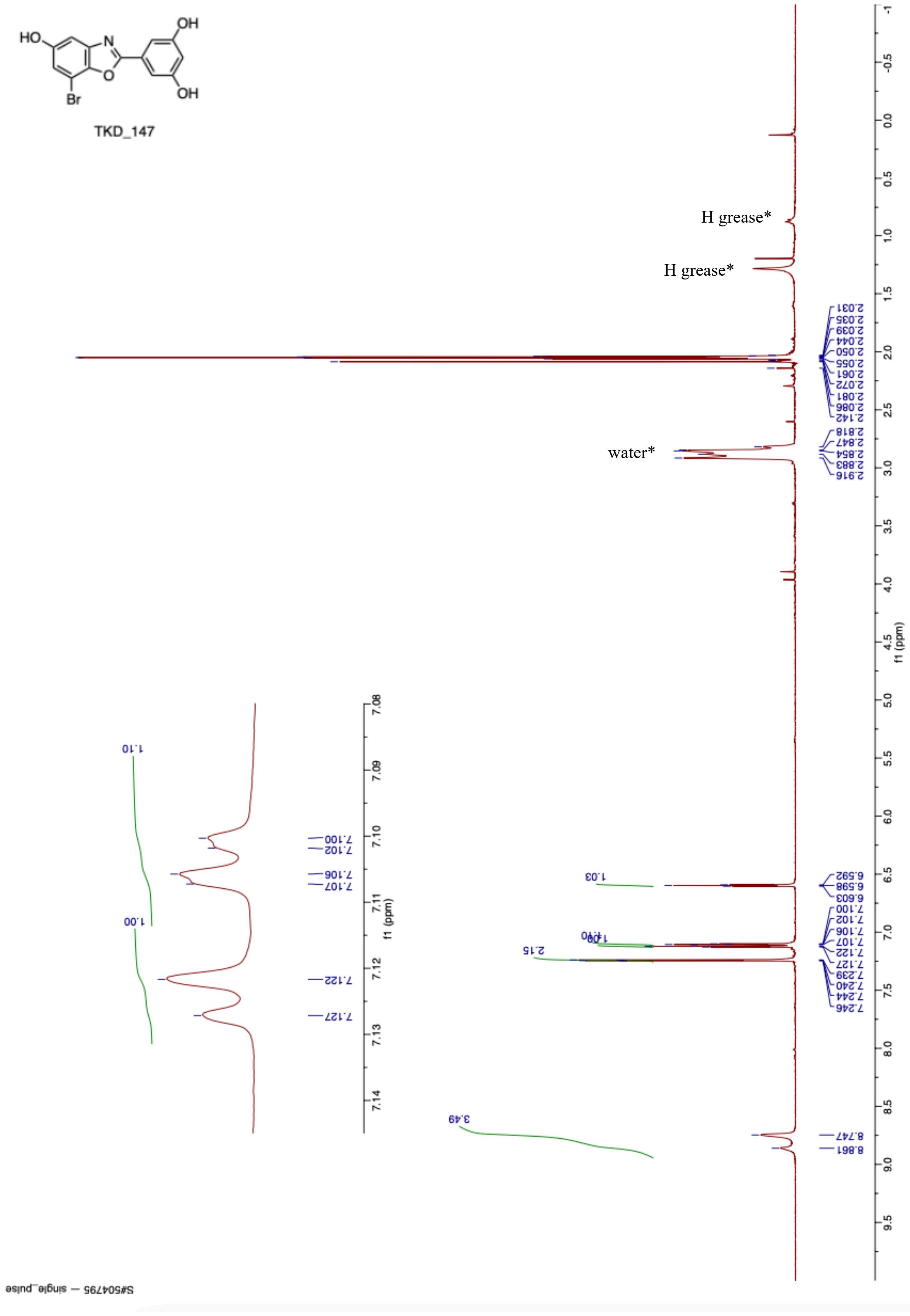

^{13}C NMR of **TKD147** (101 MHz, acetone-d_6)

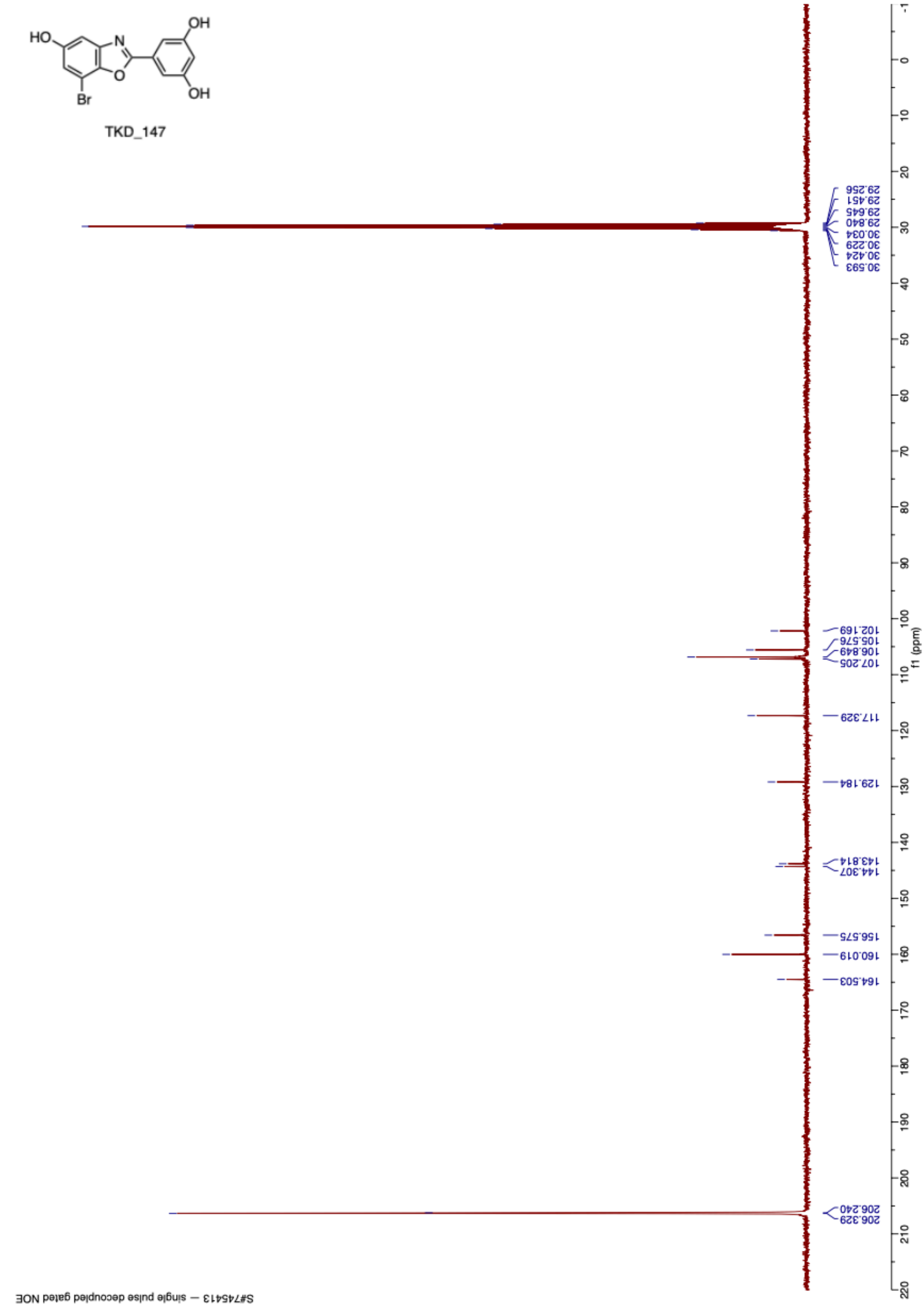

^{1}H NMR of **TKD129** (400 MHz, acetone-d_6)

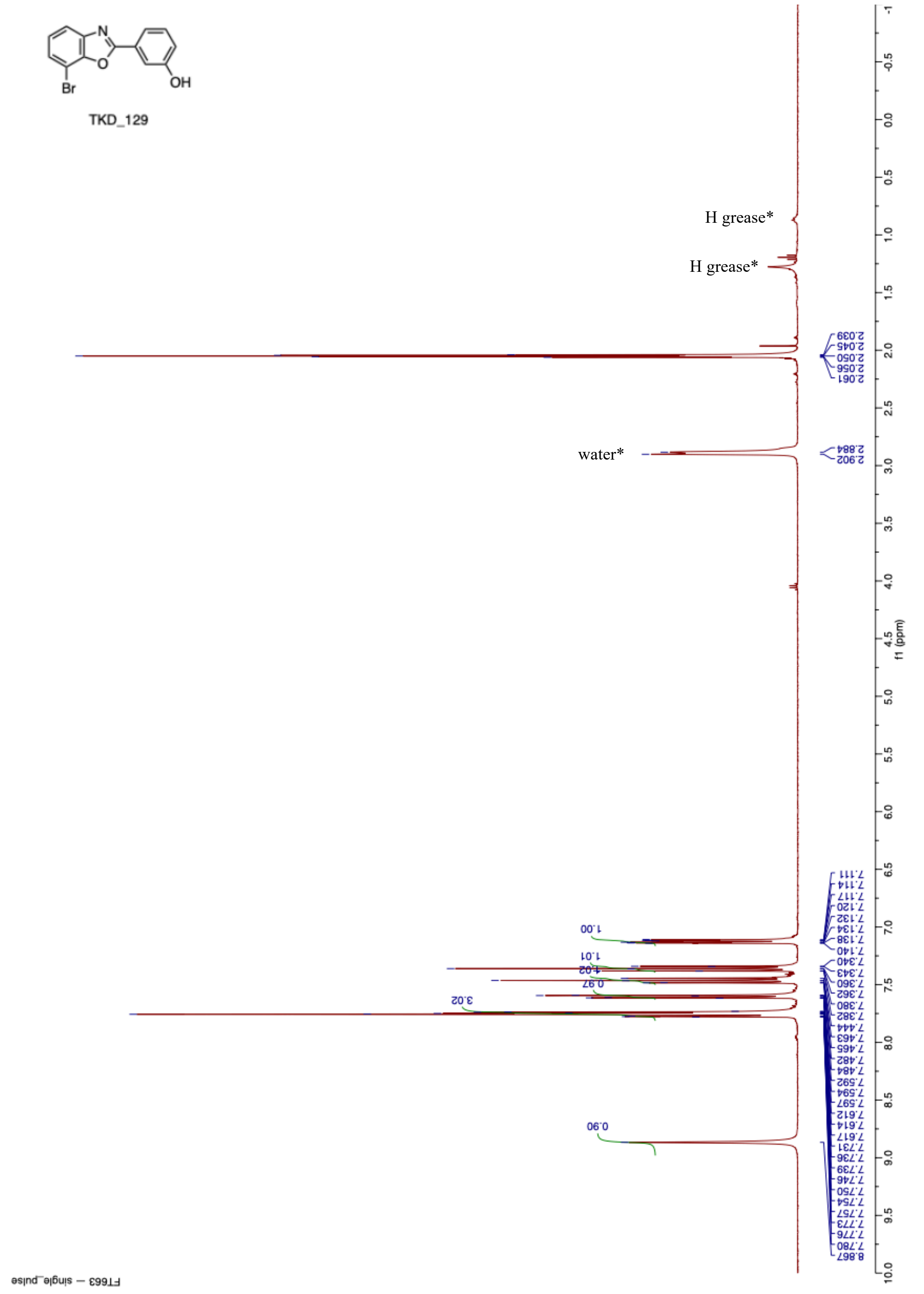

^{13}C NMR of **TKD129** (101 MHz, acetone-d_6)

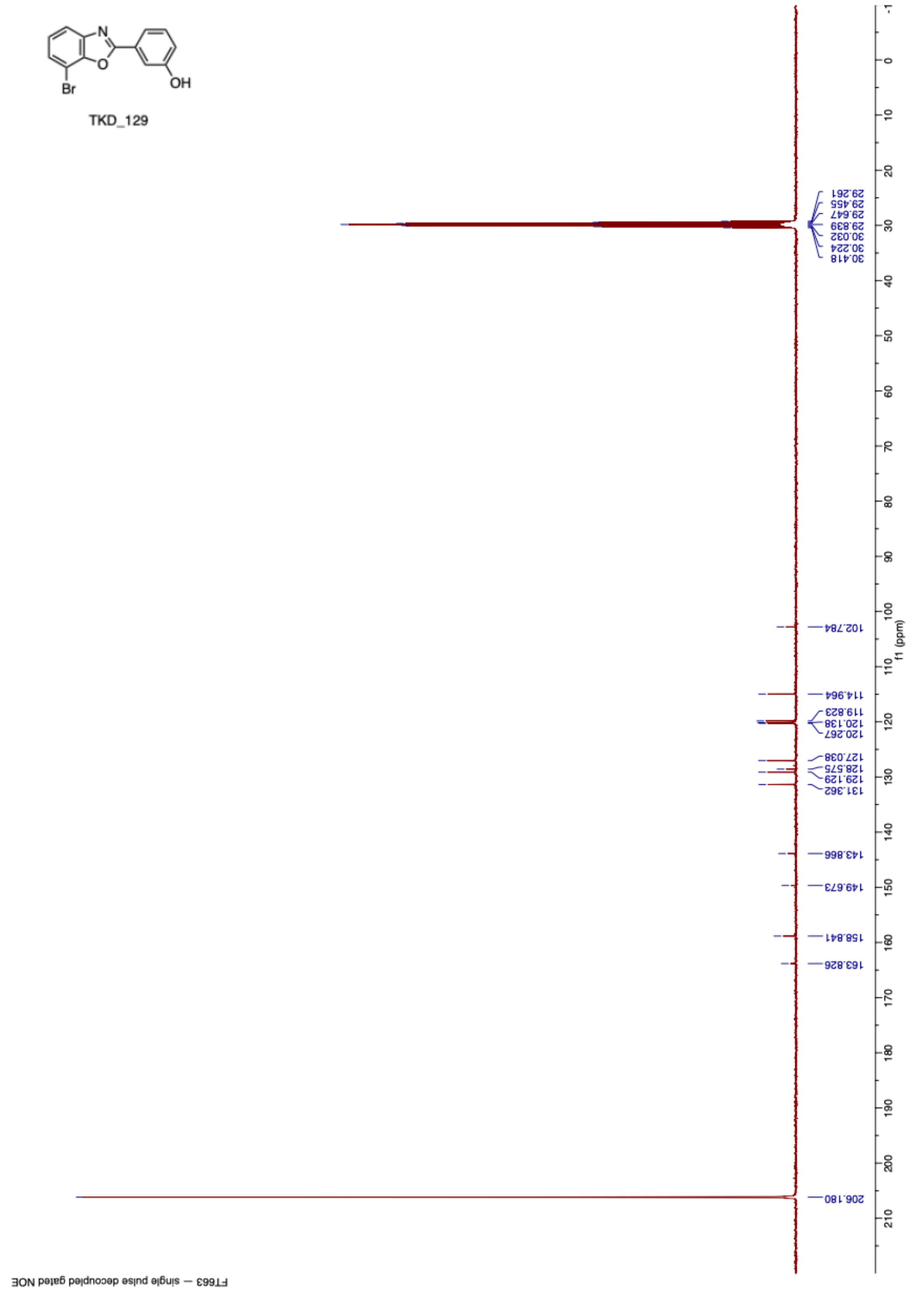

^{1}H NMR of **TKD100** (400 MHz, acetone-d_6)

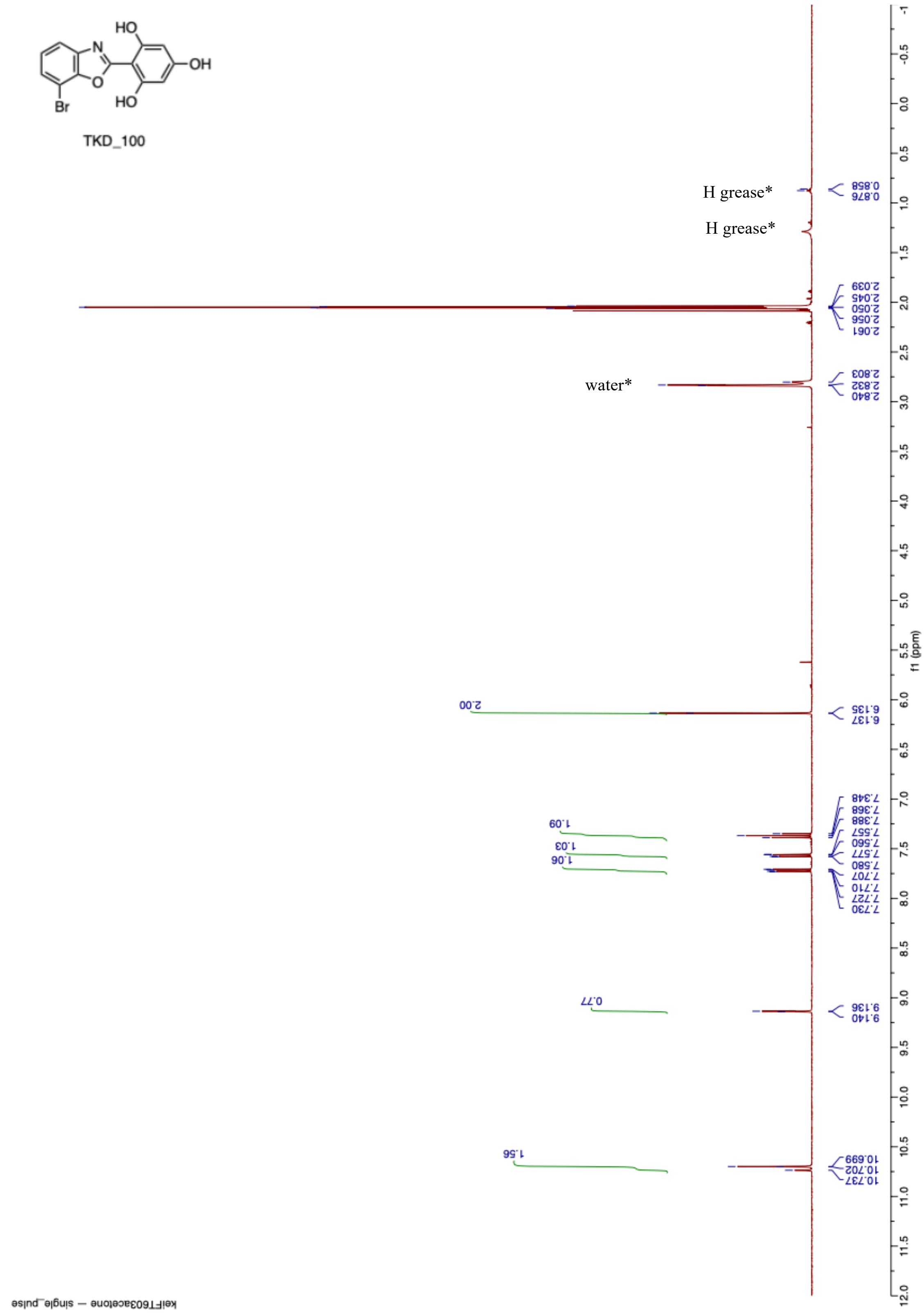

^{13}C NMR of **TKD100** (101 MHz, acetone-d_6)

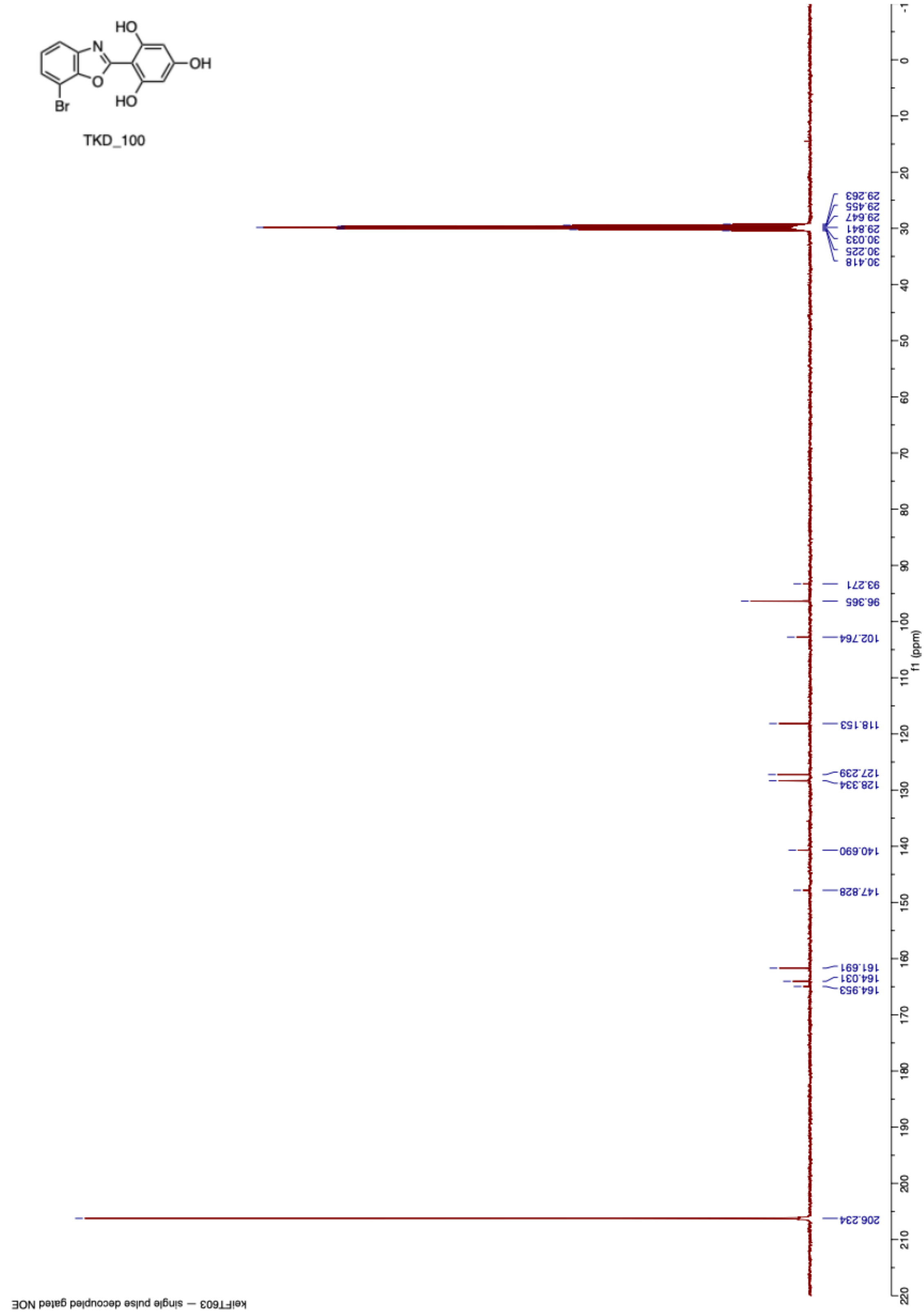

^{1}H NMR of **TKD146** (400 MHz, acetone-d_6)

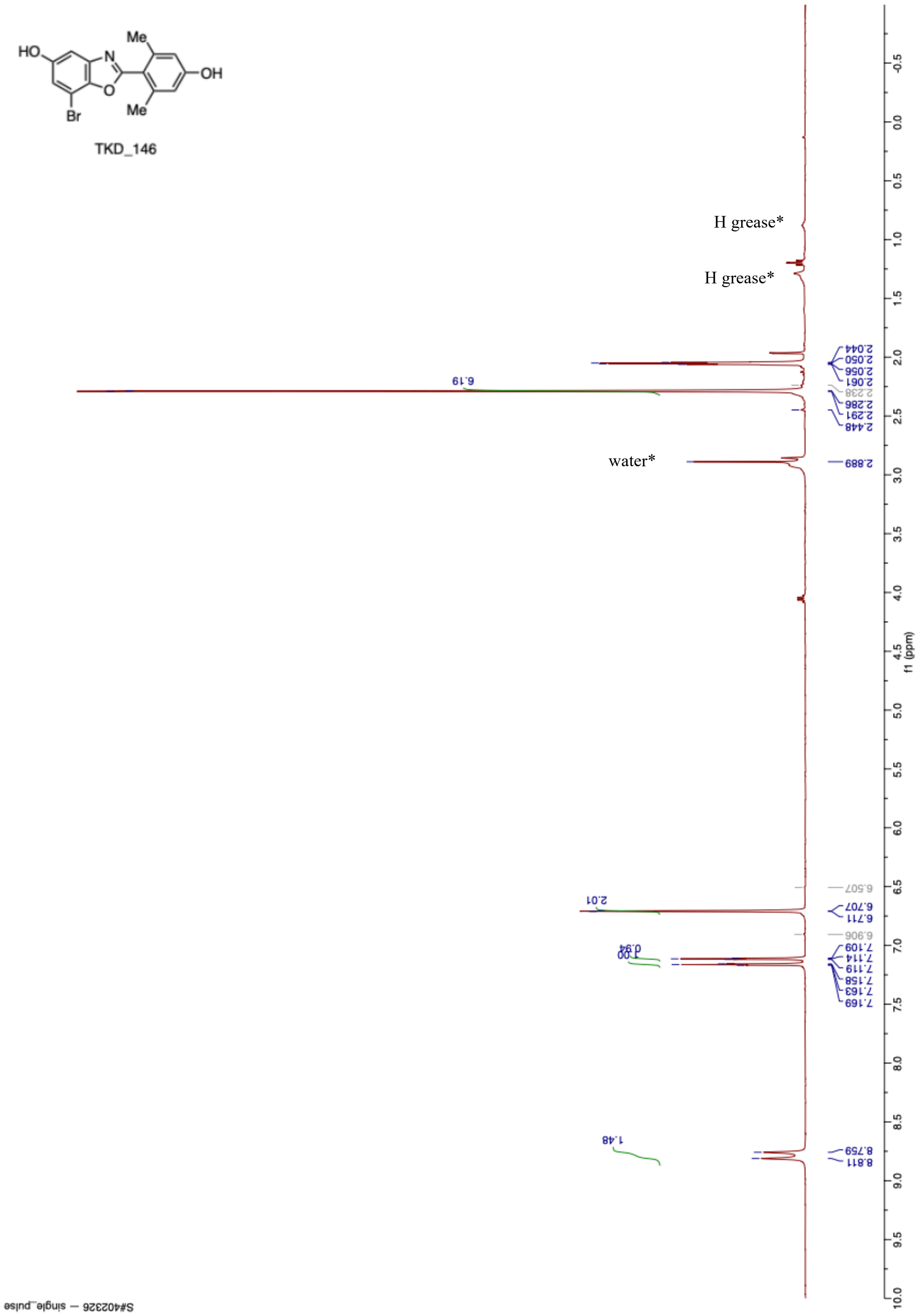

^{13}C NMR of **TKD146** (101 MHz, acetone-d_6)

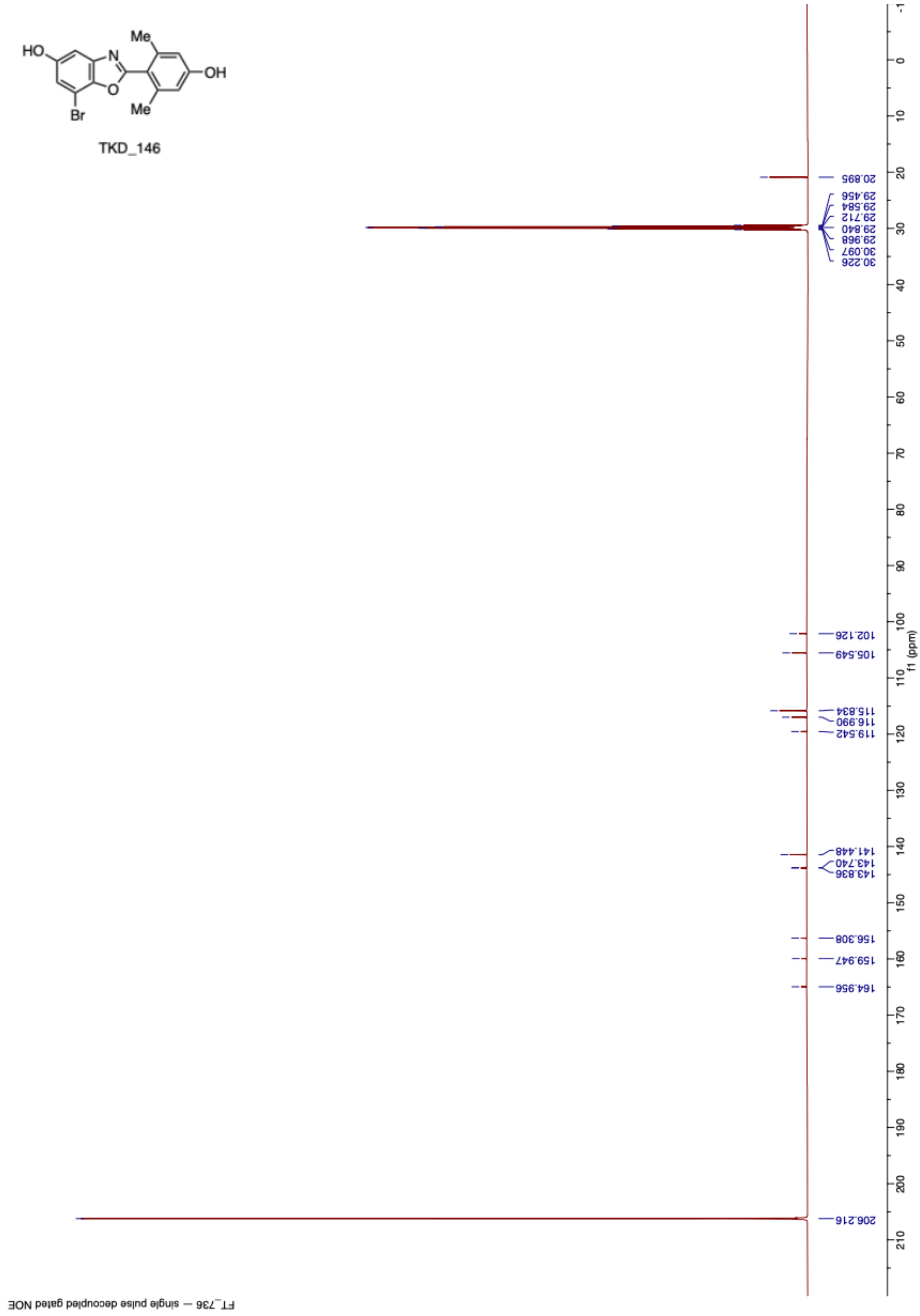

^{1}H NMR of **TKD152** (400 MHz, acetone-d_6)

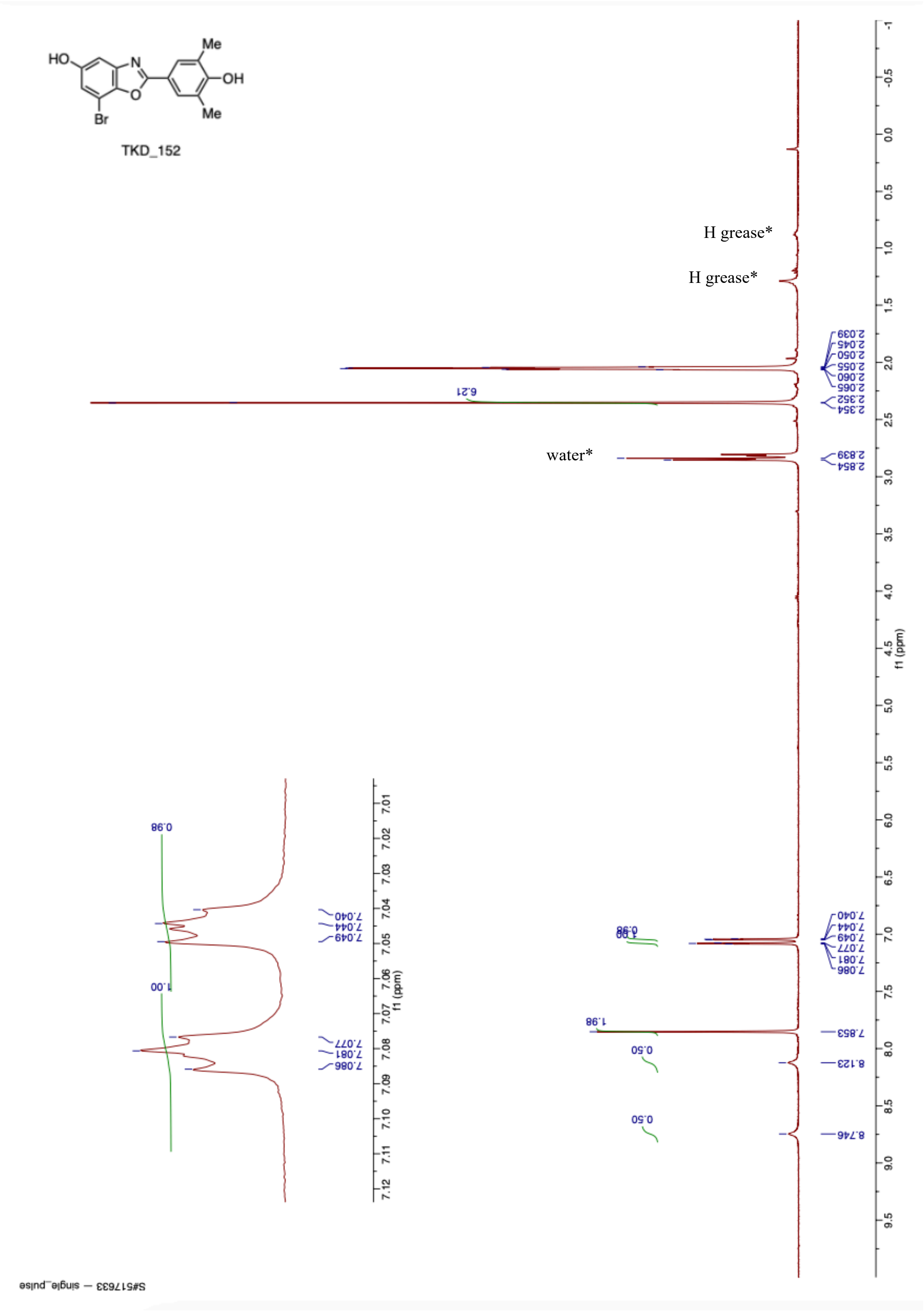

^{13}C NMR of **TKD152** (101 MHz, acetone-d_6)

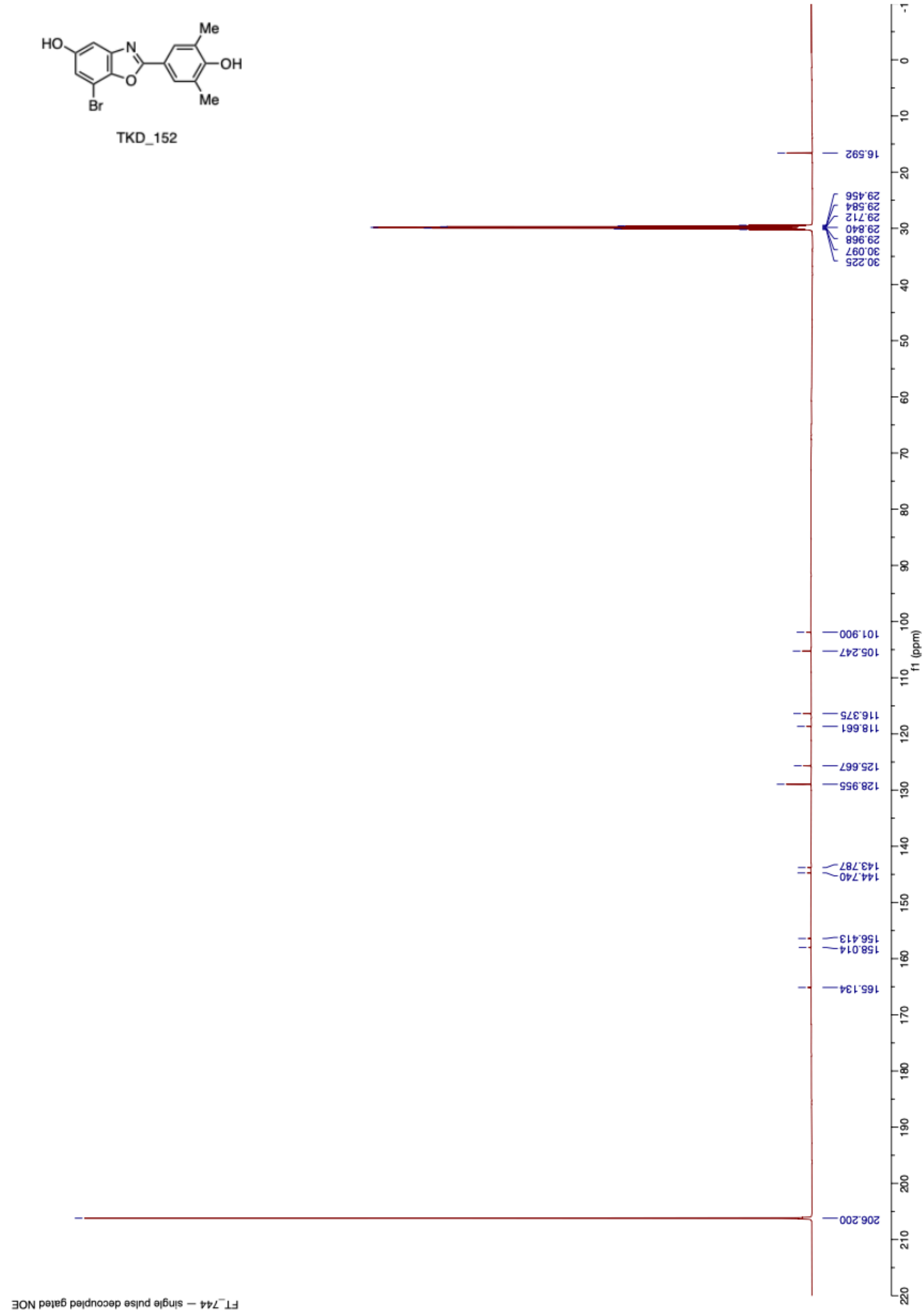

^{1}H NMR of **TKD150** (400 MHz, acetone-d_6)

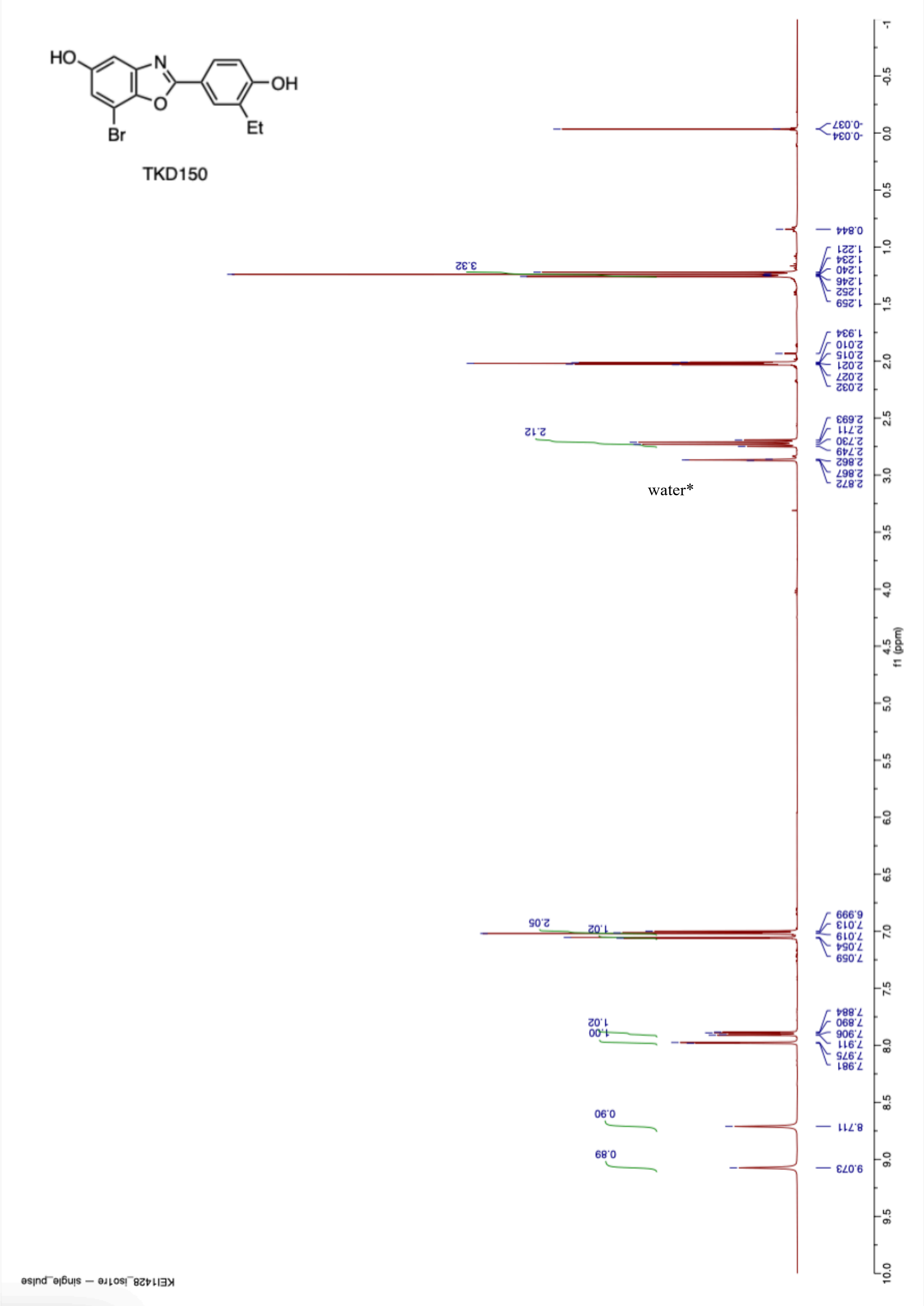

^{13}C NMR of **TKD150** (101 MHz, acetone-d_6)

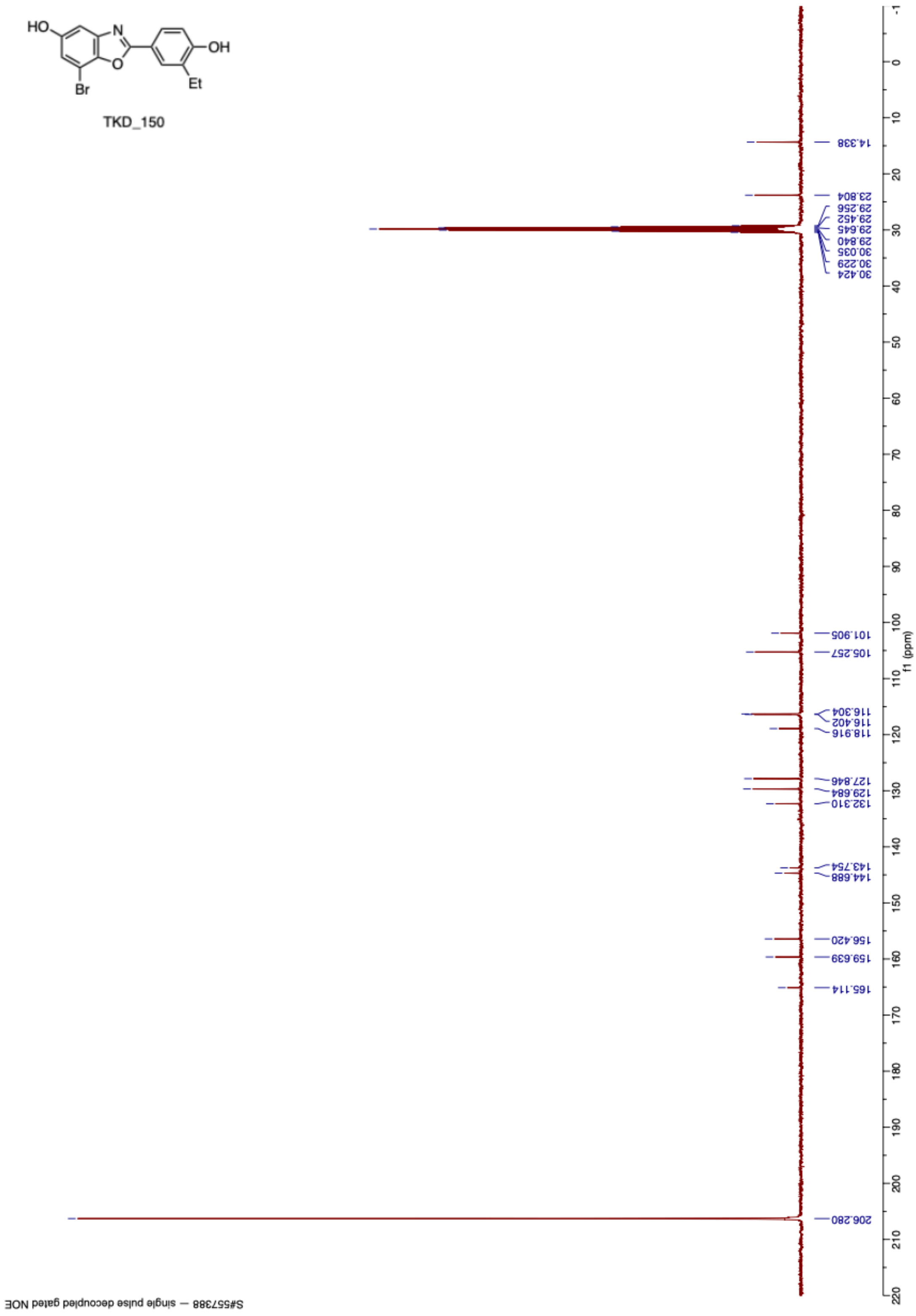

9. HPLC Chromatogram

PA86 purity: 96.3%

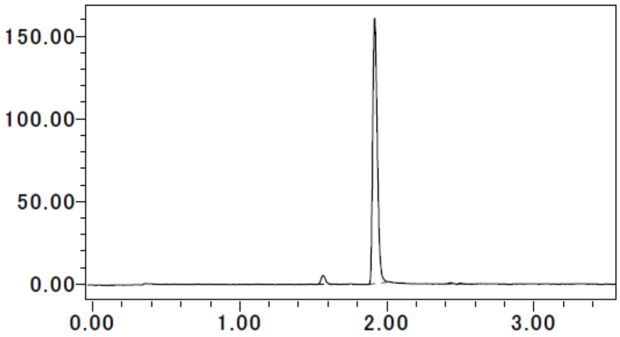

Result of Analysis

	Retention Time (min)	Peak area (μV×sec)	Divisional method	% of Area
1	1.566	11298	BB	3.15
2	1.917	345192	BB	96.25
3	2.435	1391	bb	0.39
4	2.502	760	bb	0.21

TKD1 purity: 90.6%

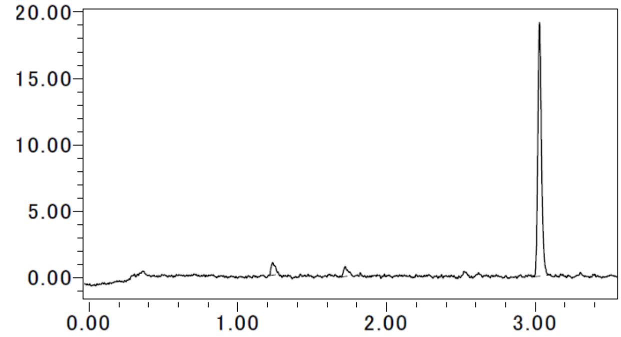

Result of Analysis

	Retention Time (min)	Peak area (μV×sec)	Divisional method	% of Area
1	1.234	1871	bb	5.16
2	1.722	1538	bb	4.24
3	3.029	32856	BB	90.60

TKD33 purity: 99.1%

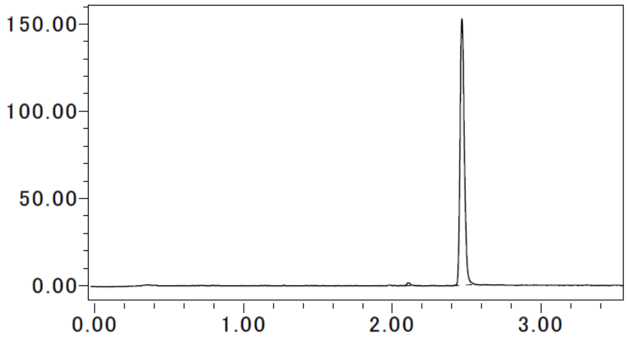

Result of Analysis

	Retention Time (min)	Peak area (μV×sec)	Divisional method	% of Area
1	2.107	2761	Bb	0.89
2	2.468	306900	BB	99.11

TKD35 purity: 99.6%

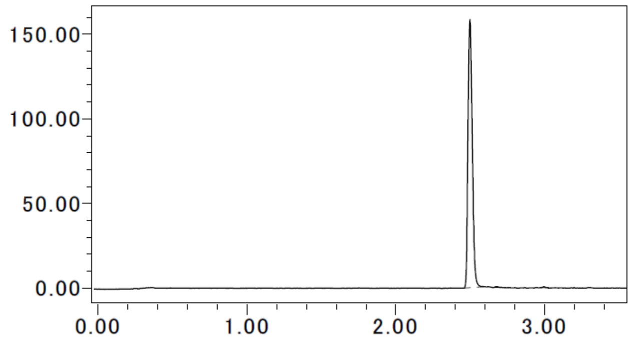

Result of Analysis

	Retention Time (min)	Peak area (μV×sec)	Divisional method	% of Area
1	2.498	314134	BB	99.59
2	2.679	602	bb	0.19
3	2.996	696	bb	0.22

TKD25 purity: 99.6%

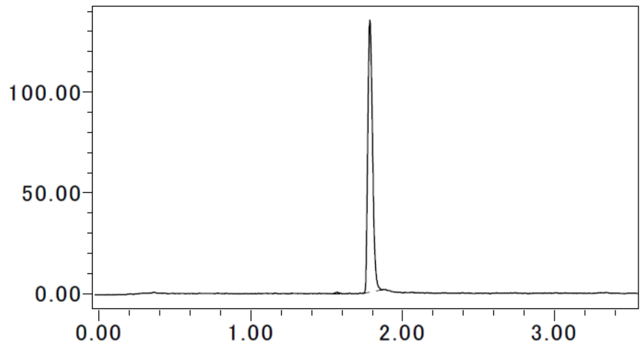

Result of Analysis

	Retention Time (min)	Peak area (μV×sec)	Divisional method	% of Area
1	1.568	1106	bb	0.38
2	1.784	291127	Bb	99.62

TKD26 purity: 99.2%

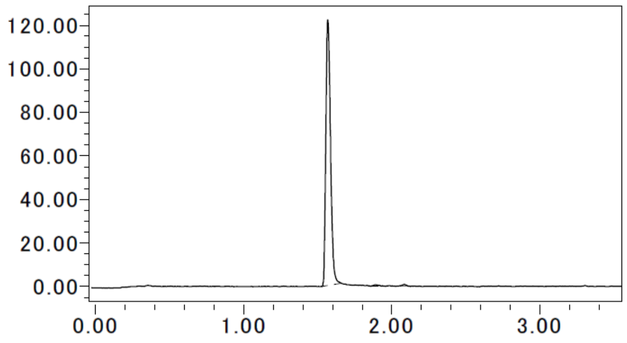

Result of Analysis

	Retention Time (min)	Peak area (μV×sec)	Divisional method	% of Area
1	1.567	269970	BB	99.23
2	1.889	1003	bb	0.37
3	2.084	1085	bb	0.40

TKD10 purity: 98.9%

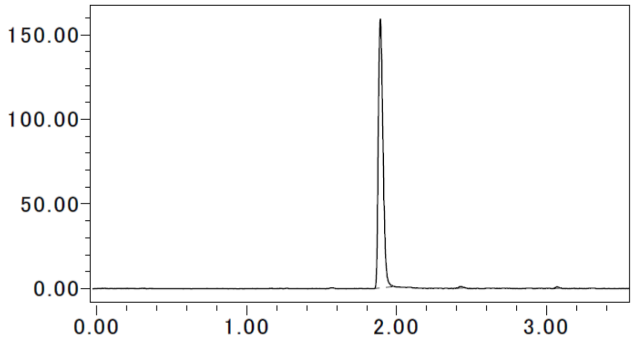

Result of Analysis

	Retention Time (min)	Peak area (μV×sec)	Divisional method	% of Area
1	1.891	345617	BB	98.90
2	2.423	2369	bb	0.68
3	3.070	1484	bb	0.42

TKD125 purity: 99.1%

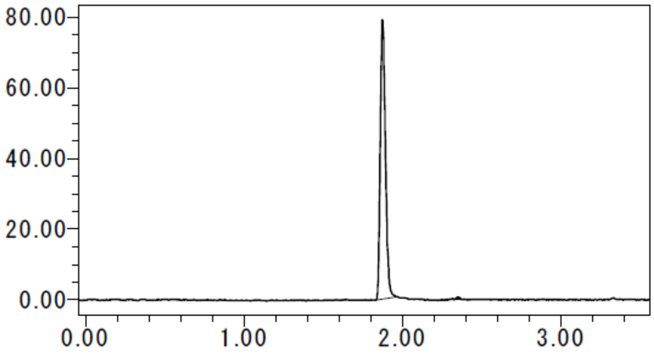

Result of Analysis

	Retention Time (min)	Peak area (μV×sec)	Divisional method	% of Area
1	1.874	177935	BB	99.08
2	2.348	1648	bb	0.92

TKD149 purity: 93.5%

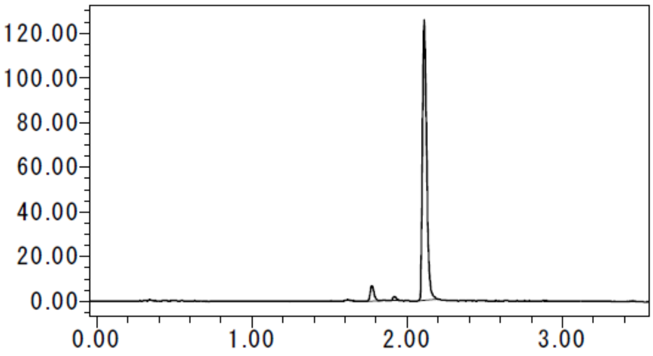

Result of Analysis

	Retention Time (min)	Peak area (μV×sec)	Divisional method	% of Area
1	1.618	957	bb	0.38
2	1.773	11688	BB	4.62
3	1.850	553	bb	0.22
4	1.918	3144	Bb	1.24
5	2.110	236608	BB	93.54

TKD66 purity: 100%

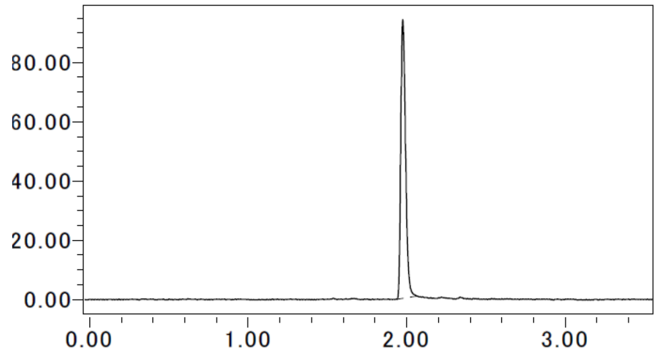

Result of Analysis

	Retention Time (min)	Peak area (μV × sec)	Divisional method	% of Area
1	1.975	197708	BB	100.00

TKD147 purity: 95.2%

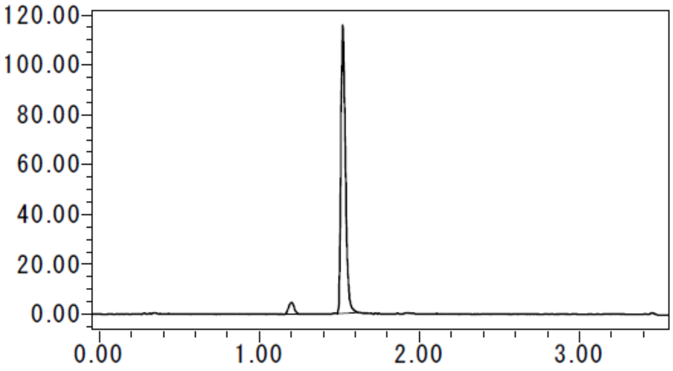

Result of Analysis

	Retention Time (min)	Peak area (μV × sec)	Divisional method	% of Area
1	1.202	11505	BB	4.83
2	1.521	226679	BB	95.17

TKD129 purity: 98.5%

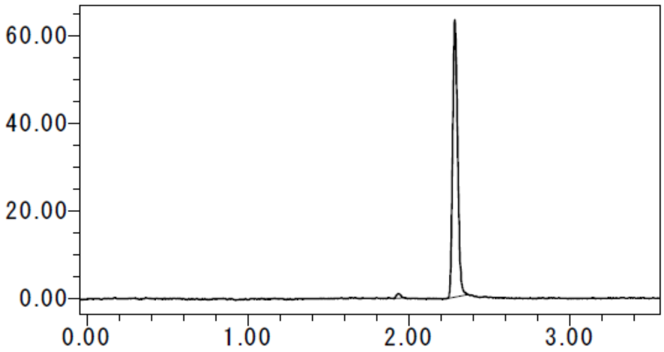

Result of Analysis

	Retention Time (min)	Peak area (μV × sec)	Divisional method	% of Area
1	1.935	2121	bb	1.52
2	2.286	137593	BB	98.48

TKD100 purity: 96.1%

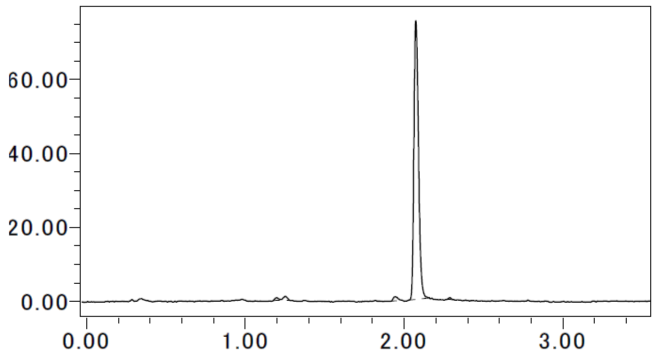

Result of Analysis

	Retention Time (min)	Peak area (μV×sec)	Divisional method	% of Area
1	1.251	3325	bb	2.12
2	1.943	2207	bb	1.41
3	2.074	150958	bB	96.13
4	2.288	540	bb	0.34

TKD146 purity: 100%

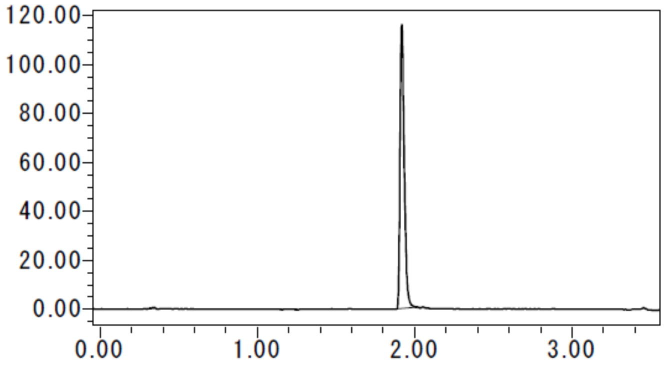

Result of Analysis

	Retention Time (min)	Peak area (μV×sec)	Divisional method	% of Area
1	1.919	217393	BB	100.00

TKD152 purity: 99.2%

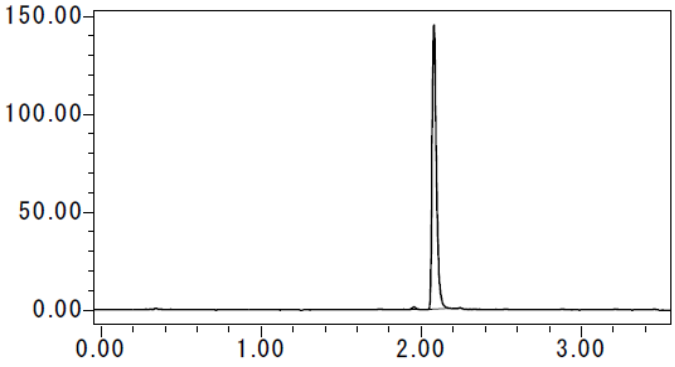

Result of Analysis

	Retention Time (min)	Peak area (μV×sec)	Divisional method	% of Area
1	1.955	2318	bb	0.84
2	2.080	273927	BB	99.16

TKD150 purity: 100%

CoronaCAD

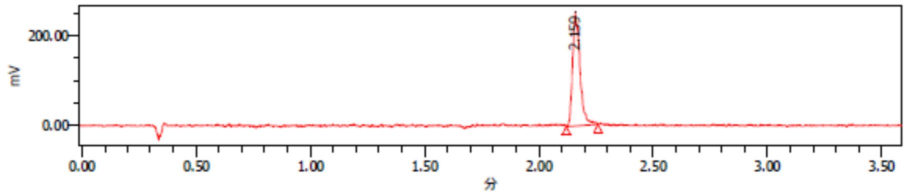

	tR (min)	Area (μV·sec)	%Area
1	2.159	562731	100.00

PDA230nm

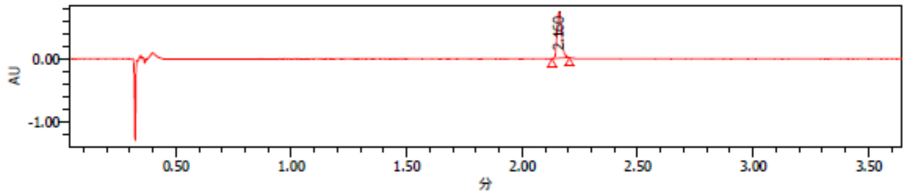

	tR (min)	Area (μV·sec)	%Area
1	2.160	1013041	100.00
